# Investigation of the phytochemical composition, antioxidant, antibacterial, anti-osteoarthritis, and wound healing activities of selected vegetable waste

**DOI:** 10.1038/s41598-023-38591-y

**Published:** 2023-08-10

**Authors:** Mohamed A. Salem, Osama G. Mohamed, Esraa M. Mosalam, Aya Ibrahim Elberri, Hend Mohamed Abdel-Bar, Mariam Hassan, Ahmed A. Al-Karmalawy, Ashootosh Tripathi, Shahira M. Ezzat, Hend E. Abo Mansour

**Affiliations:** 1 Department of Pharmacognosy and Natural Products, Faculty of Pharmacy, Menoufia University, Gamal Abd El Nasr st., Shibīn al-Kawm, 32511 Menoufia Egypt; 2 Pharmacognosy Department, Faculty of Pharmacy, Cairo University, Kasr el Aini St., Cairo, 11562 Egypt; 3 Natural Products Discovery Core, Life Sciences Institute, University of Michigan, Ann Arbor, MI 48109 USA; 4 Biochemistry Department, Faculty of Pharmacy, Menoufia University, Gamal Abd El Nasr st., Shebin El-Koum, 32511 Egypt; 5 Genetic Engineering and Molecular Biology Division, Department of Zoology, Faculty of Science, Menoufia University, Shebin El-Kom, 32511 Menoufia Egypt; 6 Department of Pharmaceutics, Faculty of Pharmacy, University of Sadat City, Sadat City, Egypt; 7 Department of Microbiology and Immunology, Faculty of Pharmacy, Cairo University, Kasr el Aini st., Cairo, 11562 Egypt; 8 Department of Microbiology and Immunology, Faculty of Pharmacy, Galala University, New Galala City, Suez Egypt; 9 Pharmaceutical Chemistry Department, Faculty of Pharmacy, Ahram Canadian University, 6th of October City, Giza, 12566 Egypt; 10 Department of Medicinal Chemistry, College of Pharmacy, University of Michigan, Ann Arbor, MI 48109 USA; 11 Department of Pharmacognosy, Faculty of Pharmacy, October University for Modern Sciences and Arts (MSA), Giza, 12451 Egypt

**Keywords:** Pharmaceutics, Biochemistry

## Abstract

Agri-food wastes, produced following industrial food processing, are mostly discarded, leading to environmental hazards and losing the nutritional and medicinal values associated with their bioactive constituents. In this study, we performed a comprehensive analytical and biological evaluation of selected vegetable by-products (potato, onion, and garlic peels). The phytochemical analysis included UHPLC-ESI-qTOF-MS/MS in combination with molecular networking and determination of the total flavonoid and phenolic contents. Further, the antimicrobial, anti-osteoarthritis and wound healing potentials were also evaluated. In total, 47 compounds were identified, belonging to phenolic acids, flavonoids, saponins, and alkaloids as representative chemical classes. Onion peel extract (OPE) showed the higher polyphenolic contents, the promising antioxidant activity, the potential anti-osteoarthritis activity, and promising antimicrobial activity, especially against methicillin-resistant *Staphylococcus aureus* (MRSA). Furthermore, OPE revealed to have promising in vivo wound healing activity, restoring tissue physiology and integrity, mainly through the activation of AP-1 signaling pathway. Lastly, when OPE was loaded with nanocapsule based hydrogel, the nano-formulation revealed enhanced cellular viability. The affinities of the OPE major metabolites were evaluated against both p65 and ATF-2 targets using two different molecular docking processes revealing quercetin-3,4′-*O*-diglucoside, alliospiroside C, and alliospiroside D as the most promising entities with superior binding scores. These results demonstrate that vegetable by-products, particularly, those derived from onion peels can be incorporated as natural by-product for future evaluation against wounds and osteoarthritis.

## Introduction

The food industry produces tons of waste annually estimated to represent about one-third of the edible food parts made for human consumption^1^. Additionally, the efficient use of agri-food by-products increased tremendously in the last decade due to recent studies relating the dietary consumption of vegetables and fruits with a low incidence of diseases and mortality^2^. Thus, the valorization of agri-food by-products to value-added food and pharmaceutical ingredients has become a significant subject of sustainable food research.

Onion (*Allium cepa* L., F. Liliaceae) is a biennial plant considered one of the most important worldwide vegetable crops that is a part of many dishes, sauces, gravies, salads, and sandwiches^3^. Apart from its culinary uses, *A. cepa* has also been traditionally employed in various indigenous cultures for its therapeutic properties for ages. The ancient Egyptians worshiped the bulb because of its spherical form and concentric rings, which they thought signified eternity, while Greek and Phoenician sailors used it to avoid scurvy and other ailments^4^. Onion has been reported to have antioxidant, anti-inflammatory, antidiabetic, antiallergic, antithrombotic, neuroprotective, anti-hypercholesterolemic, and antimicrobial activitie^5^. Onion phytoconstituents include flavonoids, anthocyanins, carotenoids, terpenoids, oregano-sulfur compounds, vitamins, minerals, carbohydrates, and amino acids. The bulbs, which have a distinctive pungent solid odor, are the main onion edible part, with outer peels that are discarded as a waste product^4^. Onion peels are rich in various bioactive compounds, although phenolic compounds represent the major part and have attracted the interest of researchers and consumers due to their health potential^6^.

Potato (*Solanum tuberosum* L. of the Solanaceae family) is one of the most important vegetable crops that provide edible tubers, consumed as cooked, boiled, or fried^7^. Processing raw tubers, particularly during potato chips manufacturing, usually involves a peeling step that generates bulky waste which is discarded in landfills or used as fertilizer or animal feed^8^. Potato peels are rich in starch, soluble sugars (e.g., glucose, fructose, and sucrose), proteins, dietary fibers, phenolic acids, and glycoalkaloids (e.g., *α*-chaconine and *α*-solanine)^7^. In addition, potato peel extracts have been reported to have antimicrobial, and antioxidant, activities^9^.

Garlic (*Allium sativum* L., family Amaryllidaceae) has been consumed as a part of several dishes or flavoring agent for thousands of years, representing the second most widely cultivated Allium species after onion^10^. Garlic phyto-constituents have been reported to have antioxidant, antimicrobial, anti-inflammatory, antidiabetic, antihypertensive, cardioprotective, hepatoprotective, antifibrinolytic, immunomodulatory and anticancer activities, which are ascribed to a myriad of sulfur-derived bioactive compounds such as alliin, allicin, *S*-allylcysteine and diallyl sulfide^11,12^. Garlic processing industry produces annually million tons of by-products from the straw and peels, offering promising chances for their valorization into environment-friendly alternatives for residues^10,13^.

Vegetable peelings are by-products obtained in high amounts from culinary use and the food industry with limited applications despite their richness in nutraceuticals. Potato, onion, and garlic peels represent valuable vegetable peelings. However, their comparative chemical and biological evaluation has not been explored. In addition, most of the bioactivity studies are limited to the crude extracts not in pharmaceutical drug delivery system^14^. Moreover, most of these extracts suffer from poor aqueous solubility, bioavailability, and physicochemical stability^14^.

Lipid nanocapsule (LNC) is a biomimetic lipid nanocarrier that is composed of a mixture of oil core coated with a shell composed of solid lipids and emulsifier^15^. LNC possess numerous advantages as biocompatibility and biodegradability, small particle size (< 100 nm), ease of manufacturing, physical stability, and high encapsulation efficiency^16^. However, the major drawback of most of nanoparticles is the burst payload release that could compromise therapeutic efficiency and consequently clinical application^17^. The incorporation of nanocarriers in other carrier as hydrogels or scaffold could overcome this challenge^18^. Hydrogel is a semisolid preparation that showed superior potential in different biomedical applications as tissue regeneration, sustained and local drug release^19^. In addition, hydrogel is a 3-D porous matrix that possess high water content and swelling degree^20^. Interestingly, the incorporation of lipid nanocarriers into the hydrogel is supposed to improve the mechanical properties of the hydrogel as well as prolong drug release^21^.

In this context, potato, onion, and garlic peels were studied regarding their chemical compositions and total antioxidant capacity. Moreover, the chemical composition was characterized by HPLC coupled with MS^2^ analysis. Consequently, in vitro caractrization, assaying different bioactivities, including antimicrobial, anti-osteoarthritis, and wound healing activities were also conducted. Onion peel extract was loaded into lipis capsule based hydrogel revealing enhanced cellular viability.

## Material and methods

### Plant material and extraction

Onions bulbs (*Allium cepa* L.) of the yellow cultivar (Giza 20 variety), potato tubers (*Solanum tuberosum*, Krozo variety) and Garlic bulbs (*Allium sativum*, white baladi variety) were provided in October 2021 from Local Factory for Dehydrated vegetables (Menoufia Governorate, Egypt). Permission to work on and to collect the plant material comply with the institutional guidelines which were obtained from Phamacognosy and Natural Products Department, Faculty of Pharmacy, Menoufia University, Egypt. The fully grown bulbs/ tubers were sorted, manually-peeled and the peels were air dried. Voucher specimens were deposited, after inspection by plant taxonomy specialist, at the herbarium of Phamacognosy and Natural Products Department, Faculty of Pharmacy, Menoufia University (202110-20). Peels were powdered using a laboratory mill and the ground dried powder (100 g, for each replicate) was homogenized with 1 L of cold 80% methanol using an ultrasonic bath for 30 min. Extracts were then filtered and concentrated under reduced pressure in a rotary evaporator at 40 °C^22^. The obtained dried extracts were then further subjected to chemical characterization and biological tests.

### UHPLC–QTOF-MS/MS analysis and molecular networking

#### UHPLC-QTOF-MS/MS analysis of crude extracts

Ultra-high-performance liquid chromatographic (UHPLC) analysis was performed on an Agilent system composed of an Agilent 1290 Infinity II UHPLC coupled to an Agilent 6545 ESI-Q-TOF-MS in both negative and positive modes^23^. Methanolic crude extracts were suspended in methanol at 1 mg/mL concentration and aliquots (1 µL) of each extract was analysed using the following conditions: Kinetex phenylhexyl (1.7 µm, 2.1 × 50 mm) column, 1 min isocratic elution of 90% A (A: 100% H_2_O + 0.1% formic acid) followed linear gradient elution to 100% B (95% MeCN + 5% H_2_O + 0.1% formic acid) over 6 min with a flow rate of 0.4 mL/min. ESI conditions were set with the capillary temperature at 320 °C, source voltage at 3.5 kV and a sheath gas flow rate of 11 L/min. Ions detected in the full scan at an intensity above 1000 counts at 6 scans/s, with an isolation width of 1.3 ~ m/z, a maximum of 9 selected precursors per cycle and using ramped collision energy (5 × *m/z*/100 + 10 eV). Purine C_5_H_4_N_4_ [M + H]^+^ ion (m/z 121.0508) and hexakis (1H,1H,3H-tetrafluoropropoxy)-phosphazene C_18_H_18_F_24_N_3_O_6_P_3_ [M + H]^+^ ion (m/z 922.0098) were used as internal lock masses for positive mode while TFA C_2_HF_3_O_2_[M–H]^−^ ion (m/z 112.9855) and hexakis(1H,1H,3H-tetrafluoropropoxy)-phosphazene C_18_H_18_F_24_N_3_O_6_P_3_ [M + TFA–H]^−^ ion (m/z 1033.9881) were used as internal lock masses for negative mode.

The mzXML files were imported and processed with MZmine 2 v2.53^24,25^ with the following previously published parameters^26^: (1) Mass Detection: MS^1^ noise level, 1E3; MS^2^ noise level, 1E2. (2) ADAP chromatogram builder: MS-level, 1; min group size in no. of scans, 2; group intensity threshold, 2E4; min highest intensity, 5E3; *m*/*z* tolerance, 0.01 m*/z*. (3) Chromatogram deconvolution: Local minimum search algorithm (4) Isotopic peaks grouper: *m*/*z* tolerance, 0.01 m*/z*; RT tolerance, 0.05 min; monotonic shape, yes; maximum charge, 2; representative isotope, lowest *m/z*. (5) peak alignment: *m*/*z* tolerance, 0.02 m*/z*; weight for *m/z*, 75; RT tolerance, 0.2 min; weight for RT, 25. (6) Peak list rows filter: Only features with accompanying MS^2^ data and their retention time are between 0 and 7.5 min were kept. (7) Duplicate peak filter: filter mode, old average; *m*/*z* tolerance, 0.02 m/*z*; RT tolerance, 0.5 min. The resulting feature lists were exported to the GNPS-compatible format, using the dedicated “Export for GNPS” built-in options^27^.

#### GNPS feature-based molecular MS/MS network

A Feature-based Molecular Network (FBMN) was created by processing the output of MZmine 2 utilizing FBMN workflow (version release_28.2)^28^ on GNPS webserver. The following parameters were adapted: MS^2^ spectra were filtered by removing all MS^2^ fragment ions within ± 17 Da of the precursor *m/z*, and only the top 5 fragment ions in the ± 50 Da window through the spectrum were utilized. Both the MS^2^ fragment ion tolerance and the precursor ion mass tolerance were set to 0.02 Da. Nodes are connected in the molecular network if there is a cosine score above 0.7 and more than 5 matched peaks. Moreover, nodes were only kept in the molecular network if each of these nodes appeared in each other's top 10 most similar nodes. The maximum size of clusters in the network was set to 100. Additionally, the MS^2^ spectra were searched against GNPS public spectral libraries to find matches having a score above 0.7 and at least 5 matched peaks. The molecular networks were visualized using Cytoscape software v.3.9.1.^29^.

### Determination of antioxidant activity

For the determination of the antioxidant activity, 2,2-diphenyl-1-picryl-hydrazyl-hydrate (DPPH) method was adopted^30,31^. Briefly, a stock solution of each extract (10 mg/mL in methanol) was used to prepare a serial dilution (1000–10 µg/mL in methanol). A stock solution of trolox (20 μg/mL in methanol) was used to prepare a serial dilution. In 96 well plate, 100 μL of each sample was added to 100 μL freshly prepared DPPH reagent (0.1% in methanol) and the plate was incubated in dark for 30 min at room temperature (25 °C). The color intensity was measured at 540 nm using microplate reader FluoStar Omega and the resulting data were represented as means ± SD for three biological replicates according to the following equation: percentage inhibition = ((Average absorbance of blank-average absorbance of the test)/(Average absorbance of blank))*100. The ferric reducing antioxidant power (FRAP) assay was also performed^32^. Briefly, a stock solution of each extract (10 mg/mL in methanol) was used to prepare a serial dilution (1000–10 µg/mL in methanol). A stock solution of trolox (2 mM in methanol) was used to prepare a serial dilution (1000–25 µM). In 96 well plate, 10 μL of each sample was added to 190 μL freshly prepared TPTZ reagent (300 mM Acetate Buffer (pH = 3.6), 10 mM TPTZ in 40 mM HCl, and 20 mMFeCl_3_, 10:1:1 v/v/v, respectively) and the plate was incubated in dark for 30 min at room temperature (25 °C). The color intensity was measured at 593 nm using microplate reader FluoStar Omega and the resulting data were represented as means ± SD for three biological replicates.

### Determination of total phenolics and flavonoids contents

Total phenolics content was determined using the Folin–Ciocalteu method^33^. Briefly, a stock solution of each extract (10 mg/mL in methanol) was used to prepare a serial dilution (1000–10 µg/mL in methanol). A stock solution of gallic acid (1 mg/mL in methanol) was used to prepare a serial dilution. In 96 well plate, 10 μL of each sample was added to 100 μL of Folin-Ciocalteu reagent (Diluted 1: 10) reagent and 80 μL of 1 M sodium carbonate. The plate was incubated in dark for 20 min at room temperature (25 °C). The color intensity was measured at 630 nm using microplate reader FluoStar Omega and the resulting data were represented as means ± SD for three biological replicates and the results were presented as μg gallic acid equivalent/mg extract. Total flavonoids content was determined using the aluminum chloride method^34^. Briefly, a stock solution of each extract (10 mg/mL in methanol) was used to prepare a serial dilution (1000–10 µg/mL in methanol). A stock solution of rutin (200 μg/mL in methanol) was used to prepare a serial dilution. In 96 well plate, 15 μL of each sample was added to 175 μL of methanol, 30 μL of freshly prepared 1.25% AlCl_3_ reagent and 30 μL of 0.125 M sodium acetate. The plate was incubated in dark for 5 min at room temperature (25 °C). The color intensity was measured at 420 nm using microplate reader FluoStar Omega and the resulting data were represented as means ± SD for three biological replicates and the results were presented as μg quercetin equivalent/mg extract.

### Mouse chondrocyte cell cultures

Mouse-isolated chondrocyte cell line cells, which were obtained from American Type Culture Collection, were cultured using DMEM (Invitrogen/Life Technologies) supplemented with 10% FBS (Hyclone), 10 µg/mL of insulin, and 1% penicillin–streptomycin. Cells were plated prior to incubation at 37 °C in a humidified 5% CO_2_ incubator. After growing to 80%–90% confluence, cells were harvested with 0.25% (w/v) trypsin—0.53 mM EDTA solution. To explore the anti-osteoarthritis activity, chondrocytes were treated for 24 h with IL-1β (10 ng/mL) following pretreatment with serial concentrations tested extracts for 48 h at 37 ºC, then the plates are to be examined under the inverted microscope and proceed for the MTT assay.

### MTT cytotoxicity assay protocol

The method of monitoring in vitro cytotoxicity was performed using MTT (3-[4,5-dimethylthiazol-2-yl]-2,5 diphenyltetrazolium bromide) assay (in vitro toxicology assay kit MTT-based, Sigma-Aldrich, Inc.) by determining the cell number spectrophotometrically as a function of mitochondrial activity in living cells. Initially, chondrocytes cells in the log phase of growth were plated into 96-well plates (5 × 10^3^ cell/cm^2^) for 24 h, after which serial concentrations tested extracts was added for 48 h. Afterwards, 10 μL of MTT (final concentration of 5 mg/mL) solution was added for 4 h at 37 °C. The cytotoxic effect of the tested extracts, dissolved in DMSO, on chondrocytes was determined at various concentrations against the blank (DMSO alone). The absorbance was measured spectrophotometrically at a wavelength of 570 nm. The experiments were performed in triplicates.

### Enzyme-linked immunosorbent assay (ELISA) and quantitative real-time polymerase chain reaction (qRT-PCR)

Nitric oxide was measured colorimetric using nitric oxide assay kit (Abcam, China, kit no ab65328). Collagen II was determined using Enzyme-linked Immunosorbent Assay (ELISA) for quantitative detection (Novus Biologicals kits). Matrix metalloproteinase 13 (MMP-13) was determined using in vitro SimpleStep ELISA® kit (Abcam, China, kit no. ab270216). NF-κB p65(Nuclear Factor Kappa B p65) was measured using Elabscience® ELISA kit. All assays were performed according to the manufacturer's instruction. All assays were performed three times.qRT-PCR was performed using Qiagen RNA extraction (Qiagen, GmbH, Hilden, Germany) and the iScript One-Step RT-PCR Kit with SYBR® Green, Bio-Rad) using reader Rotorgene RT- PCR system, according to manual instructions^35^. The sequences of the primers used are presented in Table S1.

### Screening the antibacterial activity

The antibacterial activity of the tested extracts was screened against a group of Gram-positive and Gram-negative highly virulent pathogens (methicillin-resistant *Staphylococcus aureus (MRSA USA300)*, *Acinetobacter baumannii AB5075*, *Escherichia coli ATCC8* and *Pseudomonas aeruginosa PAO1*)^36–37^. The minimum bactericidal concentration (MBC) of the extracts was determined using the broth microdilution method as described before^38,39^. The experiment was performed three independent times and the MBC was recorded as mean ± standard deviation.

### Preparation of onion peel extract (OPE) nano-formulations and in-vitro characterization of the prepared onion peel extract lipid nanocapsule

Onion peel extract (OPE) loaded Lipid nanocapsule (LNC_OPE_) was fabricated using phase inversion method^40,41^. Briefly, oil-surfactant mixture composed of labrafac (Gattefossé S.A. (Saint-Priest, France): Kolliphor® HS15 (Sigma-Aldrich) in ratio of 1:2 w/w was mixed with epikuron 200® (Cargill, Minneapolis, MN, USA) (1.5% w/w) using magnetic stirrer at 500 rpm for 30 min. Consequently, onion peel extract was accurately weighed and dissolved in the prepared oil-surfactant dispersion (10% w/w). Afterwards, the obtained mixture was titrated with equivolume of sodium chloride solution (1.75% w/w). LNC_OPE_ was prepared using three thermal-cooling cycles, each for between 70 and 40 °C under continuous magnetic stirring (500 rpm). In the last cycle, the obtained mixture was diluted with cold deionized water (10 mL, 4 °C) to aid the formation of LNC_OPE_.

The particle size and size distribution measured as polydispersity index (PDI) of the fabricated LNC_OPE_ were dynamic light scattering technique using Nanosizer ZS Series (Malvern Instruments Ltd., UK). Surface charge as zeta potential was measured using electrophoresis. Accordingly, LNC_OPE_ was diluted with deionized water (1: 100 v/v) then transferred to disposable plain folded capillary Zeta cells. All the measurements represent the average of 20 runs, each run was completed in triplicate at 25 °C using an angle of 90°^42^.

The entrapment efficiency (EE%) was determined directly by measuring the encapsulated quantity of the extract. Briefly, the amount of the unentrapped extract in LNC_OPE_ was separated with ultrafiltration centrifugation using MWCO 100 K at 14,000 rpm for 45 min. The obtained pellets were dissolved in dimethylformamide (10 mL). Onion peel extract was quantified using a previously reported HPLC method. Briefly, the HPLC system was composed of Dionex ultimate 3000 controlled by Chromeleon Chromatography Data System with UV detector and quaternary pump (Thermo Fisher Scientific, USA). A reverse phase C18 column (Thermo BDS, 250 × 4.6 mm, 5 μm) was used for onion peel extract separation at detection wavelength of 360 nm at 40 °C. The mobile phase composed eluent A (0.1% v/v formic acid in water) and eluent B (0.1% v/v formic acid in acetonitrile) at flow rate 1 mL/min. The gradient elution was 100% A at time 0, 97% A at 0–5 min, 50% A 5–45 min, 0% A at 45–50 min^43^. The EE % was calculated using the following equation:

1 $${\text{EE }}\% \, = { }\frac{{{\text{Amount }}\;{\text{of}}\;{\text{ red}}\;{\text{ onion}}\,{\text{ peel}}\;{\text{ extract }}\;{\text{determined }}\;{\text{in }}\;{\text{the}}\;{\text{ LNC}}}}{{{\text{Total}}\;{\text{ amount }}\;{\text{of }}\;{\text{red }}\;{\text{onion}}\;{\text{ peel }}\;{\text{extract }}\;{\text{added }}}} \times {1}00$$

LNC_OPE_ was visualized using transmission electron microscope (TEM, Jeol, JEM-1230, Japan). One drop of the LNC_OPE_ was deposited on a copper 300-mesh grid, coated with carbon for 10 min after which, any excess fluid was dried with a filter paper. The sample was negatively stained with one drop of 1% phosphotungstic acid, applied, and allowed to dry for 5 min^44^.

### Preparation and characterization of onion peel extract loaded nanocapsule based hydrogel

Extract loaded LNC based hydrogel was prepared by dispersing the LNC_OPE_ into carbopol 940 (Lubrizol Advanced Materials, Inc, Ohio, USA) (1% w/v) under continuous stirring (500 rpm) for 45 min. Afterwards, triethylamine (0.5% w/w) was added to neutralize the mixture and form the gel^45,46^. The pH of the fabricated LNC_OPE_ based hydrogel was measured using a pH meter (Jenway, UK). Physical appearance includes color, homogeneity and texture were inspected visually^46^.

The viscosity of the proposed LNC_OPE_ based hydrogel was measured by using Brookfield rheometer (Brookfield DV-III ultra-programmable cone and plate rheometer fitted with a spindle number 52 and controlled with Brookfield Rheocalc operating software, USA). Briefly, 1 g of the prepared LNC_OPE_ based hydrogel was placed into the rheometer and the viscosity was measured as a function to the shear rate^47^.

The spreadability of the prepared LNC_OPE_ based hydrogel was assessed using glass slide method^46^. About 0.5 g of the prepared LNC_OPE_ based hydrogel was placed in the center of glass slide with a circle mark of 1 cm diameter^48^.Consequently, LNC_OPE_ based hydrogel was squeezed by another glass slide where a 500 g standard weight was placed on the glass slides. Afterwards, the diameter of the gel was measured and the spreadability was calculated using the following equation:

2 $${\text{Spreadability}}\;{\text{index}}\; = \;d^{2} *{ }\frac{{\uppi }}{{4{ }}}$$

The swelling index of LNC_OPE_ based hydrogel was measured using the gravimetric method^18^. LNC_OPE_ based hydrogel (1 g) was lyophilized for 48 h then immersed in phosphate buffer (pH 5.5). The swollen hydrogel was removed from the medium and weighed at different time intervals for 24 h. Swelling ratio was calculated using the following equation:

3 $${\text{ Swelling}}\;{\text{ratio}}\;{ = }\;\frac{{W_{{w - W_{i} { } }} }}{{W_{i} { }}}$$

### In-vitro onion peel extract release

The in vitro extract release from the prepared LNC_OPE_ and LNC_OPE_ based hydrogel was performed using dialysis method. An aliquot of each formula equivalent to 5 mg onion peel extract was placed in dialysis bag (cut-off 10–12 KDa) and immersed in 100 mL of PBS (pH 5.5) at 37 ± 0.1 °C in a thermostatically controlled shaking water bath at 50 strokes/min. At predetermined time intervals up to 24 h, 1 mL of the release medium was withdrawn and the onion peel extract was quantified using the previously reported HPLC method^45^.

### In-vitro experiment

#### Cell line and determination of cytotoxicity and cell viability

Hfb4 normal skin cells was purchased from The Egyptian Organization for Biological Products and Vaccines (Vacsera, Cairo, Egypt). The cells were grown in DMEM low glucose medium supplemented with 1% penicillin/streptomycin and 10% fetal bovin serum; which were purchased from Serana Europe GmbH (Germany). The cells were maintained at 37 °C and 5% CO_2_. MTT (3-(4,5-dimethylthiazol-2-yl)-2,5-diphenyltetrazolium bromide) kit was purchased from Elabscience® (USA) and was performed according to the supplier’s instructions. Free OPE hydrogel, LNC_OPE_ hydrogel, and their blanks were tested for their cytotoxicity and effect on cell viability. The following concentrations from the formulations and their blanks were used: 3, 1.5, 0.75, 0.37, 0.18, and 0.09 mg/mL to determine which concentration will have the lower cytotoxicity and best cell viability.

#### Determination of inflammatory markers and oxidative stress biomarkers

The lipopolysaccharide (LPS) was purchased from Merck (USA), and it was used to induce inflammation as well as oxidative stress. Accordingly, there were six different groups for these tests; normal Hfb4 cells, LPS only, the two formulations along with their blanks with LPS. First, the LPS was added to the cells at a concentration of 10 µg/mL^49^ and left for 24 h incubation with the cells. Afterward, the formulations and their blanks were added to the inflamed cells at a concentration of 3 mg/mL, based on the results of MTT assay, and left for another 24 h. Finally, interleukin 6 (IL-6) and interleukin 1 beta (IL-1β) were determined in the cells by commercial ELISA kit from Elabscience® (USA) according to the supplier’s guidelines.

Malondialdehyde (MDA) and glutathione (GSH) were detected in the cells using commercial colorimetric assay kit according to the manufacturer’s instructions (Elabscience®, USA). The groups were divided as in the inflammatory markers.

### Experimental animals and in-vivo wound healing activity

#### Animals, study design and determination of wound contraction

Seventy five male albino mice aging 6–8 weeks and weighing 30–35 g were obtained from Vacsera, Egypt. The mice were accommodated for two weeks on water supply, commercial diet, and light/dark cycles. Animal sheltering and handling were according to the Guide for the Care and Use of Laboratory Animals (National Research Council) and the proposal was approved by Research Ethical Committee, Faculty of Science, Menoufia University, Egypt; FGE1020. All animal experiments were performed in accordance with guidelines and regulations for the care and use of experimental animals. Authors have complied with the ARRIVE guidelines for reporting. The experimental mice were arbitrarily divided into five different groups with 15 mice in each group as follows: untreated, blank 1, blank 2, free OPE hydrogel, and LNC_OPE_ hydrogel. All the treatments were applied topically to the wound area daily for 12 consecutive days. The untreated control group did not receive any treatment.

The mice were anesthetized with pentobarbital (70 mg/kg i.p.). A circular area of about 500 mm^2^ on the back of the mice was marked and cut carefully. The wound area was traced by a graph paper on days 1, 3, 6, 9, and 12. The changes in the dimensions of the wound were recorded and the rate of wound contraction was calculated according to the following formula: $\frac{Healed \;area}{{Total \;wound\; area}} \times 100$, where healed area = original wound area−current wound area^50^. The wounds were photographed on the same days by a digital camera.

#### Histopathological investigation, immunohistochemistry and qRT-PCR for genes’ expression

The day of wounding was designated to be day zero. Three representative mice from each group were sacrificed under pentobarbital anesthesia on day 1, 3, 6 and 9 to collect wound skin samples for the histopathological examination and immunohistochemistry. The remaining mice were sacrificed on day 12 to obtain wound skin samples for the same measurements and also for qRT-PCR. The samples for histopathological examination and immunohistochemistry were fixed in 7% formaldehyde.

The samples were cut into small sections; about 5 mm. Afterward, part of the samples was stained with hematoxylin and eosin (H & E) to detect histological changes. The other part was stained with Masson’s trichome (MT) staining to detect collagen deposition. The images were obtained with an Olympus microscope (Japan) equipped with a digital camera.

Immunohistochemistry was performed to identify tumor necrosis factor-alpha (TNF-α) and alpha-smooth muscle actin (α-SMA) by corresponding antibodies. In order to generate quantitative measures, the optical density (OD) of TNF-α and α-SMA was determined.

The expression level of activating transcription factor 2 (ATF-2), Fos proto-oncogene (c-Fos), Fos-related antigen 2 (Fra-2), and Jun proto-oncogene (c-Jun) was determined by qRT-PCR. Total RNA was extracted from wound skin tissue homogenate by RNeasy Mini kit (Qiagen, Germany). cDNA was obtained by EasyScript® First-Strand cDNA Synthesis SuperMix (TransGen Biotech Co., China). The amplification was done by QuantiTect® SYBR® Green PCR (QIAGEN, Germany) using StepOnePlus™ Real-Time PCR system (Thermo Fisher Scientific, USA). The sequence of the used primers was constructed by Primer-Blast and were purchased from Macrogen (Korea) (Table S2). Glyceraldehyde- 3-phosphate dehydrogenase (GAPDH) was used as a reference gene and the results were expressed as mean fold change relative to the normal control group.

#### Molecular modeling

The most abundant compounds identified from the OPE were evaluated against both p65 and ATF-2 targets using two different molecular docking processes using the MOE program^51,52^. The structure of the identified compounds were obtained from the PubChem website. They were introduced individually into the MOE working screen, prepared^53,54^, and inserted into a single database to be ready for the docking process. The X-ray structures of p65 and ATF-2 target proteins (PDB ID:1RAM^55^ and 6ZR5^56^, respectively) were obtained from the Protein Data Bank. Each protein was prepared individually following the full preparation steps described earlier^57,58^. Two different docking processes were performed for the same database prepared previously according to the methodology illustrated earlier^59,60^. One pose for each compound was selected to be further investigated based on the obtained scores and RMSD values^61,62^.

#### Statistical analysis

The data were analyzed by One-way analysis of variance (ANOVA) followed by Tukey post hoc test using IBM® SPSS® Statistics version 22 software (IBM Corp., NY, USA). Data were presented are means ± standard deviations, *p* < 0.05 as the significance threshold. Heatmap was generated using Metaboanalyst^63^.

## Results

### Phytochemical characterization of metabolites by UHPLC–QTOF–MS/MS supported by molecular networking

**Figure 1:**
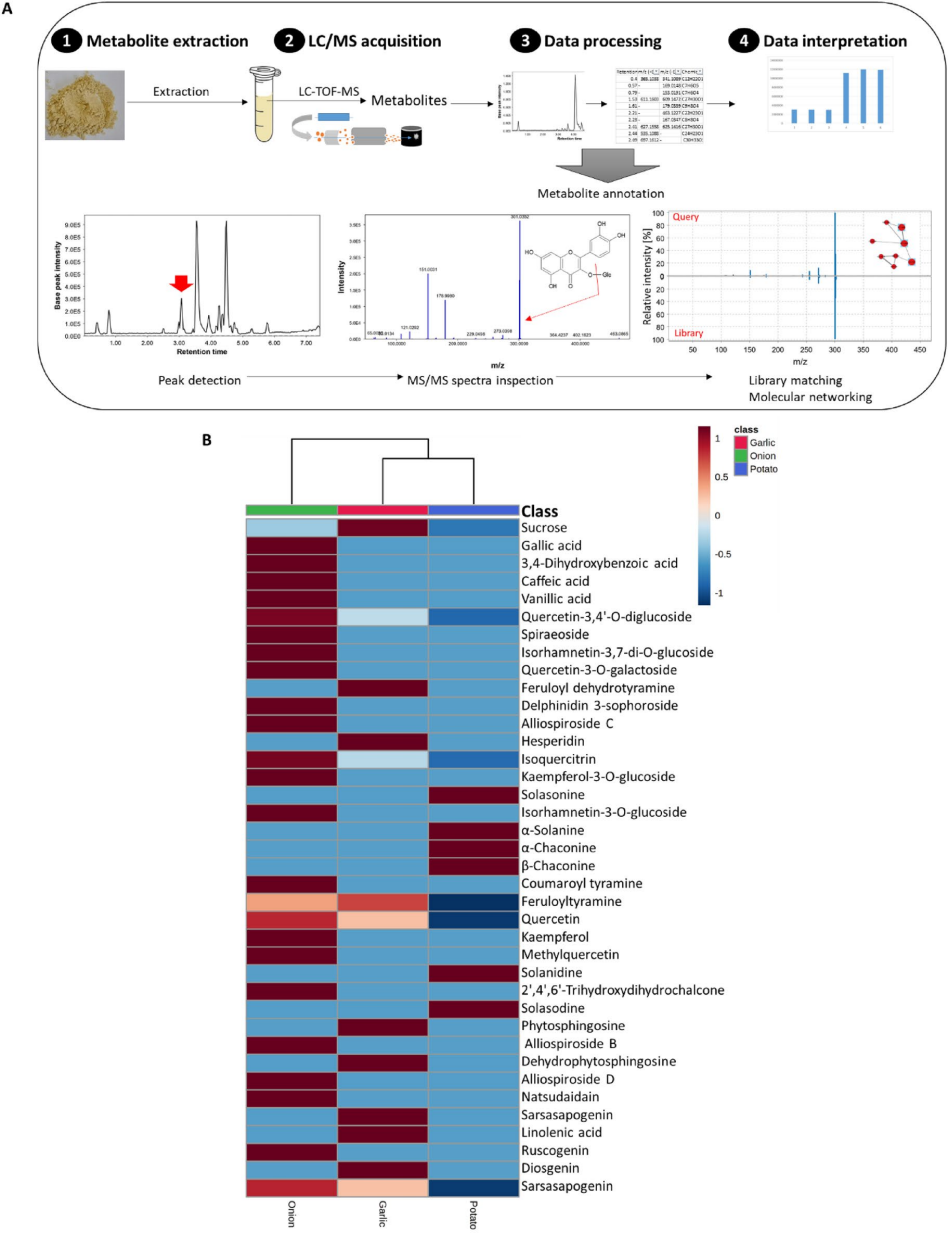
Metabolomics workflow (**A**) and heat map (**B**) of identified metabolites from selected peel extracts as analyzed by LC/MS^2^.

An Ultra-high performance liquid chromatography–quadrupole time-of-flight mass spectrometry (UHPLC–QTOF-MS/MS) method was used to analyze the metabolites extracted from the tested peel extracts (Fig. 1). Both positive and negative MS ionization modes were performed to increase metabolite coverage from their preferred mode. A total of 47 compounds were tentatively identified by investigating their elution pattern, molecular ions, detected adducts, fragmentation pattern and MS/MS-based molecular networks compared to literature data (Table S3). The most representative chemical classes were phenolic acids, flavonoids, saponins, and alkaloids.

Total ion chromatograms (TIC) of garlic peel extract (Fig. S1) revealed the presence of a small peak at 3.44 min in negative ionization mode. The extracted ion chromatogram showed a molecular ion peak at 312.1240 m/*z* (Fig. S2). The MS/MS analysis revealed the presence of fragments at *m*/*z* 297.0995, 190.0512, 178.0513 and 148.0533. These fragments matched the characteristic fragments for feruloyltyramine with the GNPS library^64^. Using the same protocol, the annotation of the flavonoid aglycone, quercetin, was confirmed in both the negative (Fig. S3) and positive (Fig. S4) ionization modes.

Further, the disaccharide sugar, sucrose, showed the characteristic fragments in both negative (Fig. S5) and positive (Fig. S6) ionization modes. Additionally, the identification of several compounds was confirmed from the molecular networks performed on the analyzed data (Fig. S7). The identification of characteristic alkaloids such as solasonine, *α*-solanine, *α*-chaconine, *β*-chaconine, solanidine, and solasodine, particularly from potato peel extract, was confirmed from the positive ionization mode (Figs. S8–10). Additionally, the identification of these compounds was by the molecular networks performed on the analyzed data (Figs. S11–13).

A heat map analysis based on the abundance (relative peak areas) of the detected metabolites was performed to examine metabolome variation in the tested peel extracts (Fig. 1). Onion peel extract contained high levels of metabolites from phenolic classes such as flavonoids, anthocyanins, and phenolic acids. Saponins (e.g. alliospiroside B, alliospiroside D, ruscogenin and sarsasapogenin) also dominated the metabolome of onion peels. Whereas diosgenin, sarsasapogenin, feruloyltyramine, and quercetin were detected in high levels in garlic peel extract. Additionally, alkaloids (e.g. solasonine, solasodine, *α*-solanine, *α*-chaconine and *β*-chaconine) uniquely characterized potato peel extract. Phenolic acids and flavonoids were either not abundant or not detected in garlic and potato peel extracts.

### Antioxidant potential and total phenolic contents

Two different methods, namely determined the antioxidant activity; 2,2-diphenyl-1-picryl-hydrazyl-hydrate (DPPH) and the ferric reducing antioxidant power (FRAP) assay. The results revealed that onion peels showed the highest antioxidant potential, followed by garlic peel extracts (Fig. S14). Conversely, the least activity was observed for potato peel extract. Interestingly, the results of the two assays were found to be positively correlated (Table 1). The promising antioxidant activities encouraged us to further evaluate the phenolics contents. Recent studies suggest the protective effects of phenolic compounds in the management of osteoarthritis, alleviating chondrocyte inflammation and providing therapeutic solutions for chronic wound care^65,66^. Therefore, the total phenolics and flavonoids contents were determined spectrophotometrically using the Folin–Ciocalteu and aluminum chloride methods. The results of this analysis revealed that onion peels showed the highest phenolics content (196.45 ± 8.83 μg gallic acid/mg extract) and flavonoids content (397.03 ± 21.27 μg rutin equivalent /mg extract), followed by garlic peel extract. On the contrary, the least activity was observed for potato peel extract. Interestingly, the content of total phenolics was positively correlated with antioxidant capacity (Table 1).

**Table 1:** Pearson’s correlation coefficients indicating the relationship between antioxidant activity and phenolics contents.

	DPPH	FRAP	Phenolics	Flavonoids
DPPH	1	–	–	–
FRAP	0.992^#^	1	–	–
Phenolics	0.996^#^	0.996^#^	1	–
Flavonoids	0.987^#^	0.999^#^	0.994^#^	1

^#^Correlation significant at p < 0.001 (ANOVA).

### Effect of tested extracts on chondrocyte viability and inflammatory mediators in IL-1β-stimulated chondrocytes

**Figure 2:**
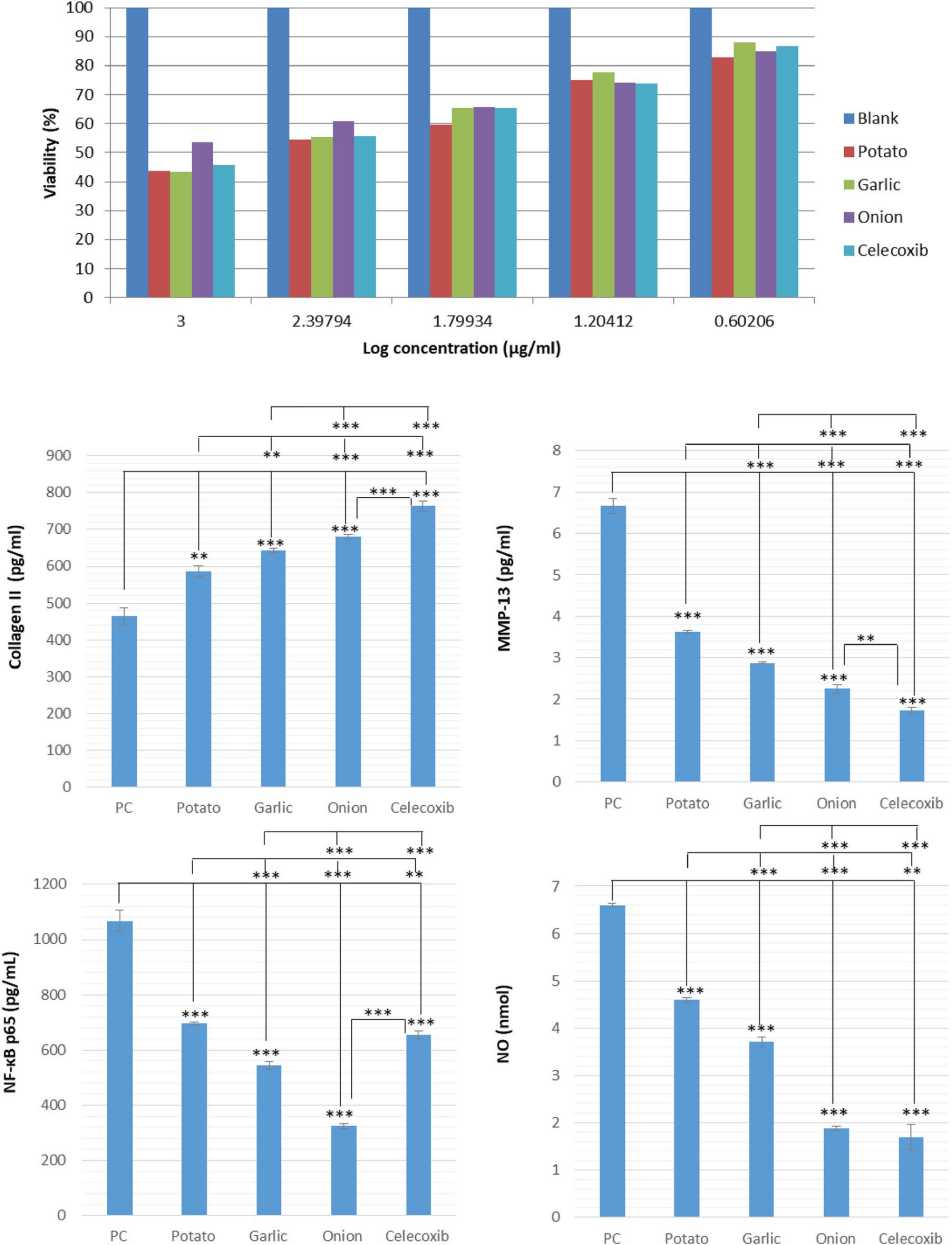
Effects of selected peel extracts on chondrocyte viability and IL-1β-induced extracellular matrix degradation and inflammatory mediators. The cytotoxic effect of the tested extracts, dissolved in DMSO, on chondrocytes was determined at various concentrations against the blank (DMSO alone). To explore the anti-osteoarthritis activity, chondrocytes were treated for 24 h with IL-1β (10 ng/mL) following pretreatment with serial concentrations of the tested extracts for 48 h at 37 °C. Positive control (PC) is the chondrocytes treated for 24 h with IL-1β (10 ng/mL) only. Levels of nitric oxide (NO) was measured colorimetric using nitric oxide assay kit. Collagen II, matrix metalloproteinase 13 (MMP-13) and NF-κB p65(Nuclear Factor Kappa B p65) were determined using Enzyme-linked Immunosorbent Assay (ELISA). Data are presented as the mean ± S.D. of three independent experiments. Asterisks indicate significance level (*, **, ***p < 0.05, 0.01 and 0.001, respectively).

The cytotoxicity of the tested extracts from potato, onion, and garlic peels on the chondrocytes was tested by MTT assay (Fig. 2). After treatment with different concentrations of the tested extracts (0–2000 μg/mL), onion peel extract showed the highest viability with IC_50_ = 1607 ± 78.8 µg/mL, when compared to celecoxib (IC_50_ = 527.1 ± 25.9 µg/mL). On the other hand, potato and garlic peel extracts showed similar viability at lower concentrations (IC_50_ = 396.9 ± 19.5 and 451.2 ± 22.1 µg/mL, respectively). These results indicate that the tested extracts were, at lower concentrations, relatively non-toxic to the cells compared to blank (DMSO).

Further, we assessed the impact of tested peel extracts on IL-1β-induced inflammation in mouse-isolated chondrocytes. We measured chondrocyte expression of key osteoarthritis-associated factors such as matrix metalloproteinase 13 (MMP-13), nitric oxide (NO), collagen II, nuclear factor kappa-light-chain-enhancer of activated B cells p65 (NF-κB p65) as well as the expression of inflammatory mediators in chondrocytes such as interleukin 6 (IL-6), tumor necrosis factor-α (TNF-α), cyclooxygenase-2 (COX-2), and inducible nitric oxide synthase (iNOS).

Our results showed that onion peel extract (OPE) showed the most significant down-regulation of MMP-13, NO, and NF-κB p65 when compared to PC (positive control is the chondrocytes treated for 24 h with IL-1β (10 ng/mL) only) (Fig. 2). The effect on NF-κB p65 was better than celecoxib, while the effect on NO was similar to the standard drug. Garlic peel extract showed higher activity than potato peel extract. However, the activity was significantly (p < 0.001) lower than onion peels or the standard celecoxib. All the tested extracts significantly reduced MMP-13, NO, and NF-κB p65 levels compared to IL-1β-treated chondrocytes (p < 0.001).

Moreover, collagen II levels, which were downregulated by IL-1β treatment, were significantly induced by all tested extracts. OPE reversed collagen II degradation causing its upregulation better than potato and garlic peel extracts, still lower than celecoxib (Fig. 2). Further, the overproduction of IL-6, TNF-α, COX-2, and iNOS induced by IL-1β were all significantly inhibited (p < 0.001) by the tested peel extracts (Fig. S15). Our results showed that OPE showed the most significant downregulation of IL-6, TNF-α, COX-2, and iNOS. The effect on IL-6 and TNF-α was better than celecoxib, while the effect on COX-2 was similar to the standard drug.

### Screening of the tested extracts for the antibacterial activity

The antibacterial activity of the tested extracts was determined using minimum bactericidal concentration (MBC). All the tested extracts showed bactericidal activity against all the tested pathogens except potato extract did not show any bactericidal activity against MRSA USA300 and *A. baumannii AB5075* (Table 2)*.* OPE recorded the highest bactericidal activity against MRSA USA300 and *A. baumannii AB5075* with MBC 3.125 ± 0 mg/mL and 4.167 ± 1.8 mg/mL, respectively. Potato extract recorded the highest bactericidal activity against *P. aeruginosa PAO1* with MBC 6.25 ± 0 mg/mL.

**Table 2:** Antibacterial activity of the tested peel extracts.

Peel extracts	Minimum bactericidal concentration (MBC) mg/mL*			
	*MRSA USA300*	*Acinetobacter baumannii AB5075*	*Escherichia coli ATCC87*	*Pseudomonas aeruginosa PAO1*
Onion	3.125 ± 0	4.167 ± 1.8	12.5 ± 0	10.417 ± 3.6
Garlic	12.5 ± 0	6.25 ± 0	12.5 ± 0	12.5 ± 0
Potato	–	–	12.5 ± 0	6.25 ± 0

*Data are presented as mean ± standard deviation.

– No antibacterial activity was detected against the tested organism.

### Wound-healing effects of onion peel extracts

The promising antibacterial and antiiflammoatory activities encouraged us to evaluate the wound healing effects of onion peel extracts in vitro and in vivo using conventional and nano-formulations.

#### Preparation of LNC_OPE_ and in-vitro characterization of the prepared LNC_OPE_

Herein, LNC_OPE_ is fabricated by adopting phase inversion technique. This method is simple and omits high energy consumption and organic solvents^67^. The phase inversion technique consists of two consequent steps. First, oil and surfactants are mixed with sodium chloride to obtain the primary emulsion. In the second step, heating the blend above the phase inversion temperature (70 °C) results in the formation of water in oil emulsion that is inverted to oil in water emulsion upon cooling below the phase inversion temperature (40 °C). The addition of cold water at the third cycle triggers irreversible shock that breaks the emulsion system and favors the solidification of the lipid to form a shell thus forming stable nanocapsule^16^.

Table 3 demonstrates the in-vitro physicochemical characteristics of the prepared LNC_OPE._ Generally, particle size is one of the important factors affecting different nanocarriers’ therapeutic potential^44^. LNC_OPE_ had a particle size of 38.54 nm ± 4.88. This relatively small particle size could be attributed to the presence of Kolliphor® HS15 and Epikuron 200®. These surfactants could diminish interfacial oil tension and, consequently the globule size^41^. The measured PDI value was 0.19, indicating the formulation of a homogenous monodisperse system^42^. High onion peel extract EE% (87.99% ± 3.57) could be attributed to surfactants that could improve the extract solubility in the oil core. The appearance of LNC_OPE_ as a spherical non-aggregated particle with a particle size 30–40 nm is agreed with the DLS determinations (Fig. S16).

**Table 3:** Characterization of the prepared LNC_OPE_ formulation.

LNC_OPE_	Particle size (nm)^a,d^	PDI^a,d^	Zeta potential (mV)^b,d^	EE %^c,d^
Characterization	38.54 ± 4.88	0.19 ± 0.02	− 13.87 ± 1.57	87.99 ± 3.57

^a^Measured by DLS.

^b^Measured by electrophoresis.

^c^Calculated as a percentage of initial onion peel extract added, determined directly by HPLC.

^d^Expressed as mean ± SD (n = 3).

#### In-vitro evaluation of LNC_OPE_ based hydrogel

**Figure 3:**
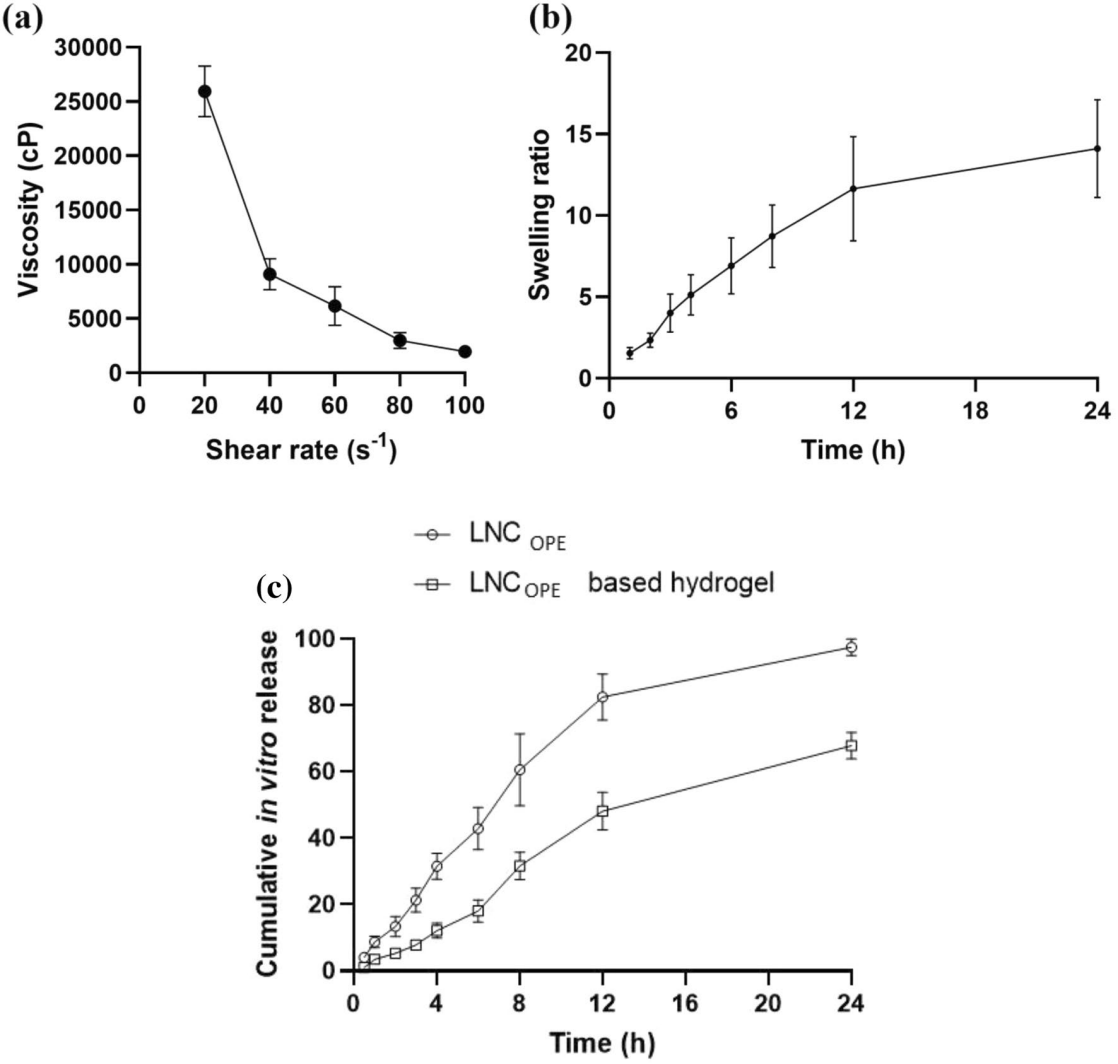
In-vitro characterization and drug release of the prepared LNC_OPE_ based hydrogel. (**a**) viscosity, (**b**) swelling rate and (**c**) in-vitro drug release in phosphate buffer (pH 5.5). Data are expressed as the mean ± SD.

The prepared topical hydrogel was translucent, homogenous, and non-gritty, with a pH value of 5.68 ± 0.68. The slightly acidic nature of the proposed hydrogel could be beneficial in topical delivery to avoid irritation as it mimics the skin pH^68^. The viscosity of the prepared LNC_OPE_ based hydrogel as a function of shear rate is expressed in Fig. 3a. It could be noticed that system viscosity is inversely proportional to the shear rate indicating a pseudoplastic flow. In topical preparations, pseudoplastic flow is favored over other types where it could be easily spread upon medium shear force^45^.

The spreadability of LNC_OPE_ was assessed using the glass slide method, where the value was found 345 mm^2^ ± 1.1. The spreadability is an indicator of the ease of application and consistency of hydrogels. This result is in accordance with previous reports that indicate this value would be accepted for ease of spreading and application^17,46^.

To assess the capacity of LNC_OPE_ based hydrogel to provide wet environment when they are utilized onto an open wound area, the swelling behavior in PBS pH 5.5 was investigated. The hydrogel’s high absorption capacity, which is important for rapid absorption of exudates to keep the wound dry and precludes airborne infection^69^. It could be depicted from Fig. 3b that the swelling ratio of LNC_OPE_ increases with time. The swelling ability of carbopol 940 could be its carboxylic acid moieties^70,71^.

The in vitro release profile of OPE from LNC and LNC-based hydrogel is displayed in Fig. 3c. The acidic media was used to mimic the pH of the skin. LNC showed a higher release rate than LNC-based hydrogel at all time points. LNC showed burst release where about 21.29% ± 3.57 of OPE was released after 3 h. An almost 100% OPE from LNC was observed 24 h. On the contrary, only 67.84% ± 3.26 OPE was detected from LNC-based hydrogel at 24 h with no burst release. This could be attributed to the ability of carbopol to act as a barrier and increase the viscosity that hinders drug release^46^.

#### Effect of OPE on Hfb4 cell cytotoxicity, cell viability, inflammatory markers and oxidative stress biomarkers

Treatment of Hfb4 cells with the formulations and their blanks revealed a reverse relationship between concentration and cytotoxicity (Fig. S17). The highest cytotoxicity was observed at a dose of 0.093 mg/mL (9.23, 8.54, 3.76, and 3.28%; respectively for blank 1, blank 2, free OPE, and LNC_OPE_), whereas the lowest cytotoxicity was observed at a dose of 3 mg/mL (7.65, 6.45, 3.11, and 2.54%; respectively for blank 1, blank 2, free OPE, and LNC_OPE_). These results indicate that the onion-containing formulations were non-toxic to the cells compared to the extract-free blanks.

Regarding cell viability, it was a concentration-dependent; the highest cell viability was observed at 3 mg/mL (Fig. S17). At all used concentrations, free OPE showed a significant increase in the viability of the cells compared to its blank by 15.64, 16.74, 16.24, 20.37, 20.12, and 21.9%, *P* < 0.01, respectively at 3, 1.5, 0.75, 0.37, 0.18, and 0.09 mg/mL. In the same manner, the LNC_OPE_-treated cells showed significant elevation in the viability of the cells by 17.08, 18.34, 20.08, 22.4, 22.18, and 23.23%, *P* < 0.01, respectively at the same serial dilution compared to blank 2. Furthermore, when the cells were treated with LNC_OPE_, it showed a significant increase in their viability compared to free OPE at all used concentrations by 2.56, *P* = 0.004; 2.19,* P* = 0.029; 3.99, *P* = 0.001; 2.6, *P* = 0.007; 3.21, *P* = 0.001; and 2.61%, *P* = 0.001, respectively.

**Figure 4:**
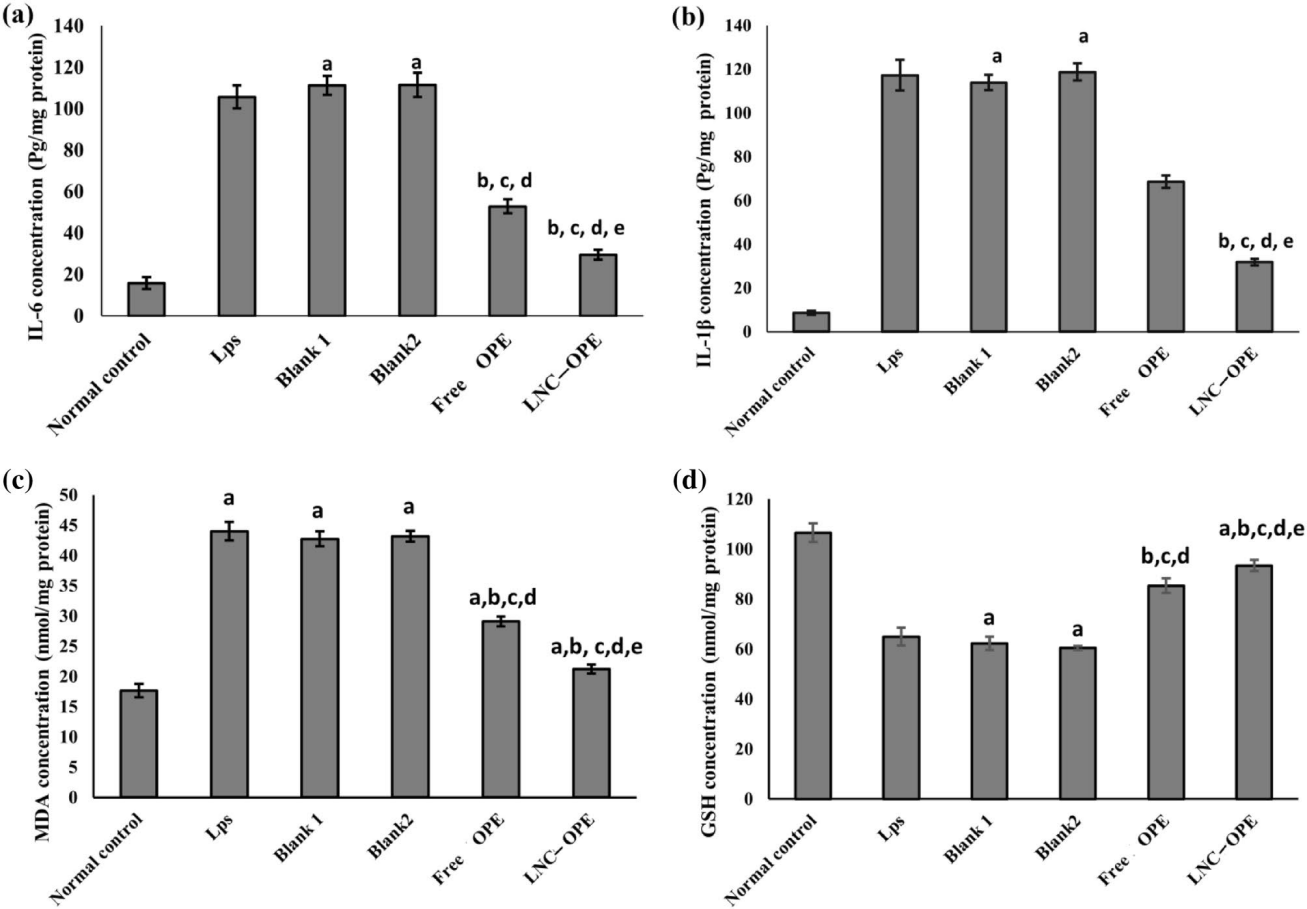
Effect of free OPE and LNC_OPE_ and their blanks on inflammatory markers and oxidative stress biomarkers. (**a**) IL-6, (**b**) IL-1β, (**c**) MDA and (**d**) GSH. Data are expressed as the mean ± SD and were analyzed using one-way ANOVA followed by Tukey post hoc test. Values were considered significantly different at *p* < 0.001. a, significant *versus* normal control; b, significant *versus* LPS only; c, significant *versus* blank 1; d, significant *versus* blank 2 and e, significant *versus* free OPE. GSH, glutathione; IL-6, interleukin 6; IL-1β, interleukin 1 beta and MDA, malondialdehyde.

LPS only-treated cells showed a significant increase in IL-6 and IL-1β by 5.68 and 12.63 folds, *P* < 0.001; respectively, compared to normal control cells. Treatment of the cells with free OPE and LNC_OPE_ significantly decreased the concentration of these cytokines by 50.04 and 41.52%, respectively, *P* < 0.001 for free OPE, whereas by 72.13 and 72.86%; respectively, *P* < 0.001 for LNC_OPE_ in comparison with the LPS only group. When comparing the formulations with their corresponding blanks, the OPE showed a significant reduction in IL-6 and IL-1β (52.56 and 39.79%; respectively, *P* < 0.001), and the same was observed for LNC_OPE_ (73.56 and 73.18%; respectively, *P* < 0.001). It is worth mentioning that the LNC_OPE_ formulation showed a significant decrease in the concentration of the cytokines by 44.21 and 53.59%, respectively, *P* < 0.001 relative to the free OPE group (Fig. 4).

As shown in Fig. 4, the LPS control group showed significant elevation in the level of MDA (1.49 fold, *P* < 0.001) with a substantial decrease in the concentration of GSH (39.02, *P* < 0.001) compared to the normal control group. Treatment of Hfb4 cells with free OPE and LNC_OPE_ significantly decreased the concentration of MDA (33.9 and 51.8%, *P* < 0.001; respectively) and increased the concentration of GSH (31.40 and 43.71%, *P* < 0.001; respectively) in comparison with the LPS only group. When comparing the formulations with their corresponding blanks, both free OPE and LNC_OPE_ significantly decreased the concentration of MDA (31.93, 50.86%, *P* < 0.001; respectively) and increased the level of GSH (36.99, 54.54%, *P* < 0.001; respectively). In addition, treatment with LNC_OPE_ significantly decreased the MDA content of Hfb4 (27.08%, *P* < 0.001) and increased their content of GSH (9.37%, *P* = 0.005) relative to the free OPE-treated cells.

#### Effect of OPE on excision wound model

The rate of wound contraction in the group treated with the free extract was significantly higher than the untreated group, and it was 0.186–40.24% from day 1 to day 12 compared to 0.16–7.11% for the untreated group (Table 4). At the end of the experiment, the free OPE and LNC_OPE_ showed an increased rate of wound contraction by 4.59 and 7.61 folds, respectively, compared to their corresponding blanks. Treatment of the mice with LNC_OPE_ significantly enhanced the rate of wound contraction (0.29–63.99%) compared to the free extract. It is worth mentioning that the rate of wound contraction was in the following descendant order: LNC_OPE_ > free OPE > blank 2 > blank 1 > untreated, as presented in Fig. S18.

**Table 4:** Rate of wound contraction.

Wound contraction (%)	Untreated	Blank 1	Blank 2	Free OPE	LNC_OPE_
Day 1	0.16 ± 0.37	0.06 ± 0.08	0.36 ± 0.46	0.186 ± 0.21	0.29 ± 0.40
Day3	0.49 ± 0.22	0.56 ± 0.27	0.66 ± 0.38	7.7 ± 2.10^a b c^	17.04 ± 2.59^a b c d^
Day 6	1.71 ± 0.4	1.73 ± 0.35	2.03 ± 0.31	17.8 ± 2.07^a b c^	34.36 ± 4.55^a b c d^
Day 9	3.7 ± 2.57	3.79 ± 2.56	4.06 ± 2.52	30.2 ± 2.3^a b c^	49.53 ± 4.04^a b c d^
Day 12	7.11 ± 3.12	7.19 ± 3.12	7.43 ± 3.11	40.24 ± 5.64^a b c^	63.99 ± 6.4^a b c d^

a, significant *versus* untreated group; b, significant against blank 1; c, significant *versus* blank 2; d, significant *versus* free OPE at *p* < 0.001.

**Figure 5:**
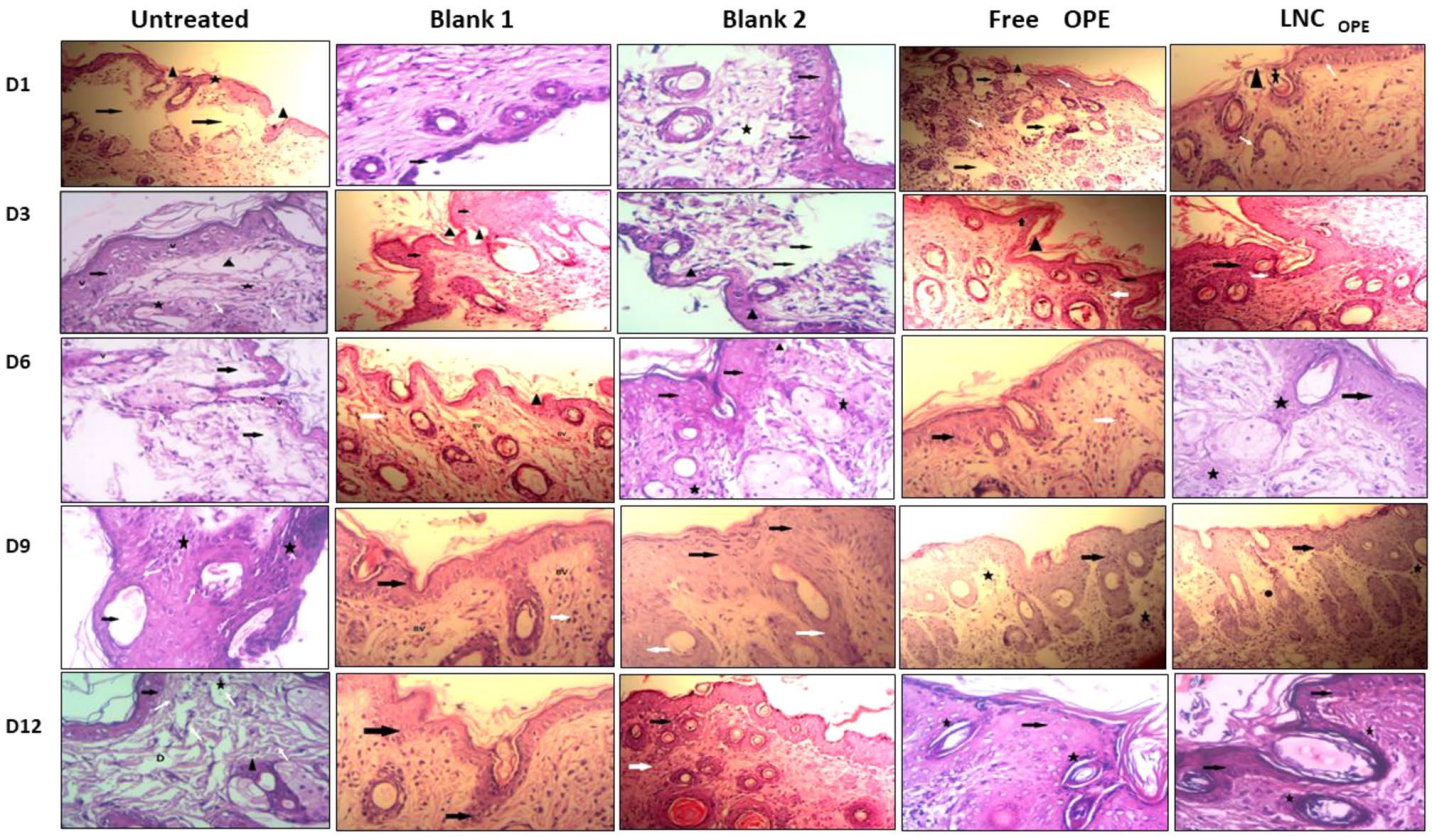
Effect of free OPE and LNC_OPE_ and their blanks on histopathology of the wound tissue stained by H&E and visualized at 200×.

Figure 5 shows that on the first day of wounding, the untreated group exhibited ulceration (arrow head), edema (star), and lost skin tissue with the disturbed architecture of the layers of skin (arrow). On days 3, 6, and 9, the untreated mice started to show epidermal cells (arrow) with some vacuolation (V), congested blood vessels (star on day 3), and lost tissue (arrow head). The inflammatory phase startedon day 3 and continued on day 6. There was an excessive accumulation of granulation tissue (star) at day 9; indicating the proliferative healing phase. On the twelfth day, there was a thin epidermis (arrow), dilated blood vessels (star), and an accumulation of granulation tissue (arrow head).

Regarding blank 1-treated groups, it showed ulceration with the disturbed architecture of the layers of the skin (arrow) on the first day. There was a disturbed architecture with edema (arrow head), and new epithelialization started appearing (arrow) on the third day. At day 6, there was a slightly healed epidermis (arrow head), minimal granulation tissue (white arrow), and good vascularity (BV). Relative epithelization (dark arrow), granulation tissue (white arrow), and blood vessels were observed on day 9. In the end, good epithelization (dark arrow) was detected.

Blank 2-treated mice exhibited ulceration with the disturbed architecture of the layers of the skin (arrow) on the first day of the treatment. On day 3, the mice slightly showed healed epidermis (arrow head), but there were some lost areas (arrow). Later on day 6, there was a slightly healed epidermis (arrow) with little accumulation of granulation tissue (star) and congested blood vessels (arrow head). Good epithelization (dark arrow) and granulation tissue (white arrow) were detected at day 9. Finally, on day 12, good epithelialization (dark arrow head) and severe granulation tissue (white arrow) were observed.

Concerning free OPE, the mice showed ulceration (arrow head), edema, and lost skin tissue with disturbed. The treatment on the third day showed edema (arrow head), and new epithelialization started appearing (dark arrow). The sixth day was similar to the third, but good granulation tissue was observed (white arrow). At day 9, good epithelialization (dark arrow head), and severe granulation tissue (white arrow). On the last day, there was a normal structure of the epidermis (arrow) and hair follicle (star), the architecture of the layers of the skin (arrow), and monocellular infiltrate (white arrow) on the first day. This indicates the early inflammatory and proliferative stages compared to the untreated control and the corresponding blank 1.

The LNC_OPE_-treated mice started with ulceration (arrow head), monocellular infiltrate (white arrow), edema, and destructed hair follicles (star). Day 3 showed a new epithelialization (dark arrow) and increased vascularity (BV). The mice showed good architecture of the epidermis (arrow) with the accumulation of granulation tissue (star) at day 6. Normal skin architecture formed of epidermis and dermis with good epithelialization (dark arrow), severe accumulation of granulation tissue (white arrow), and deposition of collagen fibers (circle) was observed at day 9. At the end of the treatment, there was a normal structure of the epidermis (arrow) and the hair follicle (star). These results signify an accelerated wound healing process compared to the untreated and blank 2 groups. In addition, the inflammatory cells, the granulation tissue, and the collagen fibers were more dense than free OPE.

Concerning the effect on collagen precipitation, MT staining shows that the untreated group exhibited mild collagen deposition from day 1 to day 6 (Fig. S19). The deposition was increased gradually from day 9 to reach the maximum at day 12. Similarly, blank 1 and 2-treated groups exhibited mild collagen precipitation from day 1 to day 6 with2-treated groups exhibiting mild collagen precipitation from day 1 to day 6 with an abrupt increase at day 9 to reach the highest level at day 12. Both free OPE and LNC_OPE_-treated groups started with mild collagen deposition with severe precipitation on day 9 for free OPE and on day 6 for LNC_OPE_.

#### Effect of OPE on TNF-α and α-SMA immunoreactivity

**Figure 6:**
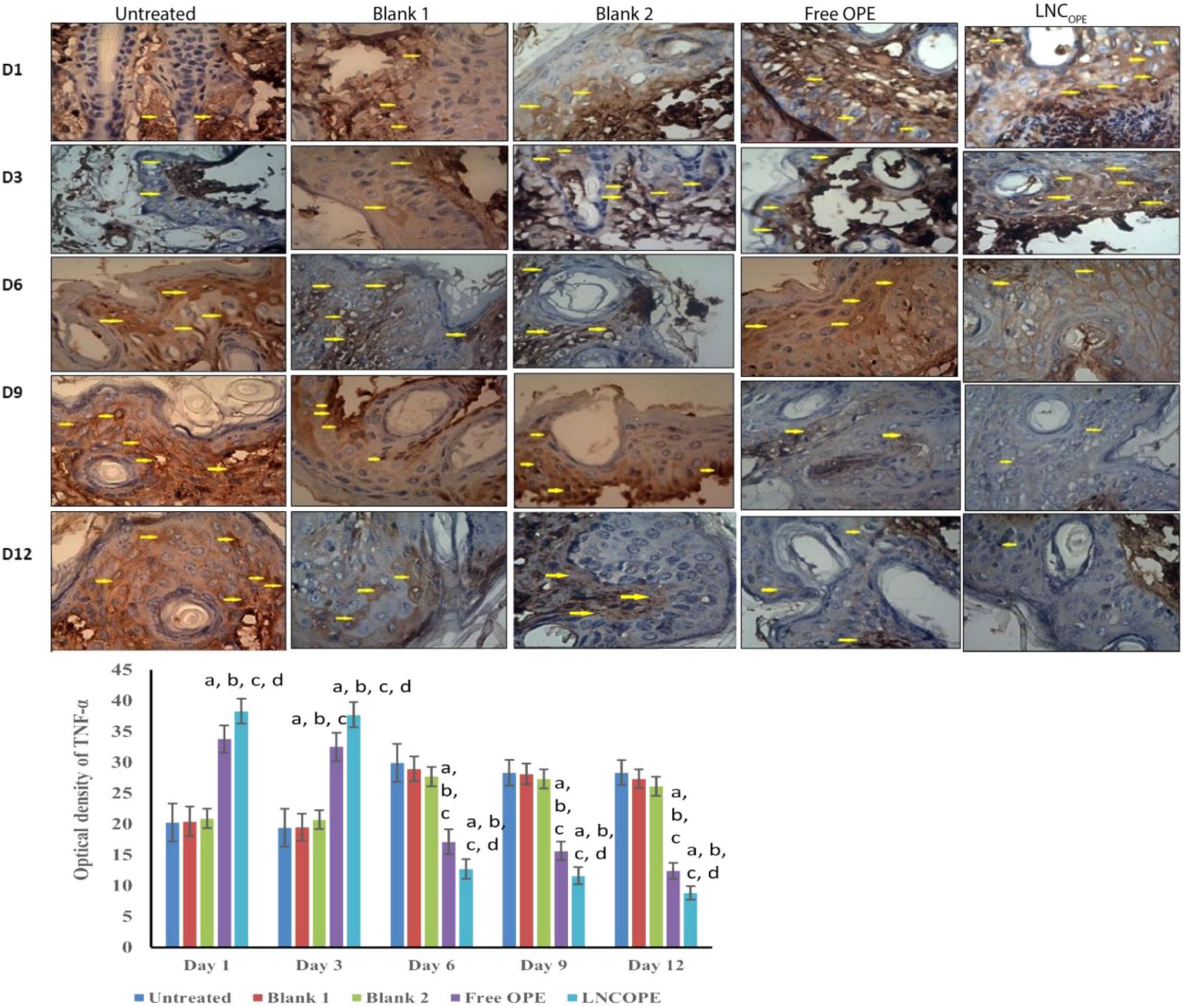
Effect of free OPE and LNC_OPE_ and their blanks on TNF-α immunohistochemistry and the quantified optical density. Data are expressed as the mean ± SD and were analyzed using one-way ANOVA followed by Tukey post hoc test. Values were considered significantly different at *p* < 0.05. a, significant *versus* normal control; b, significant *versus* blank 1; c, significant *versus* blank 2 and d, significant *versus* free OPE. TNF-α, tumor necrosis factor-alpha.

The effects of free OPE and LNC_OPE_ and their blanks on TNF-α and α-SMA immunoreactivity were evaluated (Fig. 6). The untreated group started with mild immunoreactivity and gradually increased to severe at day 6 and beyond. The blank 1 group showed mild reactivity on day 1, severe at day 6, and decreased to moderate at day 12. Blank 2 group started with moderate reactivity, reached severity at day 6 and decreased again to mild at day 12. Free OPE and LNC_OPE_-treated mice showed high immunoreactivity toward TNF-α at the first day, but LNC_OPE_ was more severe. Afterward, both groups showed a gradual decrease in the reactivity to reach very mild immunoreactivity at day 12. Statistical measures reflected that free OPE and LNC_OPE_ groups showed a significant increase in the OD of TNF-α in the first day by 67.32% and 89.6%, *P* < 0.001; respectively, compared to the untreated group. The free OPE group showed a significant increase in the OD by 65.68% and by 66.66%, *P* < 0.001; respectively, on day 1 and day 3 in comparison with its blank. In contrast, there was a significant decrease in the OD of free OPE compared to blank 1 at day 6, 9, and 12 (40.83, 44.48, and 54.57%,* P* < 0.001; respectively). Treatment of the mice with LNC_OPE_ significantly elevated the OD of TNF-α by 83.25 and by 82.12%, *P* < 0.05; respectively, at day 1 and day 3 relatively to blank 2. At day 6, 9, and 12 there was a significant decrease in the OD for LNC_OPE_ group compared to its blank (54.15, 57.5, and 66.28%; *P* < 0.001). Ultimately, there was a statistically significant difference between the free OPE and LNCOPE-treated groups where LNCOPE decreased the OD of TNF-α by 29.03%, P = 0.007 relative to free OPE. These results are consistent with H & E histopathological investigation; both indicate earlier inflammatory phase in the formulations-treated groups compared to the untreated group and their blanks.

Regarding α-SMA, it was assessed to indicate myofibroblast activity as presented in Fig. S20. In the first day, all the studied groups showed mild immunoreactivity toward α-SMA except LNC_OPE_-treated group; it showed an increased reactivity. The reactivity of the untreated group was increased gradually from day 6 to day 12. For blank 1 and 2 groups, immunoreactivity for α-SMA was increased at day 6 with severe reactivity at day 9 and day 12. Free OPE and LNC_OPE_-treated groups exhibited severe reactivity against α-SMA starting from day 6 but LNC_OPE_ group was more highly reactive than free OPE. These observations were consistence with the statistical analysis; only LNC_OPE_-treated mice showed significant increases in the OD of α-SMA by 29.82%, *P* = 0.003 in the first day compared to the untreated group. On the other hand, free OPE group revealed a significant elevation in the OD against blank 1 starting from day 6 (21.14, *P* < 0.001; 16.59,* P* < 0.001; and 26.3%,* P* < 0.001; respectively at day 6, 9, and 12). Likewise, the LNC_OPE_-treated mice showed significant increase in the OD of α-SMA compared to blank 2 (25.56, 30.85, and 36.11%,* P* < 0.001; respectively, at day 6, 9, and 12). At the end, the LNC_OPE_ group showed significant increase in the OD of α-SMA by 11.9%, *P* = 0.002 compared to free OPE group. These findings are in harmony with H & E staining, which indicated early proliferative phase in free OPE and LNC_OPE_-treated groups but LNC_OPE_ showed more pronounced proliferative features.

#### Effect of OPE on genes’ expression

**Figure 7:**
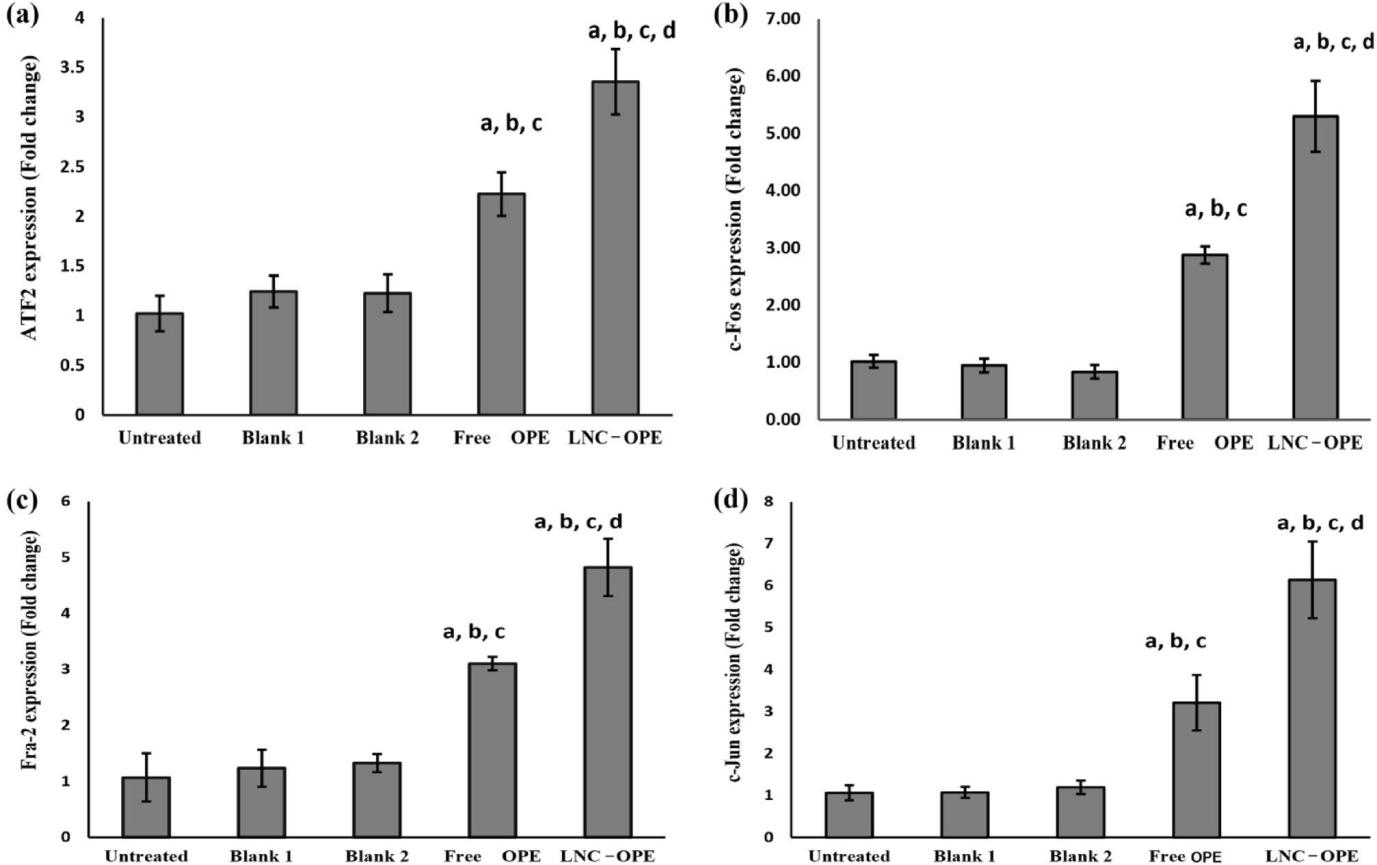
Effect of free OPE and LNC_OPE_ and their blanks on the expression level of AP-1 signalling-related proteins. (**a**) ATF-2, (**b**) c-Fos, (**c**) Fra-2, and (**d**) c-Jun. Data are expressed as the mean ± SD and were analyzed using one-way ANOVA followed by Tukey post hoc test. Values were considered significantly different at *p* < 0.05. a, significant *versus* normal control; b, significant *versus* blank 1; c, significant *versus* blank 2 and d, significant *versus* free OPE. ATF-2, activating transcription factor 2; c-Fos, Fos proto-oncogene; Fra-2, Fos-related antigen 2; c-Jun, Jun proto-oncogene.

Free OPE and LNC_OPE_-treated mice showed significant increase in the expression level of ATF-2, c-Fos, Fra-2, and c-Jun by 1.17, *P* = 0.012; 1.82,* P* = 0.001; 1.89, *P* = 0.005 and 2, *P* = 0.001 folds; respectively, for the free OPE group and by 2.28, 4.2, 3.5, and 4.75, *P* < 0.001; respectively, for the LNC_OPE_ group comparing with the untreated group (Fig. 7). Free OPE group exhibited significant up-regulation of these genes (0.78, *P* = 0.033; 2.03,* P* = 0.001; 1.51, *P* = 0.008 and 1.99, *P* = 0.001 folds; respectively) relatively to its corresponding blank 1-treated group. When comparing LNC_OPE_ group to blank 2, it showed significant increase in the expression level of the genes by 1.73, 5.33, 2.63, and 4.13 folds, *P* < 0.001; respectively. Comparing LNC_OPE_ with free OPE showed that LNC_OPE_ significantly upregulated the expression of the genes by 50.76,* P* = 0.017; 84.19, *P* < 0.001; 55.38, *P* = 0.012; and 91.17%,* P* < 0.001; respectively.

### Molecular docking between onion peel bioactive metabolites on p65/Atf-2

Analyzing the binding modes of the most promising investigated compounds within the binding pocket of p65 (Table 5), it was found that quercetin-3,4′-*O*-diglucoside (S = − 7.182 kcal/mol and RMSD = 1.565) formed six H-bonds with Lys93 (2 H-bonds), Asp103, Ser112, Ile110, and Gln114 at 2.92, 3.17, 2.97, 2.97, 3.13, and 3.14 Å, respectively. Also, it formed an extra pi-H interaction with Ser112 at 4.04 Å. Additionally, alliospiroside C (S = − 7.024 kcal/mol and RMSD = 2.161) showed the formation of four H-bonds with Ser112, His111, and Lys93 (2 H-bonds) at 2.83, 2.85, 2.89, and 3.07 Å, respectively. Moreover, alliospiroside D (S = − 7.282 kcal/mol and RMSD = 2.212) formed three H-bonds with His111, Asp94, and Lys93 at 3.07, 3.14, and 3.20 Å, respectively. It formed an H-pi interaction with Phe113 at 4.06 Å as well.

**Table 5:** Binding scores, RMSD, 3D binding interactions, and 3D positioning of the most promising compounds from the selected vegetable byproducts (quercetin-3,4′-*O*-diglucoside, alliospiroside C, and alliospiroside D) inside the binding pockets of the p65 and ATF-2 target receptors.

Compound	R	S	RMSD	2D interaction	3D interaction	3D positioning
Quercetin-3,4′-*O*-diglucoside	p65	− 7.182	1.565			
	ATF-2	− 9.205	1.356			
Alliospiroside C	p65	− 7.024	2.161			
	ATF-2	− 7.344	1.567			
Alliospiroside D	p65	− 7.282	2.212			
	ATF-2	− 8.794	1.918			

R, receptor; S, score of a compound within the receptor binding pocket (Kcal/mol).

On the other hand, observing the binding modes of the most promising investigated compounds within the binding pocket of ATF-2, it was concluded that quercetin-3,4′-*O*-diglucoside (S = − 9.205 kcal/mol and RMSD = 1.356) formed three H-bonds with Asn156, Arg69, and Asn114 at 2.05, 2.95, and 3.25 Å, respectively. Moreover, it formed two extra H-bonds with Asn156 through an Mg^2+^ ion bridge at 2.24 and 3.30 Å, respectively, besides, a pi-H interaction with Val40 at 4.56 Å. Also, alliospiroside C (S = − 7.344 kcal/mol and RMSD = 1.567) was found to be able to form two H-bonds with Ser155 and Asn114 at 2.72 and 3.22 Å, respectively. At the same time, it bound Asn156 with two H-bonds through an Mg^2+^ ion bridge at 2.10 and 2.14 Å, respectively. Alliospiroside D (S = − 8.794 kcal/mol and RMSD = 1.918) achieved the formation of six H-bonds with Thr188, Ser155 (2 H-bonds), and Arg69 (3 H-bonds) at 2.67, 2.73, 2.83, 2.87, 2.92, and 2.99, respectively. Besides, it formed two extra H-bonds with Asn156 through an Mg^2+^ ion bridge at 2.07 and 2.14 Å, respectively.

## Discussion

The high volume of vegetable biological wastes generated by culinary uses and industrial processes raises the cost of environmental protection. Consequently, new strategies for recycling biological waste are indispensable to transform them into sustainable value-added products. Thus, the potential of this waste for various applications such as bioenergy, feed alimentation, and pharmaceutical production has been reported^72,73^. Valorization of vegetables wastes and their by-products (e.g. potato, onion and garlic peels) can meet the demands for potential pharmaceuticals and cosmeceuticals applications at the industrial levels^74–76^. Potato, onion, and garlic peels are renewable resources for bioactive metabolites. However, the comparative study on biological activities as an anti-inflammatory in the context of osteoarthritis and antimicrobial in the context of wound healing remains unknown.

Osteoarthritis is a common orthopedic degenerative joint disease representing the most common chronic musculoskeletal disorder that drives cartilage extracellular matrix degradation, affecting millions of individuals worldwide^77^. Metabolic diseases, age, sex, trauma and hormonal disturbance are among the risk factors related to the devolvement of osteoarthritis^78^. Osteoarthritis can affect all joints, particularly the knee, spine, shoulder, hip, feet, and hands, leading to chronic pain, cartilage stiffness, inflammation, and joint disability in older adults^78^. Pharmacological therapies as non-steroidal anti-inflammatory drugs (NSAIDs), acetaminophen, duloxetine, hyaluronic acid, glucosamine, and chondroitin are commonly prescribed to relieve symptoms of osteoarthritis^79,80^. Thus, it remains a challenge to develop an efficient, safe, cost-effective, and sustainable pharmacological treatment for the management of osteoarthritis from plant resources^81^. Traditionally, onion peels, as well as their boiled aqueous extract, have been used to relieve symptoms of muscle cramping pain in the legs and inflammation in the knees. However, the scientific evidence for such uses remains to be investigated. The potential effects and mechanism of onion peel extract on IL-1β-stimulated chondrocytes are unclear. Therefore, in the present study, we assessed the ability of the tested peel extracts to protect against inflammation and collagen breakdown in response to IL-1β treatment in mouse-isolated chondrocytes. Onion peel extract significantly inhibited the overproduction of NO, MMP-13, NF-κB p65, IL-6, TNF-α, COX-2 and iNOS induced by IL-1β. These findings suggested that onion peel extract showed the best anti-inflammatory activity. Furthermore, our analysis revealed that, in contrast to the IL-1β induction, treatment with onion peel extract inhibited IL-1β-stimulated MMP-13 production and collagen II degradation, and thus protecting chondrocytes from IL-1β-stimulated extracellular matrix (ECM) degradation. Due to its ability to stimulate the transcription of inflammatory mediators, the NF-κB pathway plays a significant role in inflammation^82,83^. Our results also showed that onion peel extract showed significantly inhibited IL-1β-stimulated NF-κB p65 production, even better than celecoxib. However, further research is needed to establish whether onion peel extract or its bioactive metabolites exerted in vivo anti-inflammatory effects via the NF-κB pathway (Fig. S21).

Further, we also evaluated the potential antibacterial activities of peel extracts. against highly virulent bacterial strains belonging to ESKAPE pathogens (the world’s leading cause of nosocomial infections)^84^. Previous studies reported the antibacterial activity of onion (*Allium cepa*) and garlic (*Allium sativum*), but little was reported about potato (*Solanum tuberosum*)^85^.

The antibacterial activity of the onion peels extract was superior to that of garlic and potato peels extracts in our study and that of other botanical extracts in previous studies^39,86–88^. The superiority of the antibacterial activity of the onion peels extract was demonstrated by lower minimum bactericidal activity and broad antibacterial spectrum activity against both Gram negative and Gram positive highly resistant bacterial strains.

The antibacterial activity from our screening revealed that onion peel extract exhibited promising activity against MRSA. We then extended these studies into a mouse model to explore the wound-healing potential of onion peel extracts. Many plants contain biologically active compounds, which were recently captured much more attention in the biomedical fields^89,90^. Onion peels are major source for various phytochemical compounds that possess potent anti-oxidant and anti-inflammatory properties^91^. In this context, we also aimed to investigate its activity against LPS-induced inflammation in Hfb4 normal skin cells and further against in-vivo wound healing model. In addition, we compared the activity between the conventional free extract hydrogel and its nano-capsulated form in-vitro and in-vivo. Lipid nanocapsule (LNC) is a biomimetic lipid nanocarrier composed of a mixture of oil core coated with a shellconsisting of solid lipids and emulsifier^15^. In this study, the oil core is composed of labrafac that is medium-chain triglycerides with previously reported biocompatibility, penetration enhancement potential, and provides LNC bulkiness^92,93^. Kolliphor® HS 15 is a non-ionic surfactant composed of a mixture of PEG 660 and PEG 660 hydroxystearate that improve LNC stability and stealth features^67,94^.

Moreover, the lipid surfactant Epikuron is phosphatidylcholine improves the LNC biocompatibility and shell rigidity^67^. Finally, sodium chloride is usually added during the fabrication of LNC to modulate the phase inversion temperature to suitable range^94^. The major drawback of most nanoparticles is the burst payload release that could compromise therapeutic efficiency and clinical application^17^. The incorporation of nanocarriers in other carrier as hydrogels or scaffolds could overcome this challenge^18^. Hydrogel is a semisolid preparation that showed superior potential in different biomedical applications as tissue regeneration, and sustained and local drug release^19^. In addition, hydrogel is a 3-D porous matrix with high water content and swelling degree^20^. Interestingly, the incorporation of lipid nanocarriers into the hydrogel is supposed to improve the mechanical properties of the hydrogel as well as prolong drug release^21^.

To ensure the safety of the used formulations, the cytotoxicity and cell viability of the free OPE and LNC_OPE_ were examined against Hfb4 cells and revealed no cytotoxic effect with enhanced cellular viability compared to their extract-free blanks. Previous reports preceded us in reporting the non-cytotoxic effect of OPE against cultured fish liver cells^95^.

LPS is commonly used to induce inflammation and oxidative damage in various in-vitro and in-vivo models^49^. In our in-vitro experiments, the LPS control group showed elevated levels of IL-6, IL-1β, and MDA and low levels of GSH. IL-6 and IL-1β are common inflammatory cytokines, whereas MDA is a predictor of lipid peroxidation and the GSH is a potent free radical scavenger and highly contributes to the anti-oxidant capacity of the cells^96^. The LPS can induce reactive oxygen species (ROS) and nitric oxide (NO); thereby, generating oxidative stress microenvironment. It can also stimulate the release of different inflammatory mediators as a result of the activated microphages, which also generate NO. Therefore, the inflammatory response and oxidative stress can be intercalated in the pathogenesis induced by LPS^97^. Tang et al.^98^ suggested that LPS can also induce inflammation and ROS through the activation of the mitogen-activated protein kinase (MAPK) signaling pathway and nuclear factor-kappa B (NF-κB). Our results are in line with others that demonstrated that the LPS generates an inflammatory response and oxidative stress^49,98–100^.

Treatment of the cells with free OPE and LNC_OPE_ reversed the detected parameters in the LPS only group. This favorable effect could be attributed to the presence of polyphenolic compounds, which are valuable natural antioxidants and significant determinants of most natural extracts' antioxidant capacity^96^. More specifically, quercetin is one of the major flavonols in the onion and, interestingly, is present in high amounts in the outer dry scales compared to the inner pulp. Flavonoids can inhibit cyclooxygenase enzymes that are responsible for the production of prostaglandins, which play a crucial role in initiating inflammation^101^. Furthermore, quercetin can downregulate the pro-inflammatory cytokines. Onion peel extract was also found to enrich the antioxidant defense enzymes; superoxide dismutase (SOD) and catalase (CAT)^102^. Our findings are in harmony with previous studies that reported the anti-inflammatory and anti-oxidant activity of onion peel extracts^102–105^.

Concerning the wound in-vivo model, it is well known that the skin represents a substantial part of the body's defense mechanism. Accordingly, skin injuries disrupt the anatomical structure and the physiological functions of the skin and even may make it vulnerable to infections if not promptly and adequately healed. Therefore, repairing the injured skin is essential to restore its functions^50^. The wound healing process has four distinct stages; hemostasis, inflammation, proliferation, and remodeling/maturation. The hemostasis starts with vasoconstriction to limit the blood flow and is rapidly followed by the initiation of blood clotting cascade. In the inflammatory step, the neutrophiles and macrophages are recruited to the site of injury and the macrophage produce different pro-inflammatory cytokines and chemokines including IL-1, IL-6, IL-27, and TNF-α^106^. Keratinocytes and fibroblasts mainly mediate the proliferation phase. Re-epithelialization and vascularization also take place in this stage. Precisely, fibroblasts form a new extracellular matrix (ECM) in which collagen III is predominant, leading to the formation of the granulation tissue and, ultimately, the scar. Then, the fibroblasts become myofibroblasts that express α-SMA, which contracts the borders of the wound^107^. Finally, collagen III is replaced by more strength and mature collagen I in the maturation phase. It is noteworthy that any disruption in these phases can lead to impaired wound healing and diseases or infections; thereby, an accelerated healing process is crucial^108^.

The untreated mice showed a normal pattern of the typical wound healing process and its characteristic features within the usual time required for a wound to heal ≈ 12 days. After treatment of the mice with onion peel extract hydrogels, the healing was accelerated compared to the untreated mice. The inflammatory phase was observed earlier at day 1 with mononuclear infiltration. It increased TNF-α, as indicated by the histopathological and immunohistochemical investigations, compared to the untreated group in which the inflammation was started at day 3. Concerning the proliferative phase, MT stain was used to provide clear morphological features required for the assessment of wound healing that could not be offered by H & E staining including collagen deposition, which stained bright blue color^109^. In the untreated group, considerable amount of collagen deposition was detected at day 9, which helped the formation of the granulation tissue, and simultaneously, the myofibroblasts expressed the α-SMA. These steps were earlier in the extracts-treated groups (at day 6) and the LNC_OPE_-treated group showed more dense collagen precipitation and more α-SMA compared to free OPE. These histopathological and immunohistochemistry analyses align with the wound contraction rate since the nano-formulation showed the highest wound contraction followed by the free extract hydrogel.

On the other hand, activating protein-1 (AP-1) signaling pathway is involved in multiple cellular processes including, but not limited to, inflammatory response, cellular growth, differentiation, and apoptotic cascade. AP-1 consists of four basic protein family; Jun, Fos, ATF, and musculoaponeurotic fibrosarcoma (Maf)^110^. AP-1 and its related proteins are expressed in different manner throughout the layers of the skin and it was found that they are involved in several skin-related processes such as keratinocytes differentiation, response to ultraviolet radiation, photo-aging, and even repairing of the skin injuries^111^. The underlying mechanism for the accelerated wound repair in onion peel extracts-treated groups could be the modulation of AP-1 signaling and further the inflammatory cytokines. The untreated group showed downregulated AP-1, which indicated by its components of ATF-2, c-Fos, Fra-2, and c-Jun. Treatment of the mice with the extracts has upregulated these genes, which could enhance the wound healing inflammatory phase. Our results are in harmony with Ha et al.^112^ who demonstrated the beneficial role of AP-1 on human keratinocytes and with Lee et al.^113^ who reported that AP-1 signaling stimulates myofibroblast differentiation and production of ECM.

It is worth to mention that the nano-formulated hydrogel showed superior results relatively to the free extract and this could be ascribed to the advantages of LNC involving better biocompatibility, improved biodegradability, small particle size (< 100 nm) that ease its entrance to the cells, physical stability, and high encapsulation efficiency^16^. LNC is a biomimetic lipid nanocarrier that is composed of a mixture of oil core coated with a shell composed of solid lipids and emulsifier^15^. In this study, the oil core is composed of labrafac that is medium-chain triglycerides with previously reported biocompatibility, penetration enhancement potential and provides LNC bulkiness^92,93^. Kolliphor® HS 15 is a non-ionic surfactant composed of a mixture of PEG 660 and PEG 660 hydroxystearate that improve LNC stability and stealth features^67,94^. Moreover, the lipid surfactant Epikuron is phosphatidylcholine improves the LNC biocompatibility and shell rigidity^67^. Finally, sodium chloride is usually added during the fabrication of LNC to modulate the phase inversion temperature to suitable range^94^. The major drawback of most of nanoparticles is the burst payload release that could compromise therapeutic efficiency and consequently clinical application^17^. The incorporation of nanocarriers in other carrier as hydrogels or scaffold could overcome this challenge^18^. Hydrogel is a semisolid preparation that showed superior potential in different biomedical applications as tissue regeneration, sustained and local drug release^19^. In addition, hydrogel is a 3-D porous matrix that possess high water content and swelling degree^20^. Interestingly, the incorporation of lipid nanocarriers into the hydrogel is supposed to improve the mechanical properties of the hydrogel as well as prolong drug release^21^.

MS/MS-based molecular networking emerges as a promising technique to supplement compound annotation based on the chemical similarity in MS/MS fragmentation patterns^64^. Thus, we performed further analysis using the Feature-Based Molecular Networking (FBMN) on the GNPS website (http://gnps.ucsd.edu). UHPLC-ESI-QTOF-MS/MS-Based molecular networking guided the tentative identification of 47 compounds. It was noticeable that the highest antioxidant capacity and total phenolics of onion peel extract reflect its richness as a source of antioxidant metabolites. This result perfectly aligns with the findings of LC/MS analysis, which have indicated that onion peel extract displayed the richest content of phenolic compounds. The most abundant bioactive compound classes were phenolic acids, flavonoids and steroidal saponins. It has been reported that the daily intake of phenolics-rich food or food supplements is highly related to the prevention and management of several diseases. Phenolics were either detected at trace levels or not detected in other tested extracts. The role of computational chemistry in drug discovery has significantly increased nowadays^114,115^. Molecular docking as the most widely used technique proposes a new mechanism of action for a particular drug or describes the existing one. Briefly, this helps to save time, effort, and cost in the long journey of new drug development^116,117^. Molecular docking studies against p65 and ATF-2 targets showed that quercetin-3,4′-*O*-diglucoside, alliospiroside C, and alliospiroside D achieved the most promising binding scores and modes. This greatly recommends the expected inhibitory activities of the compounds mentioned above toward p65 and ATF-2 based on their binding affinities. Together, our findings suggest onion peel extract has potential therapeutic values useful for future evaluation for treating osteoarthritis and wounds.

## Conclusion

Breakthroughs in natural products research have shifted the focus of drug discovery and development to bioactive metabolites derived from food and its by-products. This study aimed to determine the chemical composition of selected vegetable by-products (potato, onion, and garlic peels) using liquid chromatography-mass spectrometry (LC–MS). Further, the antioxidant activity was evaluated using 2,2-diphenyl-1-picrylhydrazyl (DPPH) and ferric-reducing antioxidant power (FRAP) assays. The totals of flavonoid and phenolic compounds were also determined. We found that onion peel extract significantly reduced the production of pro-inflammatory cytokines, such as NO, TNF-α, and -IL-6. Moreover, OPE inhibited the expression of MMP-13, COX-2, and iNOS in mouse-isolated chondrocytes, protecting Against IL-1β-mediated ECM degradation. Interestingly, onion peels showed antioxidant capacity as well as the antimicrobial activity against Gram-positive and Gram-negative bacteria. The results also revealed the promising in vitro wound healing activity for onion peel extracts. Onion peel extract reversed LPS-induced pathogenesis in Hfb4 normal skin cells through its anti-inflammatory and antioxidant effects. Furthermore, the nano-formulation revealed no cytotoxicity and enhanced the cellular viability. Moreover, the extracts accelerated the in vivo wound healing process mainly through activation of AP-1 signaling pathway. These findings suggest the potential applications of onion peel extract supported by further in-depth studies as natural pharmaceutical ingredients with antioxidant, anti-osteoarthritis, and wound healing activities.

### Supplementary Information

Supplementary Information.

## Data Availability

Thee data used to support the findings of this study are included within the article.

## References

[1] Pimentel-Moral, S., Cádiz-Gurrea, M. D. L. L., Rodríguez-Pérez, C. & Segura-Carretero, A. In *Functional and Preservative Properties of Phytochemicals* (ed. Bhanu, P.) 209–239 (Academic Press, 2020).

[2] Banerjee J (2017). Bioactives from fruit processing wastes: Green approaches to valuable chemicals. Food Chem..

[3] Marrelli M, Amodeo V, Statti G, Conforti F (2019). Biological properties and bioactive components of *Allium cepa* L.: Focus on potential benefits in the treatment of obesity and related comorbidities. Molecules.

[4] Teshika JD (2019). Traditional and modern uses of onion bulb (*Allium cepa* L.): A systematic review. Crit. Rev. Food Sci. Nutr..

[5] Arshad MS (2017). Status and trends of nutraceuticals from onion and onion by-products: A critical review. Cogent Food Agric..

[6] Celano R (2021). Onion peel: Turning a food waste into a resource. Antioxidants.

[7] Sampaio SL (2020). Potato peels as sources of functional compounds for the food industry: A review. Trends Food Sci. Technol..

[8] Calcio-Gaudino E, Colletti A, Grillo G, Tabasso S, Cravotto G (2020). Emerging processing technologies for the recovery of valuable bioactive compounds from potato peels. Foods.

[9] Gebrechristos HY, Chen W (2018). Utilization of potato peel as eco-friendly products: A review. Food Sci. Nutr..

[10] Kallel F, Ellouz-Chaabouni S (2017). Perspective of garlic processing wastes as low-cost substrates for production of high-added value products: A review. Environ. Progress Sustain. Energy.

[11] Santhosha SG, Jamuna P, Prabhavathi SN (2013). Bioactive components of garlic and their physiological role in health maintenance: A review. Food Biosci..

[12] Sasi M (2021). Garlic (*Allium sativum* L.) bioactives and its role in alleviating oral pathologies. Antioxidants.

[13] Makris DP, Boskou G, Andrikopoulos NK (2007). Polyphenolic content and in vitro antioxidant characteristics of wine industry and other agri-food solid waste extracts. J. Food Compos. Anal..

[14] George D, Maheswari PU, Begum KMMS (2019). Synergic formulation of onion peel quercetin loaded chitosan-cellulose hydrogel with green zinc oxide nanoparticles towards controlled release, biocompatibility, antimicrobial and anticancer activity. Int. J. Biol. Macromol..

[15] Battaglia L, Ugazio E (2019). Lipid nano- and microparticles: An overview of patent-related research. J. Nanomater..

[16] Dabholkar N (2021). Lipid shell lipid nanocapsules as smart generation lipid nanocarriers. J. Mol. Liquids.

[17] Mirzaei BE, Ramazani SAA, Shafiee M, Danaei M (2013). Studies on glutaraldehyde crosslinked chitosan hydrogel properties for drug delivery systems. Int. J. Polym. Mater. Polym. Biomater..

[18] Sanad RA-B, Abdel-Bar HM (2017). Chitosan–hyaluronic acid composite sponge scaffold enriched with Andrographolide-loaded lipid nanoparticles for enhanced wound healing. Carbohyd. Polym..

[19] Vashist A, Vashist A, Gupta YK, Ahmad S (2014). Recent advances in hydrogel based drug delivery systems for the human body. J. Mater. Chem. B.

[20] Biondi M, Borzacchiello A, Mayol L, Ambrosio L (2015). Nanoparticle-integrated hydrogels as multifunctional composite materials for biomedical applications. Gels.

[21] Abdel-Bar HM, Osman R, Abdel-Reheem AY, Mortada N, Awad GAS (2016). Tunable biodegradable nanocomposite hydrogel for improved cisplatin efficacy on HCT-116 colorectal cancer cells and decreased toxicity in rats. Biomacromol.

[22] Abouzed TK (2018). Red onion scales ameliorated streptozotocin-induced diabetes and diabetic nephropathy in Wistar rats in relation to their metabolite fingerprint. Diabetes Res. Clin. Pract..

[23] Hussein ME (2022). Identification of antibacterial metabolites from endophytic fungus *Aspergillus fumigatus*, isolated from *Albizia lucidior* leaves (Fabaceae), utilizing metabolomic and molecular docking techniques. Molecules.

[24] Katajamaa M, Miettinen J, Orešič M (2006). MZmine: toolbox for processing and visualization of mass spectrometry based molecular profile data. Bioinformatics.

[25] Pluskal T, Castillo S, Villar-Briones A, Orešič M (2010). MZmine 2: modular framework for processing, visualizing, and analyzing mass spectrometry-based molecular profile data. BMC Bioinform..

[26] Hussein ME (2022). Anticholinesterase activity of budmunchiamine alkaloids revealed by comparative chemical profiling of two *Albizia* spp., molecular docking and dynamic studies. Plants.

[27] Otify AM (2023). bioherbicidal activity and metabolic profiling of allelopathic metabolites of three cassia species using UPLC-qTOF-MS/MS and molecular networking. Metabolomics.

[28] Nothias L-F (2020). Feature-based molecular networking in the GNPS analysis environment. Nat. Methods.

[29] Shannon P (2003). Cytoscape: A software environment for integrated models of biomolecular interaction networks. Genome Res..

[30] Boly R, Lamkami T, Lompo M, Dubois J, Guissou I (2016). DPPH free radical scavenging activity of two extracts from Agelanthus dodoneifolius (Loranthaceae) leaves. Int. J. Toxicol. Pharmacol. Res..

[31] Chen Z, Bertin R, Froldi G (2013). EC50 estimation of antioxidant activity in DPPH assay using several statistical programs. Food Chem..

[32] Benzie IFF, Strain JJ (1996). The ferric reducing ability of plasma (FRAP) as a Measure of “Antioxidant Power”: The FRAP assay. Anal. Biochem..

[33] Attard E (2013). A rapid microtitre plate Folin-Ciocalteu method for the assessment of polyphenols. Cent. Eur. J. Biol..

[34] Kiranmai M, Mahendra CBK, Ibrahim M (2011). Comparison of total flavonoid content of Azadirachta indica root bark extracts prepared by different methods of extraction. Res. J. Pharm. Biol. Chem. Sci..

[35] Wu D (2019). The protective effect of sophocarpine in osteoarthritis: An in vitro and in vivo study. Int. Immunopharmacol..

[36] Ibrahim NM, Fahim SH, Hassan M, Farag AE, Georgey HH (2022). Design and synthesis of ciprofloxacin-sulfonamide hybrids to manipulate ciprofloxacin pharmacological qualities: Potency and side effects. Eur. J. Med. Chem..

[37] Ismail MM (2021). Exploring the antivirulence activity of pulverulentone A, a phloroglucinol-derivative from *Callistemon citrinus* leaf extract, against multi-drug resistant *Pseudomonas aeruginosa*. Antibiotics.

[38] Humphries RM (2018). CLSI methods development and standardization working group best practices for evaluation of antimicrobial susceptibility tests. J. Clin. Microbiol..

[39] Salem MA, El-Shiekh RA, Hashem RA, Hassan M (2021). In vivo antibacterial activity of star anise (*Illicium verum* Hook.) extract using murine MRSA skin infection model in relation to its metabolite profile. Infect. Drug Resist..

[40] Mouzouvi CRA, Umerska A, Bigot AK, Saulnier P (2017). Surface active properties of lipid nanocapsules. PLoS ONE.

[41] Safwat S, Hathout RM, Ishak RA, Mortada ND (2017). Augmented simvastatin cytotoxicity using optimized lipid nanocapsules: A potential for breast cancer treatment. J. Liposome Res..

[42] Sigrist RMS, Liau J, Kaffas AE, Chammas MC, Willmann JK (2017). Ultrasound elastography: Review of techniques and clinical applications. Theranostics.

[43] Ciardi M (2021). Effective and selective extraction of quercetin from onion (*Allium cepa* L.) skin waste using water dilutions of acid-based deep eutectic solvents. Materials.

[44] Abdel-Bar HM, Walters AA, Wang JT-W, Al-Jamal KT (2021). Combinatory delivery of etoposide and siCD47 in a lipid polymer hybrid delays lung tumor growth in an experimental melanoma lung metastatic model. Adv. Healthcare Mater..

[45] Pereira RL (2016). Hydrogel containing adapalene- and dapsone-loaded lipid-core nanocapsules for cutaneous application: Development, characterization, in vitro irritation and permeation studies. Drug Dev. Ind. Pharm..

[46] Khan MFA (2022). Hydrogel containing solid lipid nanoparticles loaded with argan oil and simvastatin: Preparation, in vitro and ex vivo assessment. Gels.

[47] Zeb A (2017). Enhanced anti-rheumatic activity of methotrexate-entrapped ultradeformable liposomal gel in adjuvant-induced arthritis rat model. Int. J. Pharm..

[48] Siddiqui B, Rehman AU, Haq I-U, Ahmad NM, Ahmed N (2020). Development, optimisation, and evaluation of nanoencapsulated diacerein emulgel for potential use in osteoarthritis. J. Microencapsul..

[49] Shi H (2016). The in vitro effect of lipopolysaccharide on proliferation, inflammatory factors and antioxidant enzyme activity in bovine mammary epithelial cells. Anim. Nutr..

[50] Murthy SRS (2013). Evaluation of in vivo wound healing activity of bacopa monniera on different wound model in rats. BioMed. Res. Int..

[51] Al-Karmalawy AA (2021). Naturally available flavonoid aglycones as potential antiviral drug candidates against SARS-CoV-2. Molecules.

[52] Shoala T (2021). Nanobiotechnological approaches to enhance potato resistance against potato leafroll virus (PLRV) using glycyrrhizic acid ammonium salt and salicylic acid nanoparticles. Horticulturae.

[53] Hamed MIA (2021). β-Blockers bearing hydroxyethylamine and hydroxyethylene as potential SARS-CoV-2 Mpro inhibitors: Rational based design, in silico, in vitro, and SAR studies for lead optimization. RSC Adv..

[54] Taher RF (2021). Two new flavonoids and anticancer activity of Hymenosporum flavum: In vitro and molecular docking studies. J. Herbmed. Pharmacol..

[55] Chen Y-Q, Ghosh S, Ghosh G (1998). A novel DNA recognition mode by the NF-κB p65 homodimer. Nat. Struct. Biol..

[56] Kirsch K (2020). Co-regulation of the transcription controlling ATF2 phosphoswitch by JNK and p38. Nat. Commun..

[57] Hazem RM (2022). Pirfenidone and vitamin D mitigate renal fibrosis induced by doxorubicin in mice with Ehrlich solid tumor. Life Sci..

[58] Elia SG, Al-Karmalawy AA, Nasr MY, Elshal MF (2022). Loperamide potentiates doxorubicin sensitivity in triple-negative breast cancer cells by targeting MDR1 and JNK and suppressing mTOR and Bcl-2: In vitro and molecular docking study. J. Biochem. Mol. Toxicol..

[59] Aziz MA, Shehab WS, Al-Karmalawy AA, El-Farargy AF, Abdellattif MH (2021). Design, synthesis, biological evaluation, 2D-QSAR modeling, and molecular docking studies of novel 1H-3-indolyl derivatives as significant antioxidants. Int. J. Mol. Sci..

[60] Raslan MA (2021). *Cordyline fruticosa* (L.) A. Chev. leaves: Isolation, HPLC/MS profiling and evaluation of nephroprotective and hepatoprotective activities supported by molecular docking. New J. Chem..

[61] Diab RT, Abdel-Sami ZK, Abdel-Aal EH, Al-Karmalawy AA, Abo-Dya NE (2021). Design and synthesis of a new series of 3,5-disubstituted-1,2,4-oxadiazoles as potential colchicine binding site inhibitors: antiproliferative activity, molecular docking, and SAR studies. New J. Chem..

[62] El-Masry RM (2022). Newly synthesized series of oxoindole–oxadiazole conjugates as potential anti-SARS-CoV-2 agents: In silico and in vitro studies. New J. Chem..

[63] Pang Z (2021). MetaboAnalyst 50: Narrowing the gap between raw spectra and functional insights. Nucleic Acids Res..

[64] Wang M (2016). Sharing and community curation of mass spectrometry data with Global Natural Products Social Molecular Networking. Nat. Biotechnol..

[65] Ansari MY, Ahmad N, Haqqi TM (2020). Oxidative stress and inflammation in osteoarthritis pathogenesis: Role of polyphenols. Biomed. Pharmacother..

[66] Guimarães I, Baptista-Silva S, Pintado M, Oliveira A (2021). Polyphenols: A promising avenue in therapeutic solutions for wound care. Appl. Sci..

[67] Huynh NT, Passirani C, Saulnier P, Benoit JP (2009). Lipid nanocapsules: A new platform for nanomedicine. Int. J. Pharm..

[68] Li X, Yang Q, Zhao Y, Long S, Zheng J (2017). Dual physically crosslinked double network hydrogels with high toughness and self-healing properties. Soft Matter.

[69] Archana D, Singh BK, Dutta J, Dutta PK (2015). Chitosan-PVP-nano silver oxide wound dressing: In vitro and in vivo evaluation. Int. J. Biol. Macromol..

[70] Tang C, Yin L, Yu J, Yin C, Pei Y (2007). Swelling behavior and biocompatibility of Carbopol-containing superporous hydrogel composites. J. Appl. Polym. Sci..

[71] Migliozzi S, Meridiano G, Angeli P, Mazzei L (2020). Investigation of the swollen state of Carbopol molecules in non-aqueous solvents through rheological characterization. Soft Matter.

[72] Ong KL, Kaur G, Pensupa N, Uisan K, Lin CSK (2018). Trends in food waste valorization for the production of chemicals, materials and fuels: Case study South and Southeast Asia. Biores. Technol..

[73] Isah S, Ozbay G (2020). Valorization of food loss and wastes: Feedstocks for biofuels and valuable chemicals. Front. Sustain. Food Syst..

[74] Sharma M, Usmani Z, Gupta VK, Bhat R (2021). Valorization of fruits and vegetable wastes and by-products to produce natural pigments. Crit. Rev. Biotechnol..

[75] Saini A, Panesar PS, Bera MB (2019). Valorization of fruits and vegetables waste through green extraction of bioactive compounds and their nanoemulsions-based delivery system. Bioresourc. Bioprocess..

[76] Sabater C, Ruiz L, Delgado S, Ruas-Madiedo P, Margolles A (2020). Valorization of vegetable food waste and by-products through fermentation processes. Front. Microbiol..

[77] Samvelyan HJ, Hughes D, Stevens C, Staines KA (2021). Models of osteoarthritis: Relevance and new insights. Calcif. Tissue Int..

[78] Di Nicola V (2020). Degenerative osteoarthritis a reversible chronic disease. Regener. Therapy.

[79] Steinmeyer J, Bock F, Stöve J, Jerosch J, Flechtenmacher J (2018). Pharmacological treatment of knee osteoarthritis: Special considerations of the new German guideline. Orthoped. Rev..

[80] Weng C, Xu J, Wang Q, Lu W, Liu Z (2020). Efficacy and safety of duloxetine in osteoarthritis or chronic low back pain: A Systematic review and meta-analysis. Osteoarthr. Cartil..

[81] Mu P (2022). Botanical drug extracts combined with biomaterial carriers for osteoarthritis cartilage degeneration treatment: A review of 10 years of research. Front. Pharmacol..

[82] Choi MC, Jo J, Park J, Kang HK, Park Y (2019). NF-κB signaling pathways in osteoarthritic cartilage destruction. Cells.

[83] Rigoglou S, Papavassiliou AG (2013). The NF-κB signalling pathway in osteoarthritis. Int. J. Biochem. Cell Biol..

[84] Mulani MS, Kamble EE, Kumkar SN, Tawre MS, Pardesi KR (2019). Emerging strategies to combat ESKAPE pathogens in the era of antimicrobial resistance: A review. Front. Microbiol..

[85] Anand U (2022). Ethnodermatological use of medicinal plants in India: From ayurvedic formulations to clinical perspectives—a review. J. Ethnopharmacol..

[86] Ali NB (2023). In vitro and in vivo antibiofilm activity of red onion scales: An agro-food waste. Molecules.

[87] Ismail MM (2021). Exploring the antivirulence activity of pulverulentone A, a phloroglucinol-derivative from *Callistemon citrinus* leaf extract, against multi-drug resistant *Pseudomonas aeruginosa*. Antibiotics.

[88] El-Shiekh RA, Hassan M, Hashem RA, Abdel-Sattar E (2021). Bioguided isolation of antibiofilm and antibacterial pregnane glycosides from caralluma quadrangula: Disarming multidrug-resistant pathogens. Antibiotics.

[89] Mosalam EM, Zidan A-AA, Mehanna ET, Mesbah NM, Abo-Elmatty DM (2020). Thymoquinone and pentoxifylline enhance the chemotherapeutic effect of cisplatin by targeting Notch signaling pathway in mice. Life Sci..

[90] Abo-Mansour HE (2021). Ginger extract loaded into chitosan nanoparticles enhances cytotoxicity and reduces cardiotoxicity of doxorubicin in hepatocellular carcinoma in mice. Nutr. Cancer.

[91] Kim J, Kim J-S, Park E (2013). Cytotoxic and anti-inflammatory effects of onion peel extract on lipopolysaccharide stimulated human colon carcinoma cells. Food Chem. Toxicol..

[92] Niu Z, Conejos-Sánchez I, Griffin BT, O’Driscoll CM, Alonso MJ (2016). Lipid-based nanocarriers for oral peptide delivery. Adv. Drug Deliv. Rev..

[93] Heurtault B (2003). The influence of lipid nanocapsule composition on their size distribution. Eur. J. Pharm. Sci..

[94] Anton N, Gayet P, Benoit J-P, Saulnier P (2007). Nano-emulsions and nanocapsules by the PIT method: An investigation on the role of the temperature cycling on the emulsion phase inversion. Int. J. Pharm..

[95] Elhassaneen Y, Elhady YA (2014). Onion peel powder alleviate acrylamide-induced cytotoxicity and immunotoxicity in liver cell culture. Life Sci. J..

[96] Gad D (2022). Biostimulants improve the hepatoprotection of Ammi visnaga seed yield extract against carbon tetrachloride induced acute hepatitis in mice through modulation of MAPK. Saudi J. Biol. Sci..

[97] Batista CRA, Gomes GF, Candelario-Jalil E, Fiebich BL, de Oliveira ACP (2019). Lipopolysaccharide-induced neuroinflammation as a bridge to understand neurodegeneration. Int. J. Mol. Sci..

[98] Tang X (2022). Polystyrene nanoplastics exacerbated lipopolysaccharide-induced necroptosis and inflammation via the ROS/MAPK pathway in mice spleen. Environ. Toxicol..

[99] Beheshti F, Hosseini M, Arab Z, Asghari A, Anaeigoudari A (2022). Ameliorative role of metformin on lipopolysaccharide-mediated liver malfunction through suppression of inflammation and oxidative stress in rats. Toxin Rev..

[100] Dhlamini Q (2022). FGF1 alleviates LPS-induced acute lung injury via suppression of inflammation and oxidative stress. Mol. Med..

[101] Maleki SJ, Crespo JF, Cabanillas B (2019). Anti-inflammatory effects of flavonoids. Food Chem..

[102] Albishi T, John JA, Al-Khalifa AS, Shahidi F (2013). Antioxidant, anti-inflammatory and DNA scission inhibitory activities of phenolic compounds in selected onion and potato varieties. J. Funct. Foods.

[103] Shabir I (2022). Nutritional profile, phytochemical compounds, biological activities, and utilisation of onion peel for food applications: A review. Sustainability.

[104] Masood S (2021). Investigation of the anti-hyperglycemic and antioxidant effects of wheat bread supplemented with onion peel extract and onion powder in diabetic rats. J. Diabetes Metab. Disord..

[105] Lee KA (2011). Antimicrobial and antioxidative effects of onion peel extracted by the subcritical water. Food Sci. Biotechnol..

[106] Yeh C-J (2017). The effects of artocarpin on wound healing: In vitro and in vivo studies. Sci. Rep..

[107] Bjørge IM, Kim SY, Mano JF, Kalionis B, Chrzanowski W (2017). Extracellular vesicles, exosomes and shedding vesicles in regenerative medicine—a new paradigm for tissue repair. Biomater. Sci..

[108] Hade MD, Suire CN, Mossell J, Suo Z (2022). Extracellular vesicles: Emerging frontiers in wound healing. Med. Res. Rev..

[109] Assaw S (2012). The use of modified Massion's trichrome staining in collagen evaluation in wound healing study. Malays. J. Vet. Res..

[110] Gazon H, Barbeau B, Mesnard J-M, Peloponese J-M (2018). Hijacking of the AP-1 signaling pathway during development of ATL. Front. Microbiol..

[111] Uluçkan Ö, Guinea-Viniegra J, Jimenez M, Wagner EF (2015). Signalling in inflammatory skin disease by AP-1 (Fos/Jun). Clin. Exp. Rheumatol..

[112] Ha AT (2022). Anti-inflammatory, antioxidant, moisturizing, and antimelanogenesis effects of quercetin 3-O-beta-D-glucuronide in human keratinocytes and melanoma cells via activation of NF-kappaB and AP-1 pathways. Int. J. Mol. Sci..

[113] Lee S-H, Cho JH, Park J-H, Cho J-S, Lee H-M (2021). High mobility group box chromosomal protein-1 induces myofibroblast differentiation and extracellular matrix production via RAGE, p38, JNK and AP-1 signaling pathways in nasal fibroblasts. Am. J. Rhinol. Allergy.

[114] El-Demerdash A (2021). Investigating the structure–activity relationship of marine natural polyketides as promising SARS-CoV-2 main protease inhibitors. RSC Adv..

[115] El-Gizawy HA (2021). *Pimenta dioica* (L.) Merr. bioactive constituents exert anti-SARS-CoV-2 and anti-inflammatory activities: Molecular docking and dynamics, in vitro, and in vivo studies. Molecules.

[116] Ma C (2021). Design and synthesis of new quinoxaline derivatives as potential histone deacetylase inhibitors targeting hepatocellular carcinoma: in silico, in vitro, and SAR studies. Front. Chem..

[117] Khattab M, Al-Karmalawy AA (2021). Computational repurposing of benzimidazole anthelmintic drugs as potential colchicine binding site inhibitors. Future Med. Chem..

