# Supplementary material for: Investigation of the phytochemical composition, antioxidant, antibacterial, anti-osteoarthritis, and wound healing activities of selected vegetable waste
Source: Sci Rep. 2023 Aug 10;13:13034. doi: 10.1038/s41598-023-38591-y (PMC10415269; doi:10.1038/s41598-023-38591-y)
Supplement: Supplementary file 1 — Supplementary Information. [file 41598_2023_38591_MOESM1_ESM.pdf]

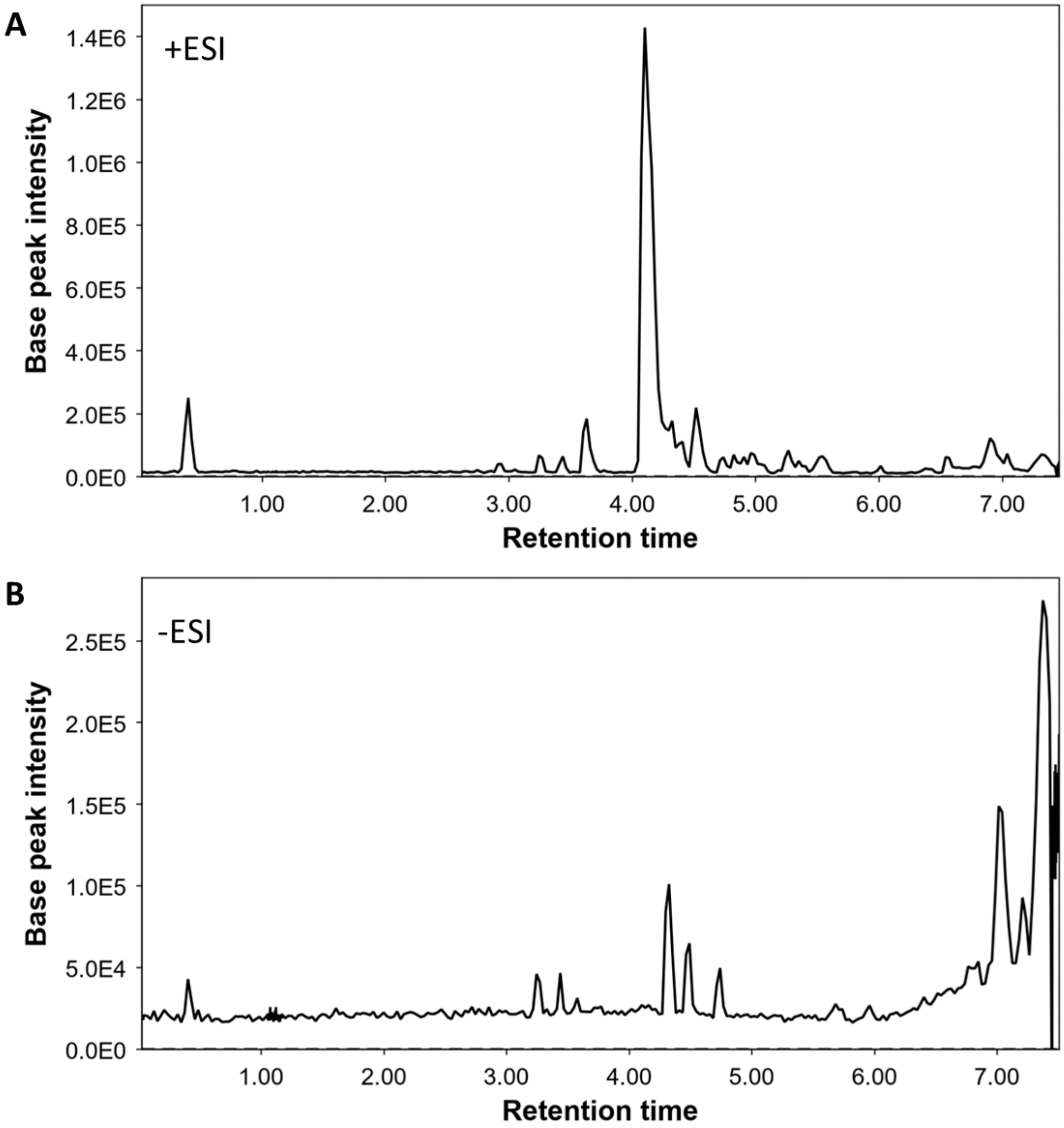

Figure S1. Base peak chromatograms (BPC) of garlic peels extract as analyzed by LC/MS in positive (A) and negative (B) ionization modes.

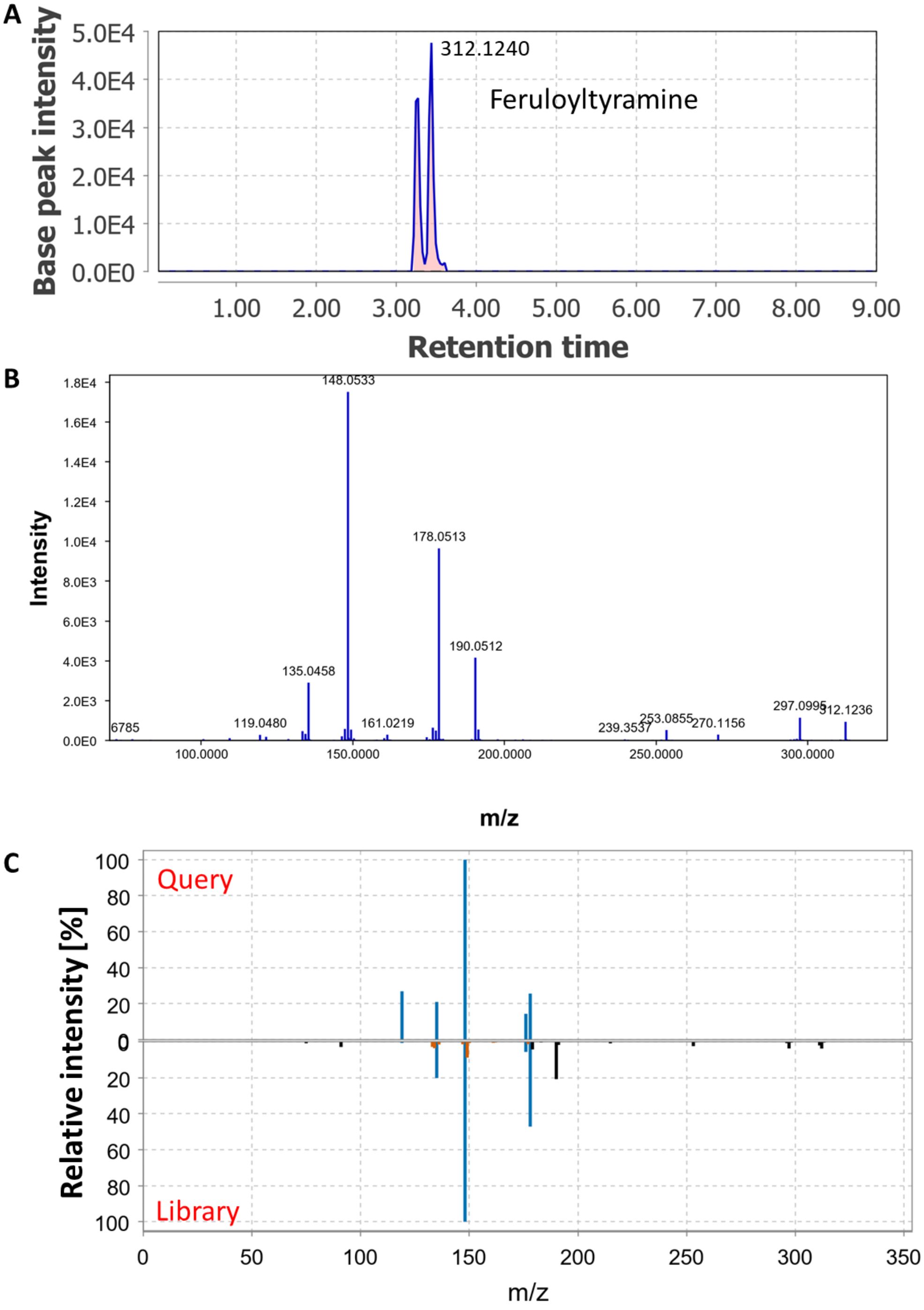

Figure S2. Extracted ion chromatograms (EIC) (A), MS spectrum (B), and MS/MS mirror match with GNPS library (C) of feruloyltyramine identified from garlic peels extract as analyzed by LC/MS in negative ionization mode.

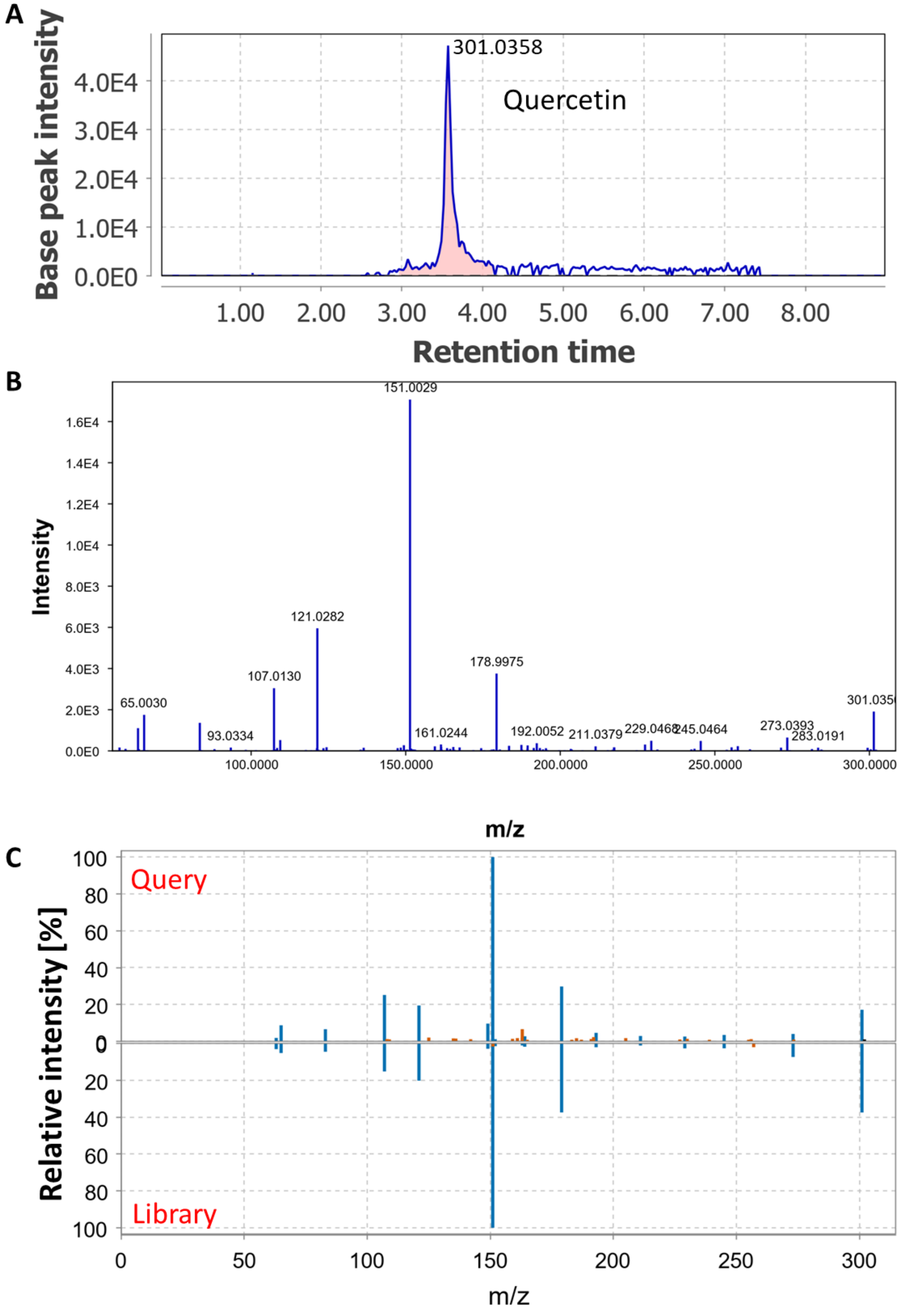

Figure S3. Extracted ion chromatograms (EIC) (A), MS spectrum (B) and MS/MS mirror match with GNPS library (C) of quercetin identified from garlic peels extract as analyzed by LC/MS in negative ionization mode.

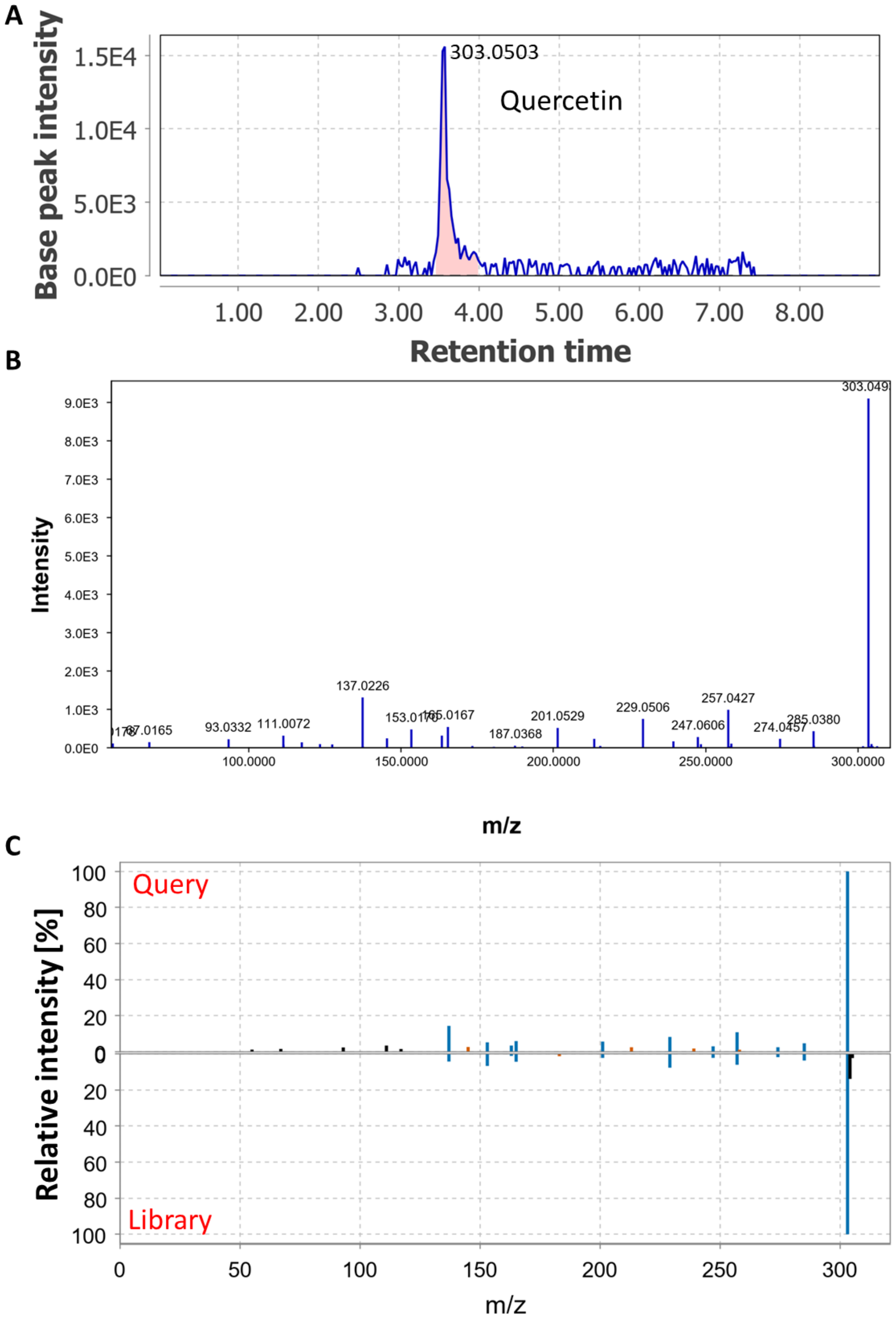

Figure S4. Extracted ion chromatograms (EIC) (A), MS spectrum (B) and MS/MS mirror match with GNPS library (C) of quercetin identified from garlic peels extract as analyzed by LC/MS in positive ionization mode.

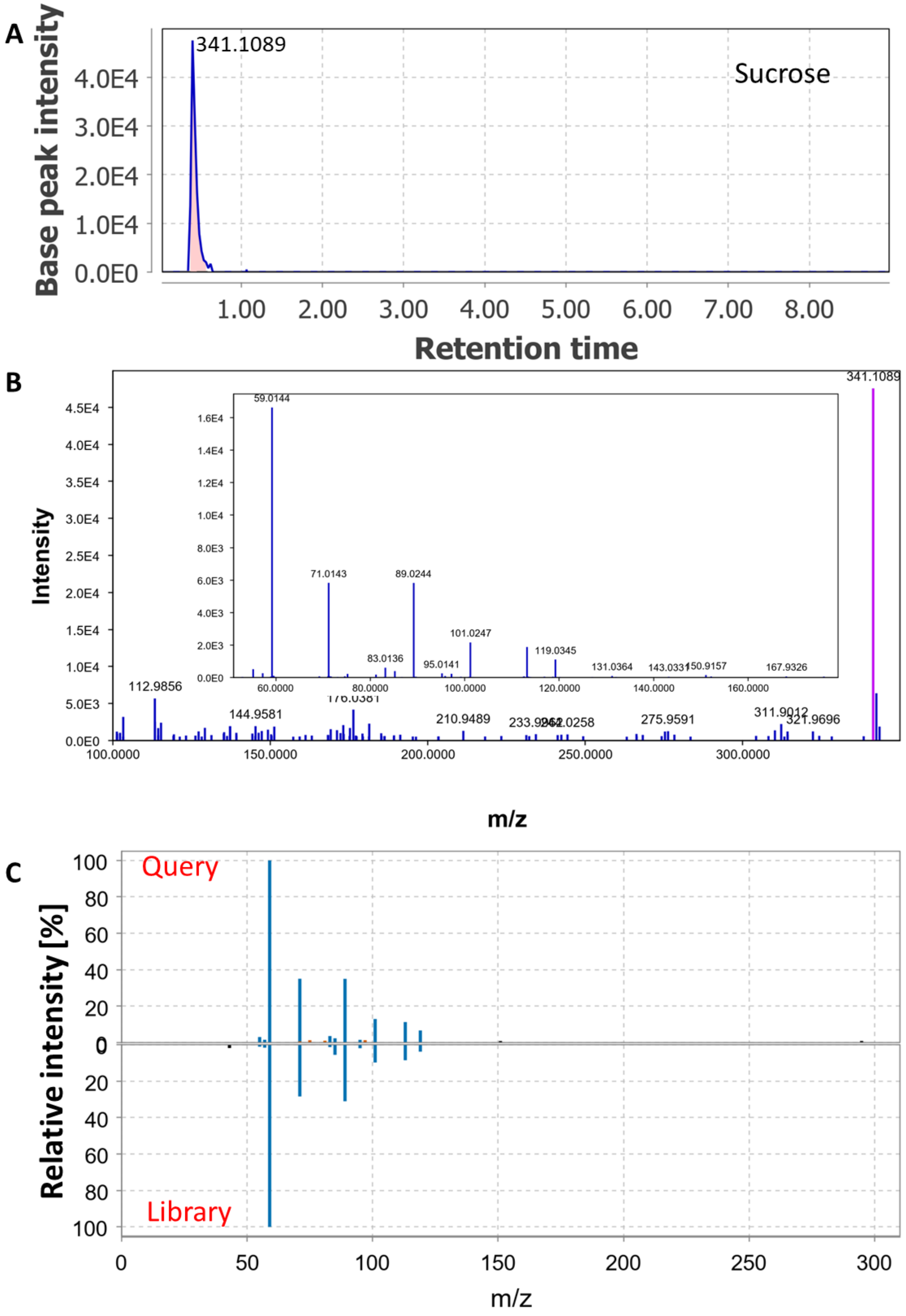

Figure S5. Extracted ion chromatograms (EIC) (A), MS spectrum (B) and MS/MS mirror match with GNPS library (C) of sucrose identified from garlic peels extract as analyzed by LC/MS in negative ionization mode.

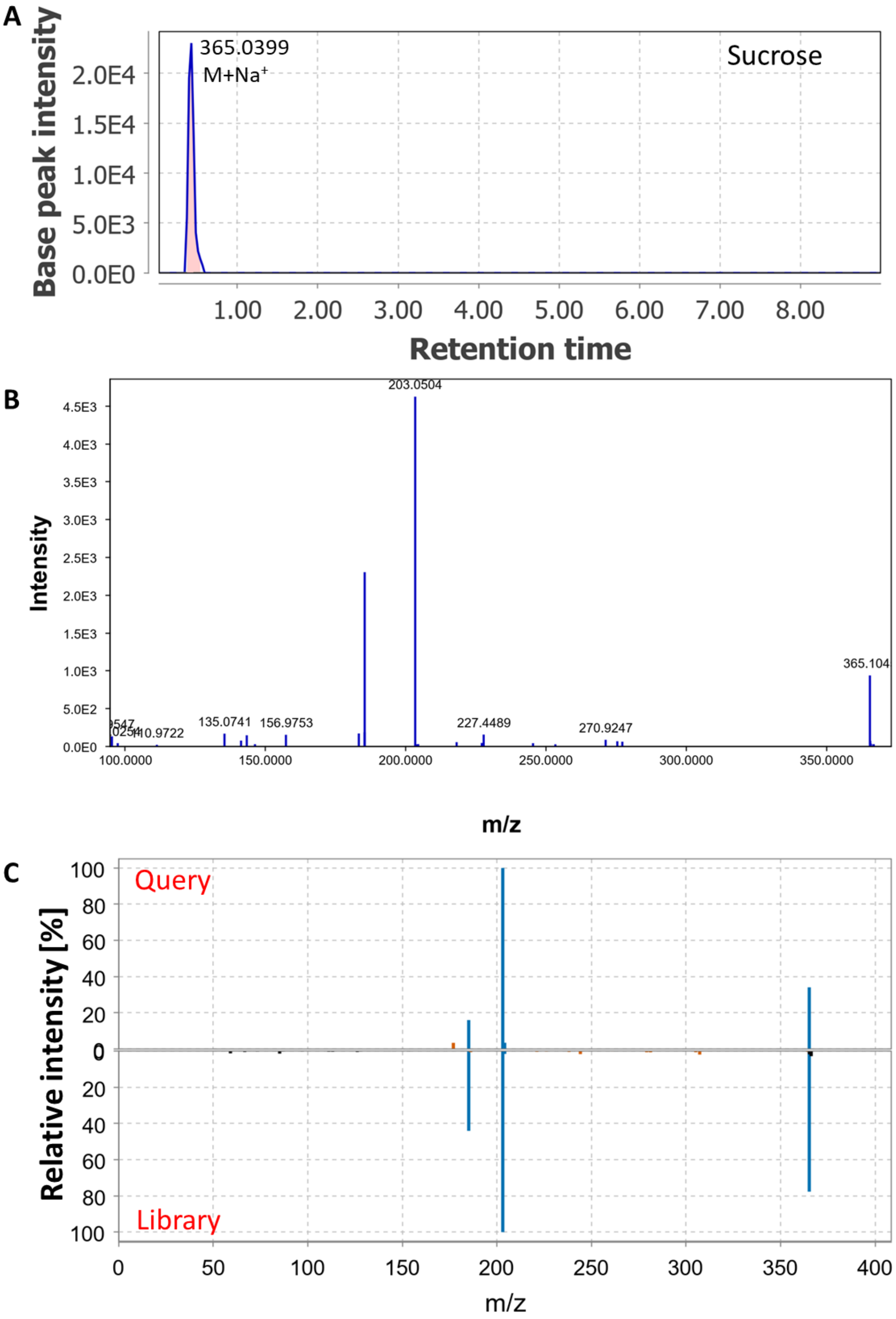

Figure S6. Extracted ion chromatograms (EIC) (A), MS spectrum (B) and MS/MS mirror match with GNPS library (C) of sucrose identified from garlic peels extract as analyzed by LC/MS in positive ionization mode.

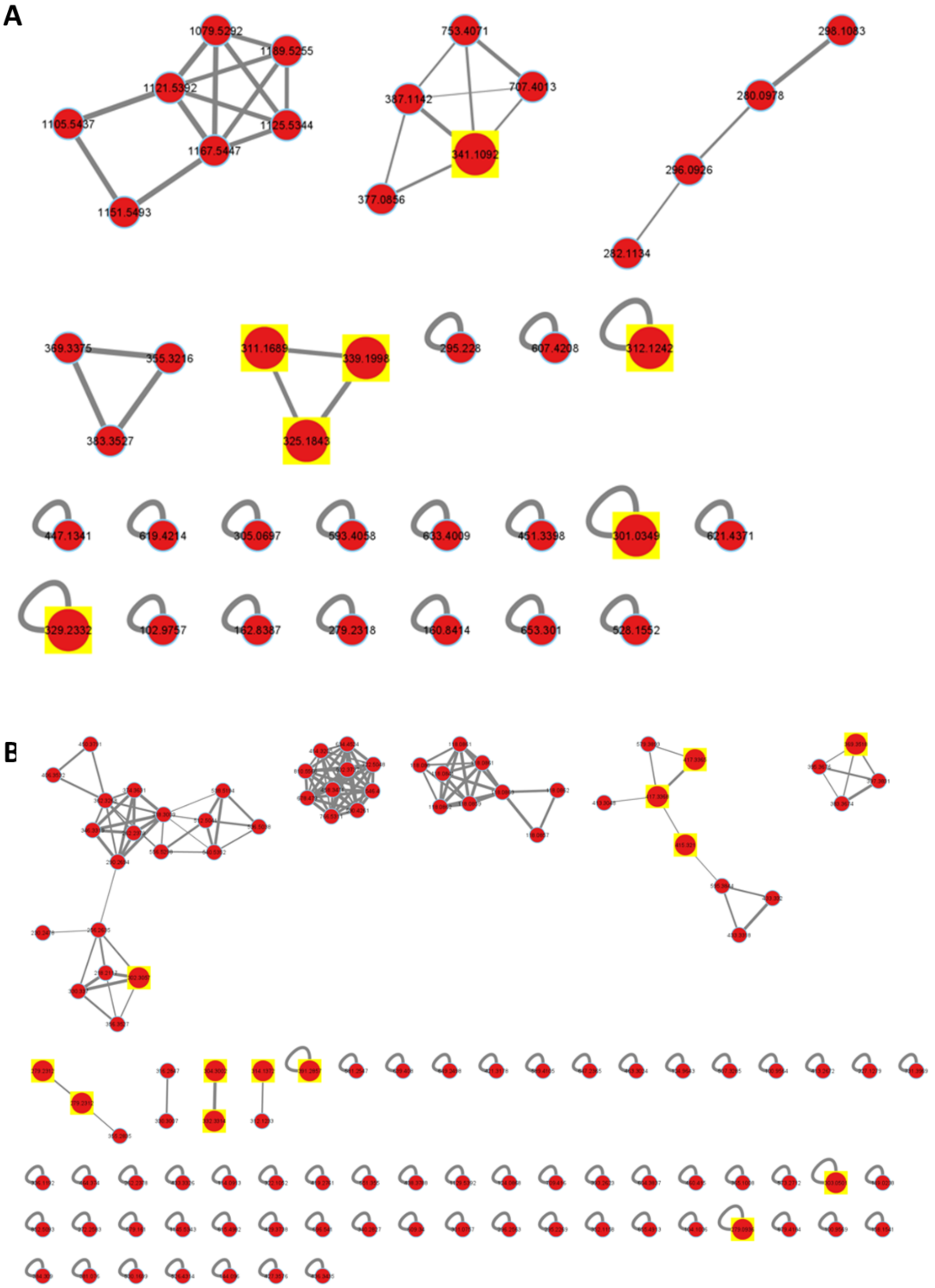

Figure S7. Molecular network of features extracted from LC-MS data ionized in negative (A) and positive (B) ionization modes from garlic peels extract.

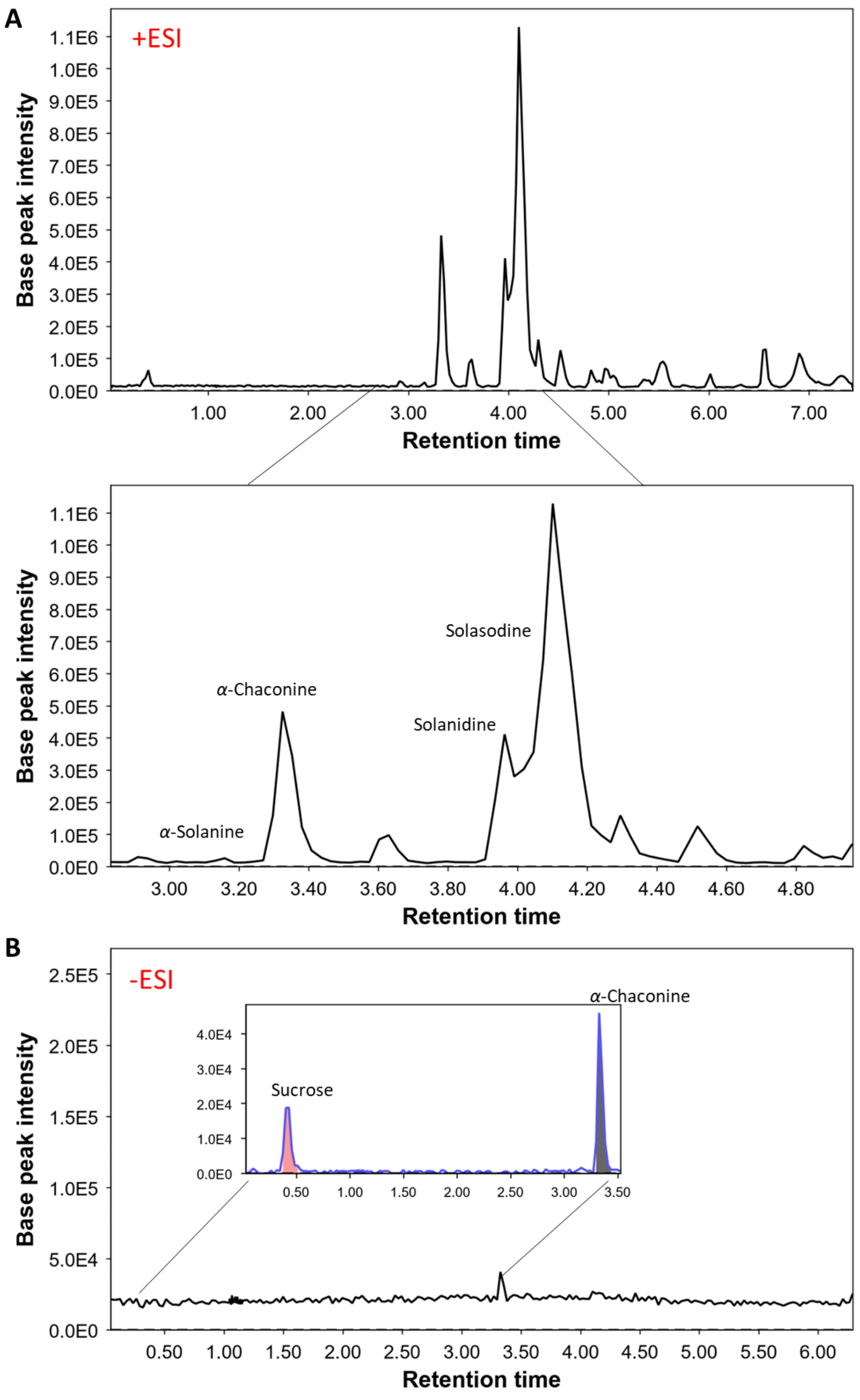

Figure S8. Base peak chromatograms (TIC) of potato peels extract as analyzed by LC/MS in positive (A) and negative (B) ionization modes.

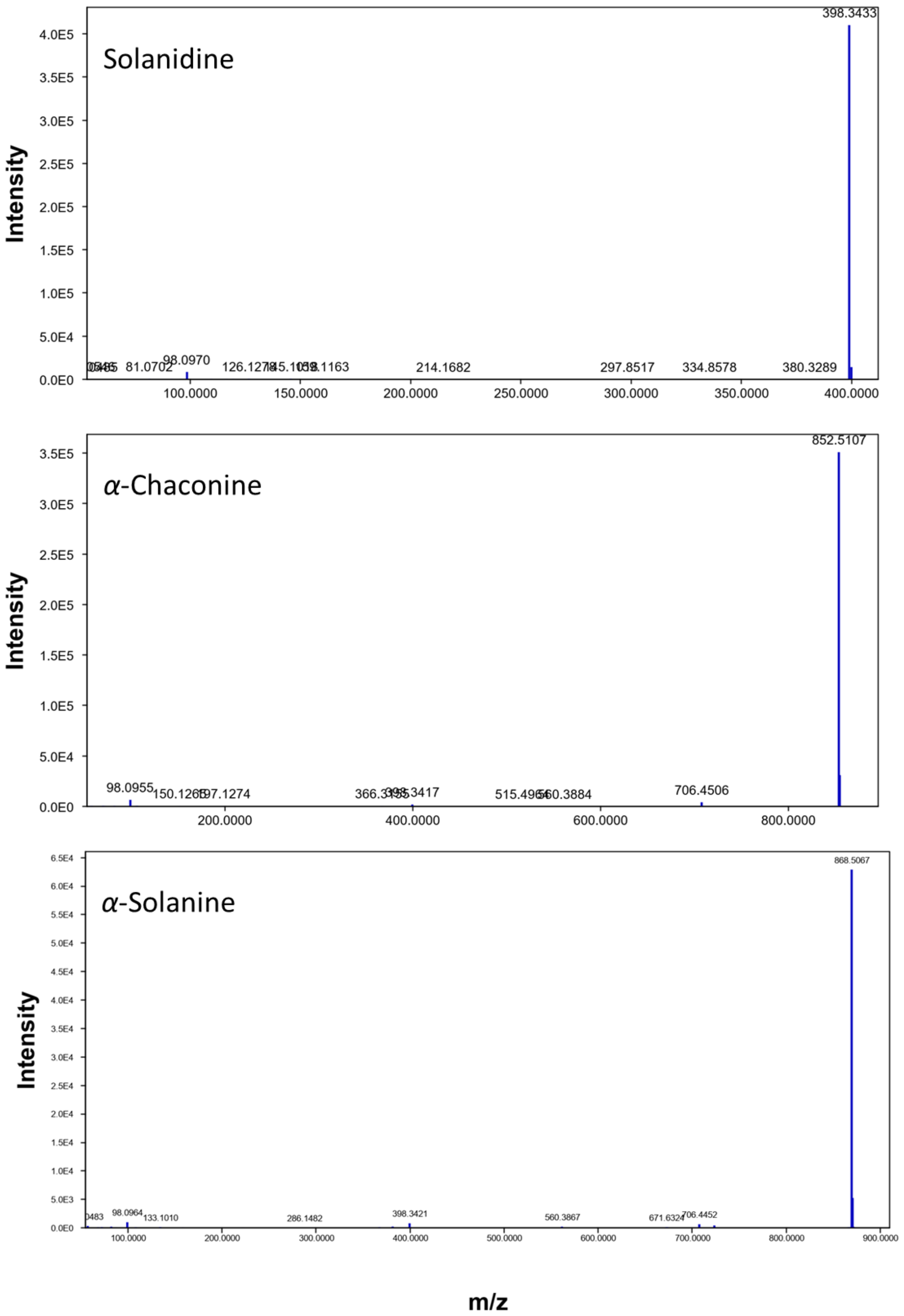

Solanidine
398.3433
98.0970
81.0702
126.1278
214.1682
297.8517
334.8578
380.3289
α-Chaconine
852.5107
98.0955
706.4506
α-Solanine
868.5067
98.0964
133.1010
286.1482
398.3421
560.3867
671.6324
706.4452
Intensity
m/z

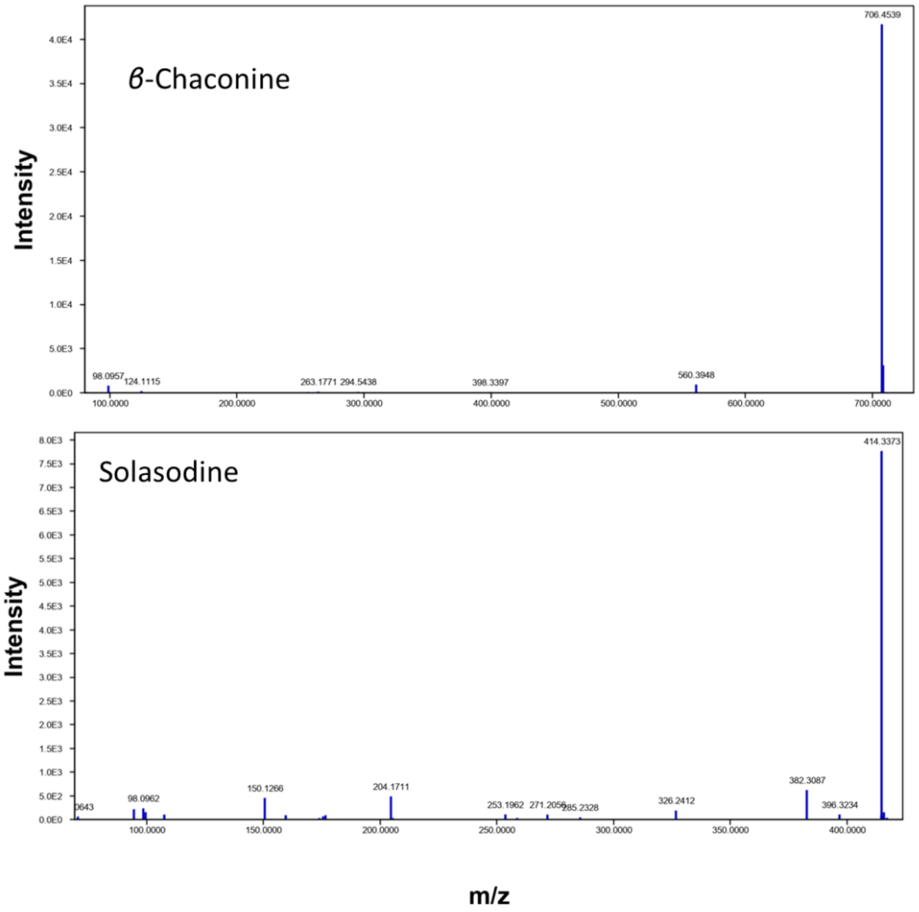

Figure S9. MS/MS spectra of identified alkaloids from potato peels extract as analyzed by LC/MS in positive ionization mode.

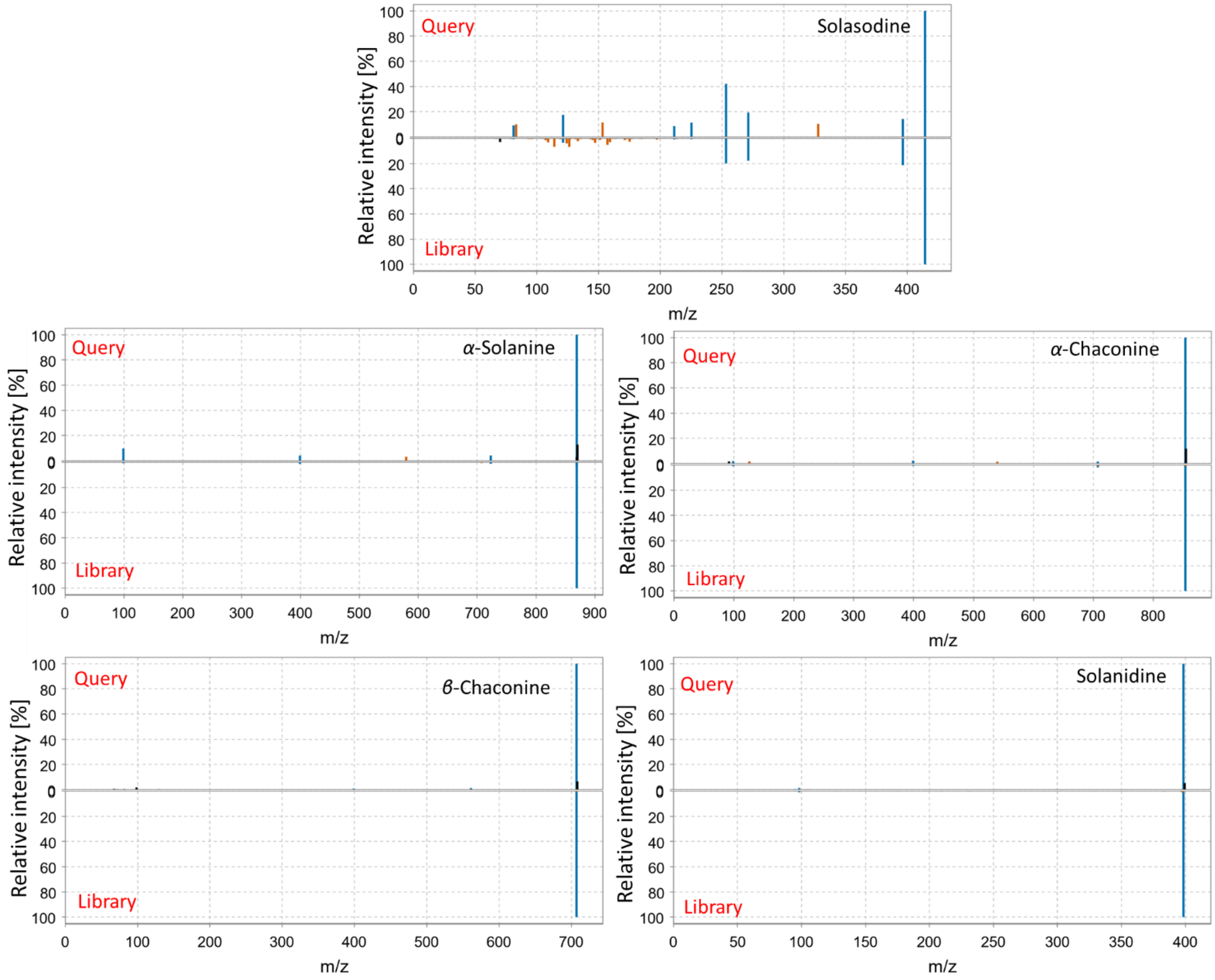

Figure S10. MONA library matched MS/MS spectrum of alkaloids identified from potato peel extract as analyzed by LC/MS in positive ionization mode.

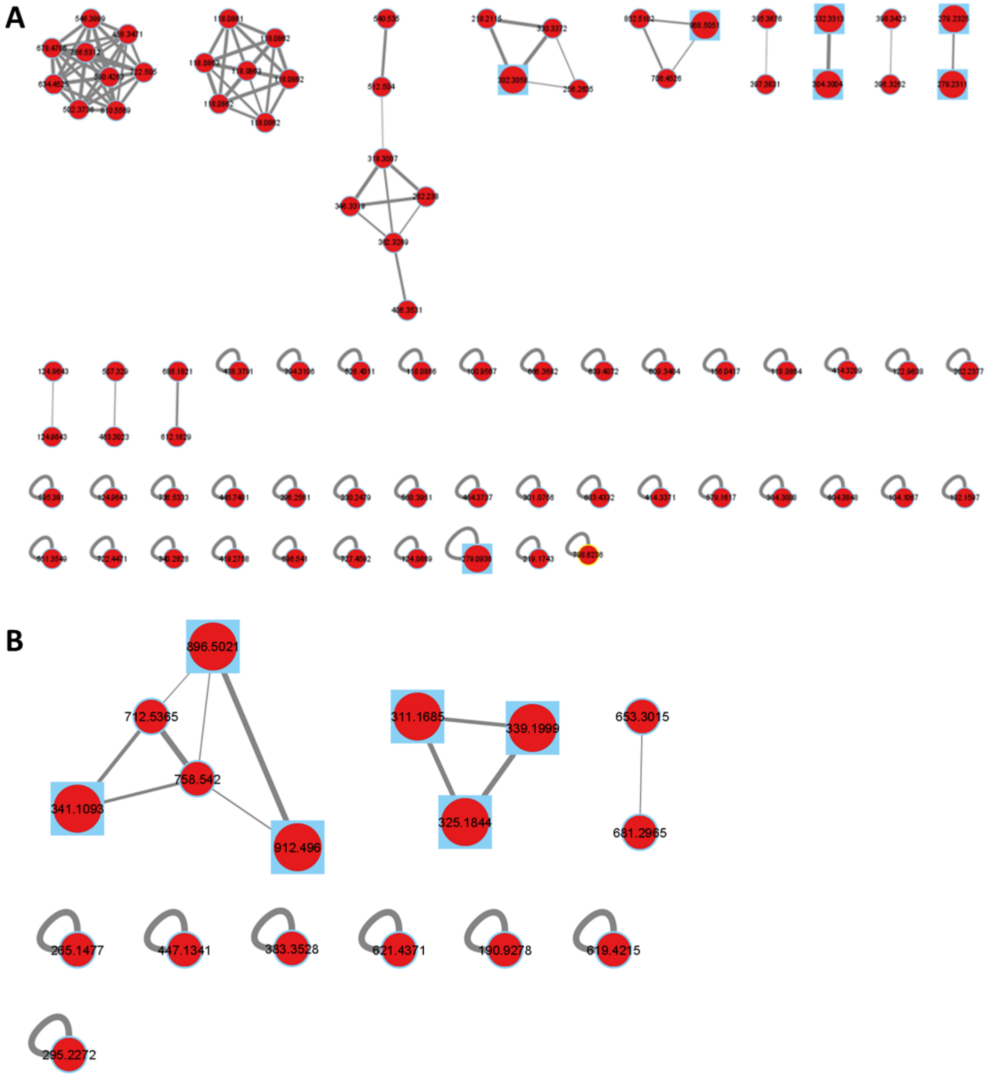

Figure S11. Molecular network of features extracted from LC-MS data ionized in positive (A) and negative (B) ionization modes from potato peel extract.

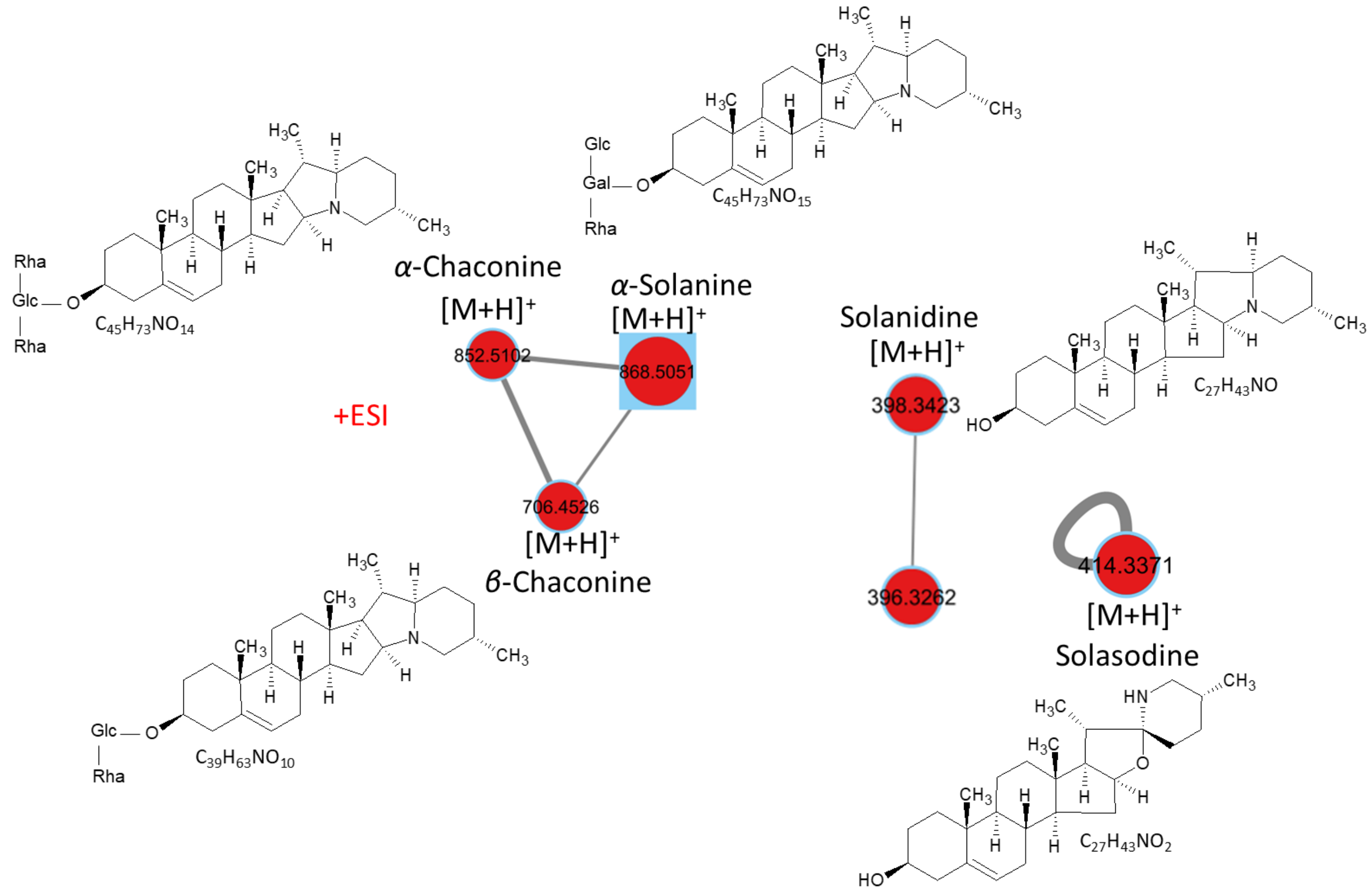

Figure S12. Selected sub-network of alkaloids identified from potato peel extract in positive ionization mode.

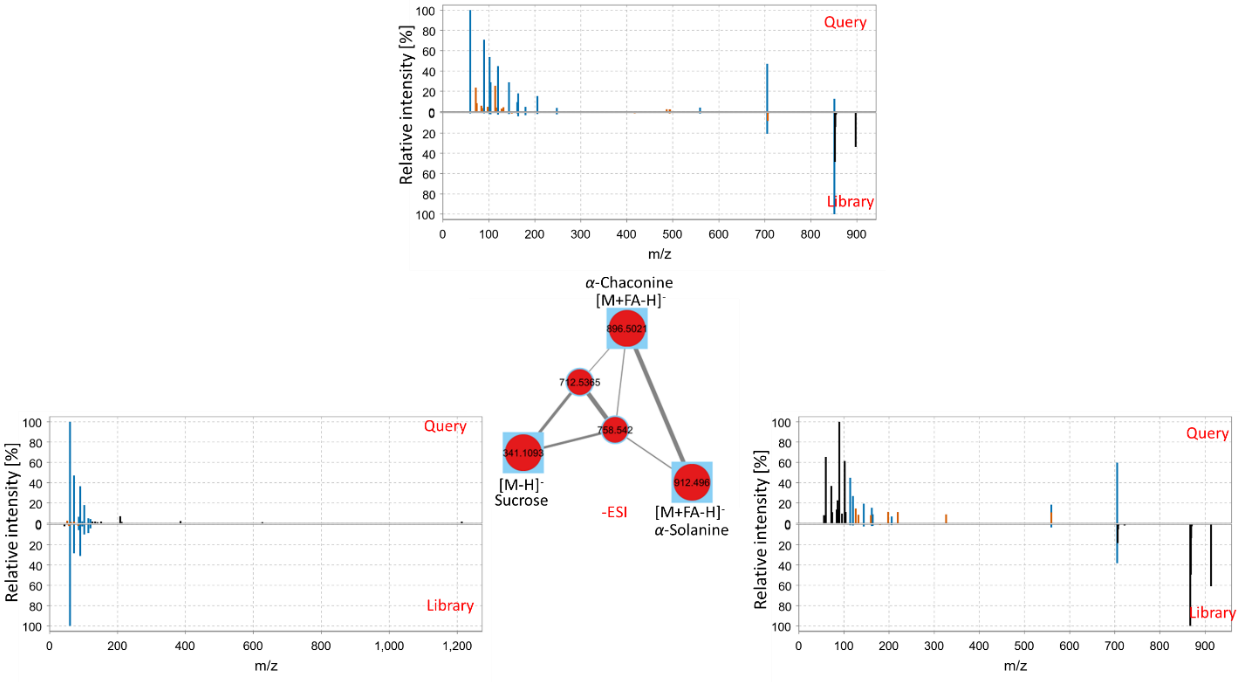

Figure S13. Selected sub-network of alkaloids identified from potato peel extract in negative ionization mode. Exact library matches of metabolites to hits from the MONA public database are shown.

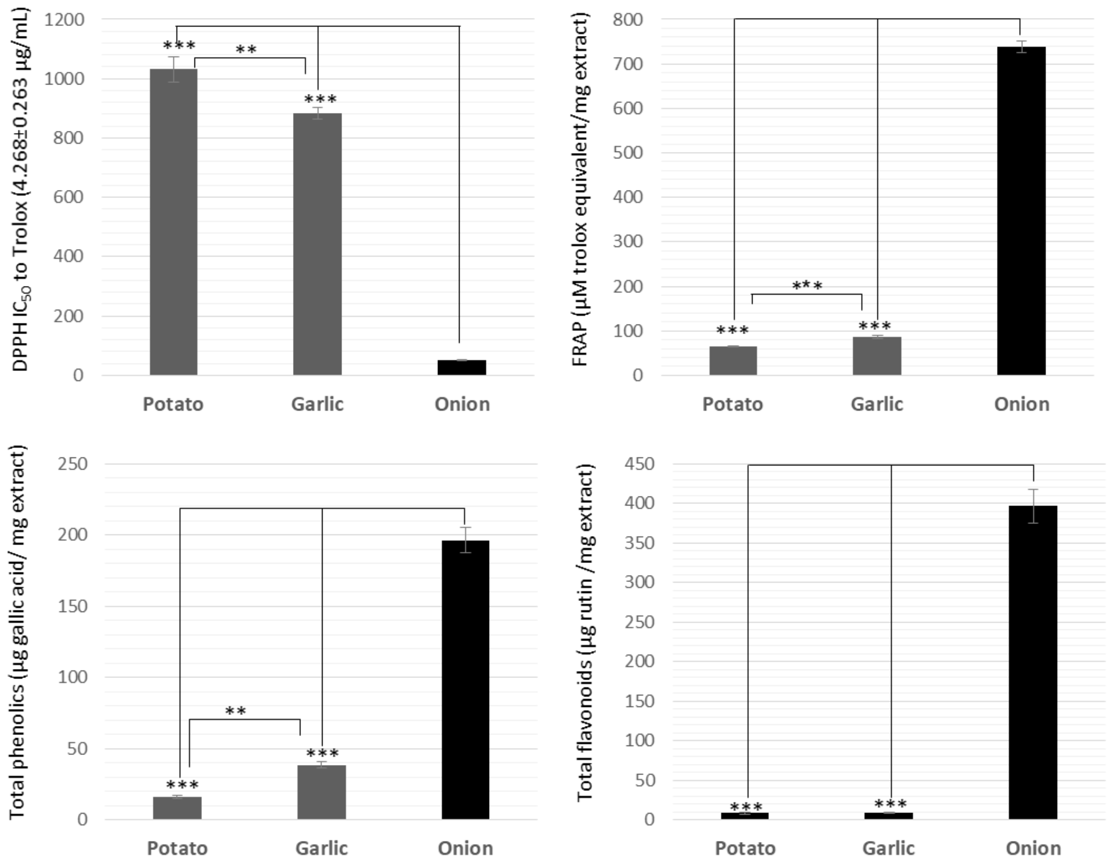

Figure S14. Antioxidant potentiality and phenolics contents of selected peel extracts. Data are presented as the mean ± S.D. of three independent experiments. Asterisks indicate significance level (*, **, ***p<0.05, 0.01 and 0.001, respectively)

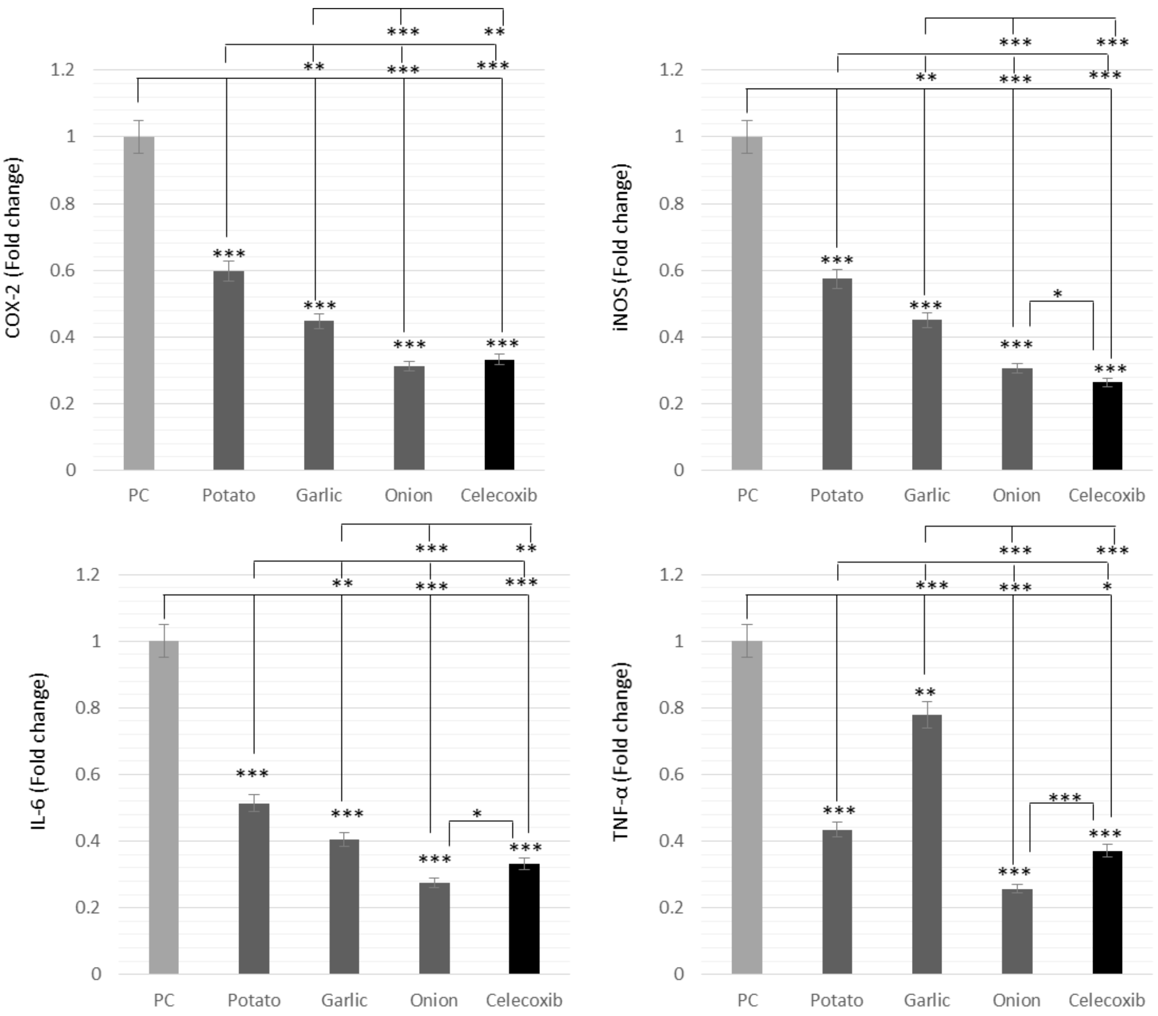

Figure S15. Effects of selected peel extracts on IL-1β-induced inflammatory mediators. Data are presented as the mean ± S.D. of three independent experiments. Asterisks indicate significance level ($^{*,\ **,\ ***}$p<0.05, 0.01 and 0.001, respectively).

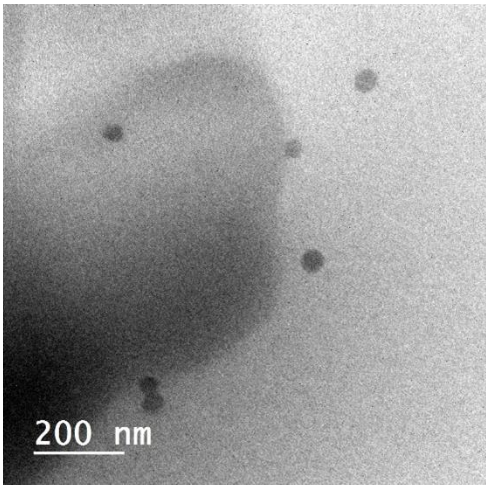

Figure S16. Transmission electron microscope of LNC$_{OPE}$

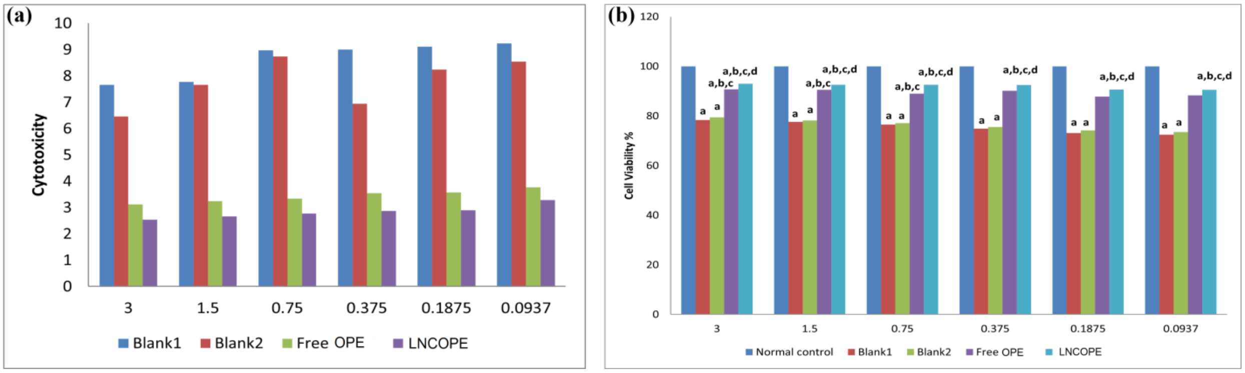

Figure S17. Effect of free OPE and LNC$_{OPE}$ and their blanks on Hfb4 cytotoxicity and cell viability. (a) cytotoxicity and **(b)** cell viability. Data are expressed as the mean ± SD and were analyzed using one-way ANOVA followed by Tukey post hoc test. Values were considered significantly different at $p < 0.01$. a: significant *versus* normal control, b: significant *versus* blank 1, c: significant *versus* blank 2, and d: significant *versus* free OPE.

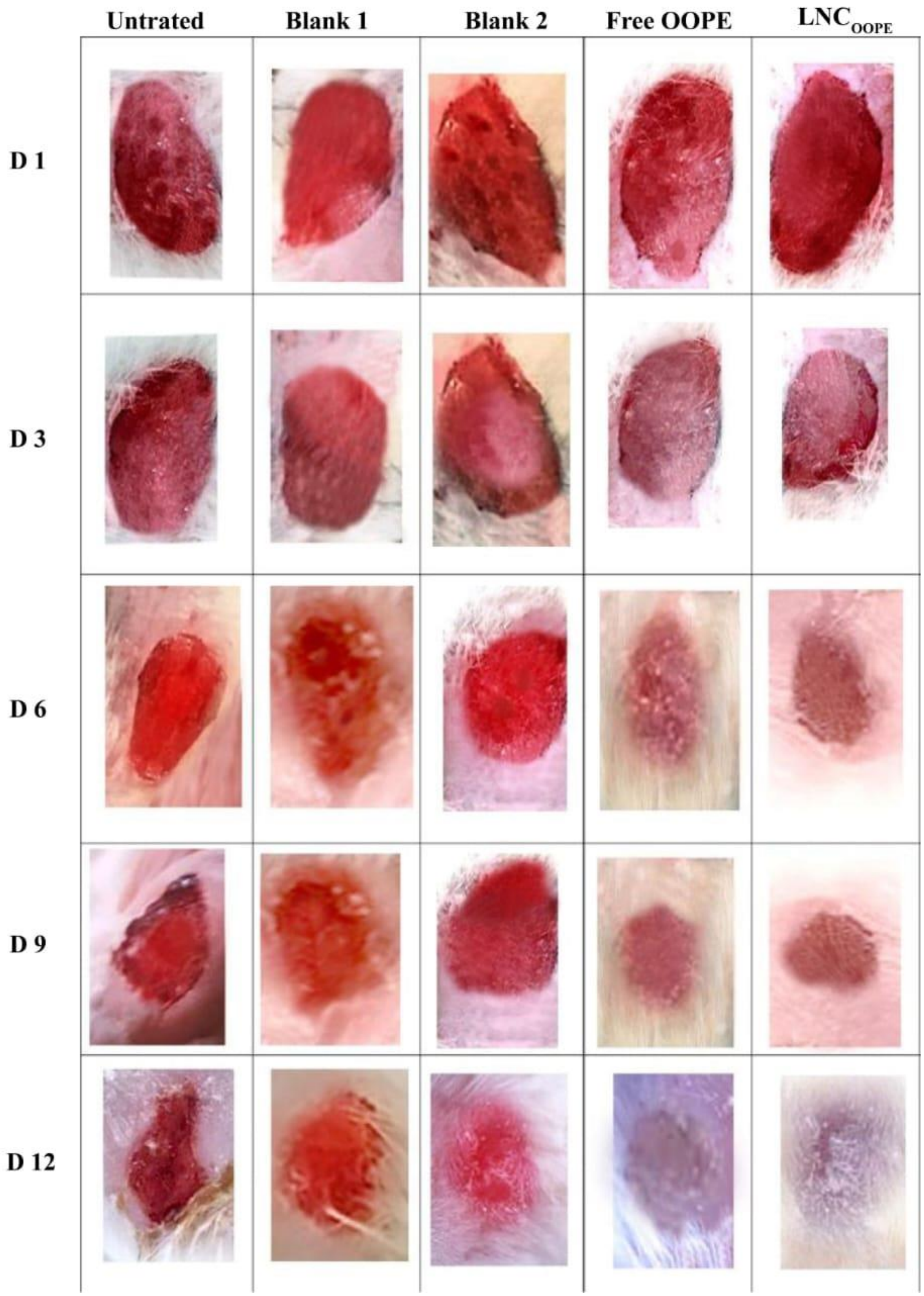

Figure S18. Photographed wound contraction for one representative mouse for each studied group.

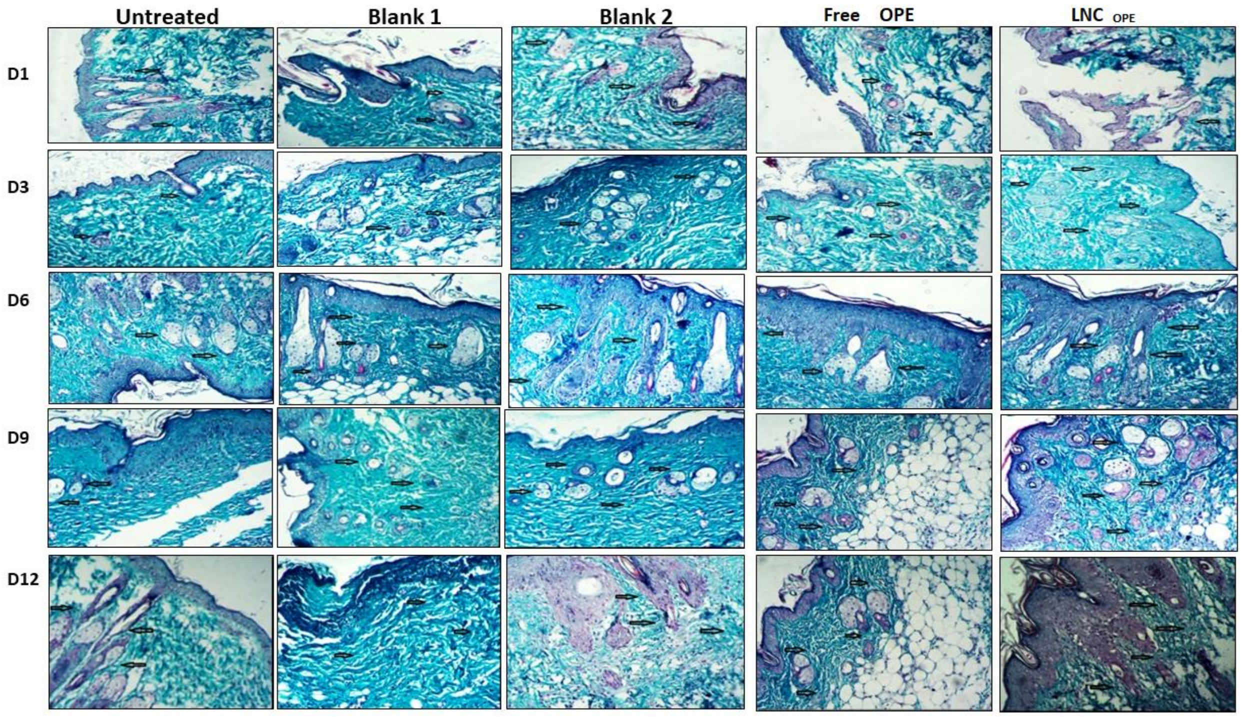

Figure S19. Effect of free OPE and LNC$_{OPE}$ and their blanks on histopathology of the wound tissue stained by MT and visualized at 200x.

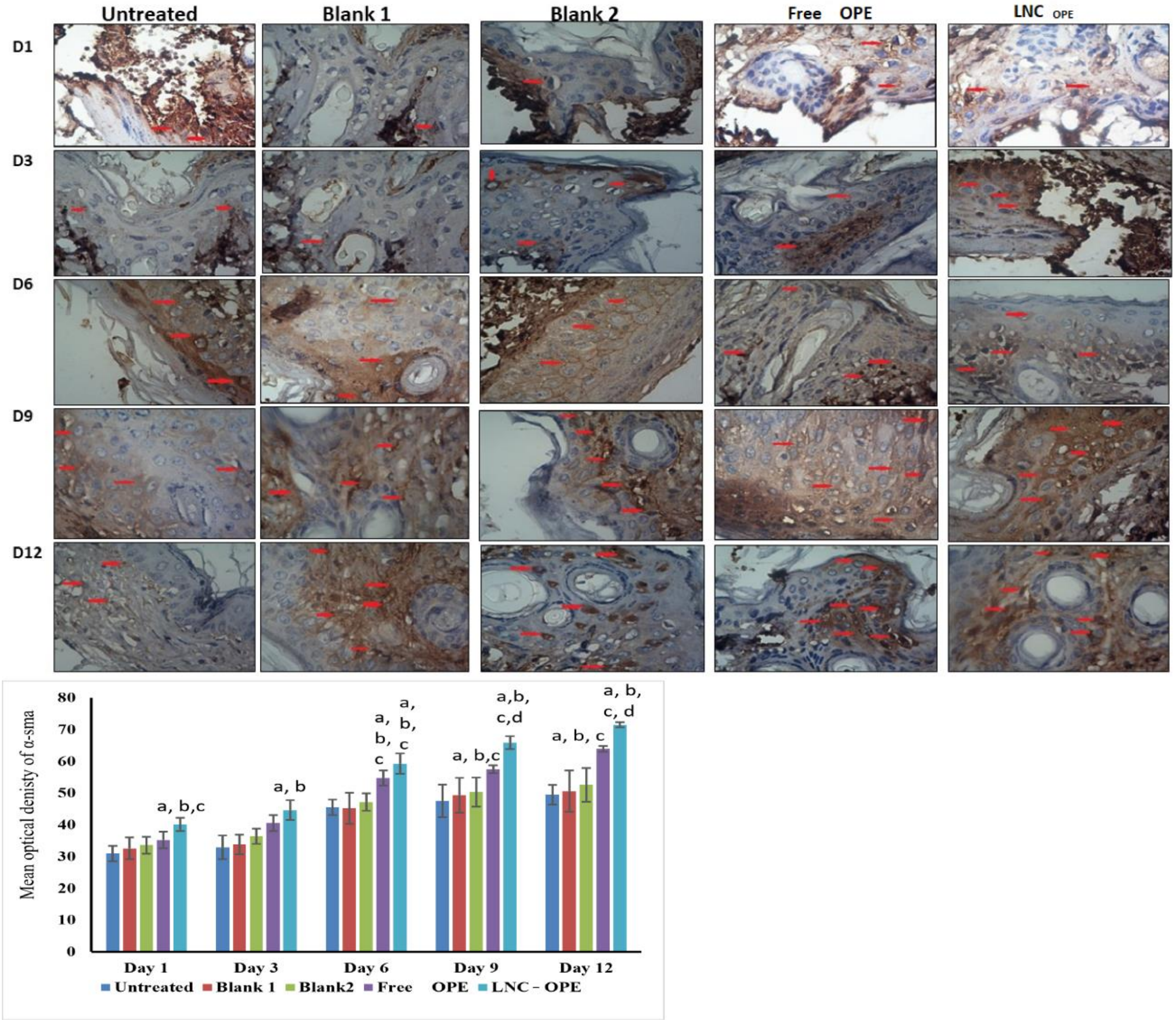

Figure S20. Effect of free OPE and LNC$_{OPE}$ and their blanks on α-SMA immunohistochemistry and the quantified optical density. Data are expressed as the mean ± SD and were analyzed using one-way ANOVA followed by Tukey post hoc test. Values were considered significantly different at $p < 0.01$. a: significant *versus* normal control, b: significant *versus* blank 1, c: significant *versus* blank 2, and d: significant *versus* free OPE. α-SMA: alpha-smooth muscle actin.

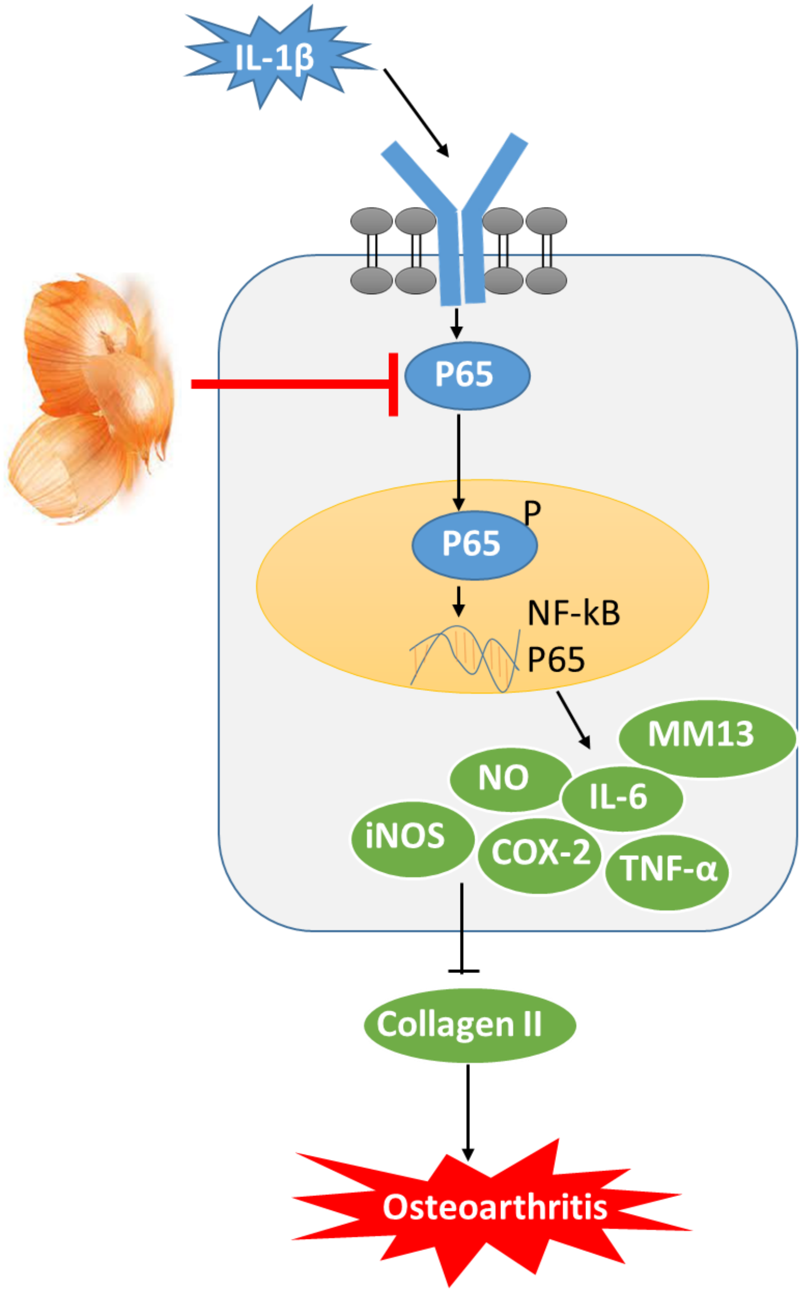

Figure S21. Suggested molecular mechanism of bioactive metabolites from onion peel extract to inhibit IL-1β-mediated inflammation in chondrocytes. The nuclear factor kappa B (NF-κB) signaling pathway is downstream effector of IL-1β. Onion peel extract prevented IL-1β-induced inflammation in mouse-isolated chondrocytes through the NF-kB-dependent downregulation of NO, iNOS, COX-2, IL-6, TNF-α, and MMP-13 production, and thus preventing IL-1β-stimulated ECM degradation.

Table S1. Sequence of the used primers for evaluation of anti-osteoarthritis activity

Gene	Forward	Reverse
Cox2	5'-CGGTGAAACTCTGGCTAGACAG-3’	5'- GCAAACCGTAGATGCTCAGGGA-3'
iNOS	5'-GCTCTACACCTCCAATGTGACC -3’	5'-CTGCCGAGATTTGAGCCTCATG-3'
IL6	5'-AGACAGCCACTCACCTCTTCAG-3’	5'-TTCTGCCAGTGCCTCTTTGCTG-3'
TNFα	5’-CTCTTCTGCCTGCTGCACTTTG-3’	5'-ATGGGCTACAGGCTTGTCACTC-3'
β-actin	5’-GCACCACACCTTCTACAATG-3’	5’-TGCTTGCTGATCCACATCTG-3’

COX-2: cyclooxygenase-2, iNOS: inducible nitric oxide synthase, IL6: Interleukin 6, TNF-α: tumor necrosis factor-α

Table S2. Sequence of the used primers for evaluation of wound healing activities

Gene	Forward	Reverse
ATF-2	CTCTCCTCCGGGGCTAGTTT	ATTCATGGTCCTGCCAGCC
c-Fos	CAACCTGACGGCTTCTCTCTT	GGCAGGTGAGGACAAACGAG
Fra-2	TGATCAAGACCATCGGTACCAC	GCAGCTAGCTTGTTTCTCTCC
c-Jun	CCTATTGGCCGGCAGACTTT	TGCAGTTTGTAACCCCTCCC
GAPDH	TGCCAGGTGAAAATCGCGGA	CACTTCGCACCAGCATCCCT

ATF-2: activating transcription factor 2, c-Fos: Fos proto-oncogene, Fra-2: Fos-related antigen 2, c-Jun: Jun proto-oncogene

Table S3. Tentatively identified compounds in the tested peel extracts obtained by LC-MS/MS analysis.

Compound	RT (min)	m/z (+ESI)	m/z (-ESI)	Chemical formula	Adducts	Onion	Garlic	Potato
Sucrose	0.4	365.1038068	341.1088573	C12H22O11	$[M+Na]^+$, $[M-H]^-$	+++	+++	++
Gallic acid	0.57	-	169.01477	C7H6O5	$[M-H]^-$	++	-	-
3,4-Dihydroxybenzoic acid	0.79	-	153.0190839	C7H6O4	$[M-H]^-$	+++	-	-
Luteolin 7, 4-diglucoside	1.53	611.1603	609.1472	C27H30O16	$[M+H]^+$, $[M-H]^-$	-	-	-
Caffeic acid	1.61	-	179.03391	C9H8O4	$[M-H]^-$	++	-	-
Peonidin-3-O-glucoside	2.21	-	463.12271	C22H23O11+	$[M]^+$	-	-	-
Vanillic acid	2.23	-	167.03465	C8H8O4	$[M-H]^-$	+	-	-
Quercetin-3,4'-O-diglucoside	2.41	627.1558388	625.1416471	C27H30O17	$[M+H]^+$, $[M-H]^-$	+++	+	-
Cyanidin 3-(3''-malonylglucoside)	2.44	535.1087931	-	C24H23O14+	$[M]^+$	-	-	-
Cyanidin 3-(6''-malonylglucoside)-5-glucoside	2.49	697.1611523	-	C30H33O19+	$[M+H]^+$	-	-	-
Spiraeoside (quercetin glucoside)	2.5	465.1033101	463.08804	C21H20O12	$[M+H]^+$, $[M-H]^-$	++	-	-
Isorhamnetin-3,7-di-O-glucoside	2.58	-	639.1566669	C28H32O17	$[M-H]^-$	++	-	-
Quercetin-3-O-galactoside (Hyperoside)	2.8	465.10321	463.08823	C21H20O12	$[M+H]^+$, $[M-H^{]-}$	++	-	-
Dihydroquercetin (Taxifolin)	2.84	305.0653334	303.0502091	C15H12O7	$[M+H]^+$, $[M-H]^-$	-	-	-
Luteolin	2.85	287.05466	285.0405367	C15H10O6	$[M+H]^+$, $[M-H]^-$	-	-	-
Feruloyl dehydrotyramine	2.85	312.1232694	-	C18H17NO4	$[M+H]^+$	-	++	-
Delphinidin 3-sophoroside	2.93	627.1577	-	C27H31O17+	$[M+H]^+$	+	-	-
Alliospiroside C	2.99	725.4102278	-	C38H60O13	$[M+H]^+$	++	-	-
Hesperidin	3.05	-	609.18219	C28H34O15	$[M-H]^-$	-	++	-
Isoquercitrin (quercetin glucoside)	3.08	465.1030893	463.08868	C21H20O12	$[M+H]^+$, $[M-H]^-$	++++	++	-
Kaempferol-3-O-glucoside	3.1	449.1092702	447.0935077	C21H20O11	$[M+H]^+$, $[M-H]^-$	++	-	-

Solasonine	3.14	884.49878	-	C45H73NO16	$[M+H]^+$	-	-	+
Isorhamnetin-3-O-glucoside	3.16	479.1183739	477.1041781	C22H22O12	$[M+H]^+$, $[M-H]^-$	+++	-	-
Quercetin-3-O-glucosyl-6"-acetate	3.3	-	505.0985564	C23H22O13	$[M-H]^-$	-	-	-
α-Solanine	3.3	868.505081	912.4960342	C45H73NO15	$[M+H]^+$, $[M+FA-H]^-$	-	-	+++
α-Chaconine	3.33	852.5102441	896.5020663	C45H73NO14	$[M+H]^+$, $[M+FA-H]^-$	-	-	++++
β-Chaconine	3.35	706.4525505	-	C39H63NO10	$[M+H]^+$	-	-	+++
Coumaroyl tyramine	3.36	284.1264797	-	C17H17NO3	$[M+H]^+$	++	-	-
Feruloyltyramine	3.44	314.1357935	312.123887	C18H19NO4	$[M+H]^+$, $[M-H]^-$	+++	+++	-
2'-O-Galloylquercitrin	3.46	-	599.1036838	C28H24O15	$[M-H]^-$	-	-	-
Quercetin	3.55	303.0502605	301.035754	C15H10O7	$[M+H]^+$, $[M-H]^-$	+++	+++	-
2,3,4-Trihydroxybenzoic Acid	3.58	-	169.0141407	C7H6O5	$[M-H]^-$	-	-	-
Kaempferol	3.9	287.0549605	285.0402782	C15H10O6	$[M+H]^+$, $[M-H]^-$	+++	-	-
Methylquercetin	3.93	317.0653032	315.0509581	C16H12O7	$[M+H]^+$, $[M-H]^-$	+++	-	-
Solanidine	3.96	398.3422959	-	C27H43NO	$[M+H]^+$	-	-	++++
2',4',6'-Trihydroxydihydrochalcone	3.99	-	257.0820296	C15H14O4	$[M-H]^-$	+++	-	-
Solasodine	4.05	414.3370797	-	C27H43NO2	$[M+H]^+$	-	-	++
Phytosphingosine	4.15	318.300906	-	C18H39NO3	$[M+H]^+$	-	++++	-
Alliospiroside B	4.26	-	737.4120314	C39H62O13	$[M-H]^-$	+++	-	-
Dehydrophytosphingosine	4.27	316.2847106	-	C18H37NO3	$[M+H]^+$	-	+++	-
Alliospiroside D	4.48	-	753.4076315	C39H62O14	$[M-H]^-$	++++	-	-
3-Hydroxy-3',4',5,6,7,8-hexamethoxyflavone (Natsudaidain)	4.63	419.1335552	-	C21H22O9	$[M+H]^+$	++	-	-
Sarsasapogenin	4.74	417.3367809	-	C27H44O3	$[M+H]^+$	-	+++	-
Linolenic acid	5.18	279.2311554	-	C18H30O2	$[M+H]^+$	-	+++	-
Ruscogenin	5.21	431.3154391	-	C27H42O4	$[M+H]^+$	+++	-	-

Diosgenin	5.26	415.3210486	-	C27H42O3	$[M+H]^+$	-	++	-
Sarsasapogenin	6.37	417.3362268	-	C27H44O3	$[M+H]^+$	+++	++	-
